
JOURNAL INFORMATION
==============================
NLM Title Abbreviation: Int J Circumpolar Health
Journal ID: Int J Circumpolar Health
NLM Journal ID: 9713056

International Journal of Circumpolar Health

ISSN: 1239-9736
EISSN: 2242-3982
Publisher: Taylor & Francis

ARTICLE INFORMATION
==============================
PMCID: PMC8843355
PMID: 28253734
DOI: 10.1080/22423982.2012.11864611
Article ID: 11864611
Article version: 1
Article version: Version of Record
Subjects: Supplement, Circumpolar Health Supplements

A Comparative Review of Circumpolar Health Systems

Electronic publication date: 2017 Mar 3
Publication date: 2012
Volume: 71
Issue: Suppl 9
First page: 1
Last page: 116
Copyright: © 2012 The Author(s). Published by Taylor & Francis.
Copyright year: 2012
Copyright holder: The Author(s)
License: This is an Open Access article distributed under the terms of the Creative Commons Attribution License (http://creativecommons.org/licenses/by/4.0/), which permits unrestricted use, distribution, and reproduction in any medium, provided the original work is properly cited.
License URL: https://creativecommons.org/licenses/by/4.0/

==============================

CIRCUMPOLAR HEALTH
SUPPLEMENTS

2012 (9)

### A Comparative Review of Circumpolar Health Systems

Edited by
Kue Young
Gregory Marchildon

***International Association of Circumpolar Health Publishers:***
*Danish-Greenlandic Society for Circumpolar Health and Greenland Institute of Health Research, Institute for Circumpolar Health Research, International Network for Circumpolar Health Research, Université Laval/Nasivvik Centre, University of Alaska, Anchorage, University of Manitoba, University of Oulu, University of Tromsø*

***Contributing members:***
*Canadian Society for Circumpolar Health, International Union of Circumpolar Health, Nordic Society for Circumpolar Health, Northern State Medical University, Arkhangelsk, The Komi Branch of the Kirov State Medical Academy*

### EDITORIAL BOARD

**Tiina Ikäheimo**
Centre for Environmental and Respiratory Health Research
University of Oulu
Oulu, Finland
tiina.ikaheimo@oulu.fi

**Jon Øyvind Odland**
Institute of Community Medicine
University of Tromsø
Tromsø, Norway
jon.oyvind.odland@ism.uit.no

**Kue Young**
Dalla Lana School of Public Health
University of Toronto
Toronto, Canada
kue.young@utoronto.ca

**The Circumpolar Health Supplements** is owned, published, and copyrighted © 2012 by the International Association of Circumpolar Health Publishers. All rights reserved. Articles and any other material published represent the opinions of the author(s) and should not be construed to reflect the opinions of the Editorial Board of the Circumpolar Health Supplements.

Circumpolar Health Supplements
International Association of Circumpolar Health Publishers
c/o Institute of Health Sciences, PO Box 5000, FI-90014 University of Oulu, Finland
ijch@oulu.fi
www.ijch.fi

ISSN-L 1797-2361
ISSN 1797-2361 (Print)
ISSN 1797-237X (Online)

Layout Privat-Medi, cover design Elina Aronen-Raappana and Riitta Aittamaa

Circumpolar Health Supplements 2012; 9

*International Association of Circumpolar Health Publishers:*
Danish-Greenlandic Society for Circumpolar Health and
Greenland Institute of Health Research,
Institute for Circumpolar Health Research,
International Network for Circumpolar Health Research,
Université Laval / Nasivvik Centre,
University of Alaska, Anchorage,
University of Manitoba,
University of Oulu,
University of Tromsø

*Contributing members:*
Canadian Society for Circumpolar Health,
International Union of Circumpolar Health,
Nordic Society for Circumpolar Health,
Northern State Medical University, Arkhangelsk,
The Komi Branch of the Kirov State Medical Academy

*Editorial board:*
Tiina Ikäheimo
Centre for Environmental and Respiratory Health Research
University of Oulu
Oulu, Finland
tiina.ikaheimo@oulu.fi

Jon Øyvind Odland
Institute of Community Medicine
University of Tromsø
Tromsø, Norway
jon.oyvind.odland@ism.uit.no

Kue Young
Dalla Lana School of Public Health
University of Toronto
Toronto, Canada
kue.young@utoronto.ca

Original text: Kue Young, Gregory Marchildon

Cover design: Elina Aronen Raappana and Riitta Aittamaa
Layout: Privat-Medi, Finland
Printing: Rannikon Laatupaino, Vihanti, Finland

### A Comparative Review of Circumpolar Health Systems

Edited by
Kue Young
Gregory Marchildon

2012

**Supported by:**

norden
Supported by the Nordic Council of Ministers

Health Canada Santé Canada

CIHR IRSC

### Table of contents

Chapter 1. Background and Overview — Kue Young

1.1 Introduction . . . 7
1.2 Geographical Coverage . . . 7
1.3 Methods and Data Sources . . . 10
1.4 Population Characteristics . . . 10
1.5 Indigenous People . . . 13
1.6 Climatic Conditions . . . 18
1.7 Health Status . . . 19
1.8 Health Care Expenditures . . . 20
1.9 Health Care Facilities . . . 24
1.10 Health Care Personnel . . . 26

Chapter 2. Alaska — Kathryn Anderson

2.1 Introduction . . . 31
2.2 Organizational Structure and Financing . . . 32
2.3 Delivery of Health Services . . . 35
2.4 Patient Pathways . . . 37
2.5 Human Resources Management . . . 38
2.6 Conclusions . . . 40

Chapter 3. Northern Canada — Gregory Marchildon and Susan Chatwood

3.1 Introduction . . . 41
3.2 Organizational Structure and Financing . . . 43
3.3 Delivery of Health Services . . . 46
3.4 Patient Pathways . . . 48
3.5 Emerging Technologies . . . 49
3.6 Human Resources Management . . . 50
3.7 Conclusions . . . 51

Chapter 4. Greenland — Peter Bjerregaard

4.1 Introduction . . . 53
4.2 Organizational Structure and Financing . . . 54
4.3 Delivery of Health Services . . . 54
4.4 Patient Pathways . . . 58
4.5 Health Care Reform . . . 58
4.6 Human Resources Management . . . 59
4.7 Conclusions . . . 60

Chapter 5. Iceland ................................ Tinna Laufey Ásgeirsdóttir

5.1 Introduction .................................................. 63
5.2 Organizational Structure and Financing ........................ 63
5.3 Delivery of Health Services ................................... 66
5.4 Human Resources Management .................................... 69
5.5 Conclusions ................................................... 69

Chapter 6. Northern Norway Jon Magnussen

6.1 Introduction .................................................. 71
6.2 Organizational Structure and Financing ........................ 72
6.3 Delivery of Health Services ................................... 73
6.4 Patient Pathways – the Coordination Reform .................... 77
6.5 Sami Health Services .......................................... 78
6.6 Human Resources Management .................................... 78
6.7 Conclusions ................................................... 79

Chapter 7. Northern Finland Johanna Lammintakanen and Juha Kinnunen

7.1 Introduction .................................................. 81
7.2 Organizational Structure and Financing ........................ 83
7.3 Delivery of Health Services ................................... 85
7.4 Human Resources Management .................................... 87
7.5 Conclusions ................................................... 88

Chapter 8. Cross-Cutting Issues Gregory Marchildon, Jon Magnussen, Kue Young, Peter Bjerregaard, Susan Chatwood, Kathryn Anderson

8.1 Introduction .................................................. 89
8.2 Financing ..................................................... 89
8.3 Decentralization .............................................. 90
8.4 Human Resources ............................................... 91
8.5 Indigenous People ............................................. 92
8.6 Public Health ................................................. 93
8.7 Telehealth Innovations ........................................ 95

Chapter 9. Policy Implications and Future Research Gregory Marchildon and Kue Young

9.1 Health System Challenges ...................................... 99
9.2 Health System Responses ....................................... 101

Abbreviations ..................................................... 103
Bibliography ...................................................... 104
Contributors ...................................................... 111

### PREFACE

The Circumpolar Health Systems Review [CircHSR] is an international collaborative effort to describe and compare the health care systems that exist in the northern regions of Arctic States. The project was proposed by the Arctic Human Health Expert Group and authorized by Senior Arctic Officials of the Arctic Council in April 2010. Funding for the project was provided by the Canadian Institutes of Health Research to the University of Toronto, by Health Canada to the Institute for Circumpolar Health Research, and by the Nordic Council of Ministers to the International Association of Circumpolar Health Publishers. For a variety of reasons, we were unable to secure final contributions from the Faroe Islands, Sweden, and the Russian Federation. However, statistical data from these countries and regions were collected and included in the overview chapter, so that this report can be considered truly circumpolar.

This report consists of a series of narrative and statistical profiles of selected northern regional health care systems, followed by discussion of several cross-cutting issues. A concluding chapter identifies policy implications (but not recommendations) and suggests potential future research. It is intended for wide dissemination among knowledge-user partners, researchers, and stakeholders in regional and national health ministries and agencies, indigenous people's organizations, and international intergovernmental bodies such as the Arctic Council and Northern Forum.

Most of the contributors worked together for the first time. In a remarkable spirit of collaboration, the review took less than two years to complete, from conception to publication. The contributors met face-to-face twice in Copenhagen and once in Toronto, and prepared, exchanged and critiqued drafts. Dr. Tiina Ikäheimo, former Editor-in-Chief of the *International Journal of Circumpolar Health*, deserves special acknowledgment for her tireless efforts in coordinating the review and shepherding it through the publication process. Dr. Winfried Dallmann of the Norwegian Polar Institute drew all the maps, most of which have also appeared in the *Circumpolar Health Atlas* (Young et al, 2012).

It should be noted that this review is meant to be more descriptive than evaluative. We consider our first and foremost task to assemble and present information in a consistent format on the organization, financing, and delivery of health services. It is envisioned that the report will be used as a reference document from which other more in-depth studies on the performance of the different health systems can be based.

### CHAPTER 1.
### BACKGROUND AND OVERVIEW

Kue Young

### 1.1 Introduction

Health care for remote, sparsely populated communities in the circumpolar North faces substantial organizational, financial, logistical, technical, and human resource challenges. Circumpolar regions share many common features but have evolved vastly different health care systems and policies. Identifying, evaluating and comparing approaches to policy development, best practices and successful models across the various regions represent a potentially useful strategy to improve and strengthen northern health systems.

Circumpolar regions are diverse. In this comparative study, the research team has decided to highlight three particular features and examine how they are responsible (or not) in making the northern health care systems different from one another and from the southern regions of the countries to which they belong. These features are (1) remote and sparsely distributed populations, (2) indigenous people's share of the regional population, and (3) harsh climate.

### 1.2 Geographical Coverage

Within the purview of CircHSR are 27 political-administrative regions (**Fig.1.1** and **Table 1.1**). The whole of Alaska, Greenland, Iceland, and Faroe Islands are included. Northern Canada is defined as the three northern territories of Yukon, Northwest Territories and Nunavut. The northernmost "counties" in Norway (*fylke*), Sweden (*län*) and Finland (*lääni*) constitute the northern regions of those countries. In 2010, Finland abolished the *lääni* and replaced it with the regional state administrative agency (*Aluehallintovirasto* or AVI). For the northern regions of Oulu (now called Pohjois-Suomi) and Lappi, there is little impact on boundaries or population served.

The Russian North (*Rossiyskiy Sever*) stretches across the Eurasian landmass. "Siberia" (*Sibir*) tends to be used by people outside Russia to refer to all of Russia east of the Ural Mountains. Within Russia, it has a more restricted meaning geographically which excludes the Far East. The Russian Federation is composed of different types of administrative divisions called federal "subjects" (*subekty*), including republic, *kray*, *oblast*, autonomous *okrug*, and federal city, with varying degrees of autonomy. An autonomous okrug (AO), with the exception of Chukotka, is generally part of some higher level units such as an oblast or kray, and usually represents the traditional territory of some indigenous ethnic group. Statistical data are usually available for these AOs separately. As of January 1, 2007, Taymyr, Evenkia and Koryak AO ceased to exist as distinct federal subjects, and were fully absorbed into the Krasnoyarsk kray and Kamchatka kray, although some statistics continue to be produced for these former AOs.

Although Iceland is an independent country, it is considered a "region" for the purpose of this review when compared with the other subnational regions. Greenland and, to a lesser extent, the Faroe Islands, are not quite fully independent countries, and they too are listed as "regions". It is customary to compare them with Denmark, although in statistical terms, their data are rarely included with Denmark's.

In all, the Arctic regions included in this review encompass 17 million square km, sustaining a sparsely distributed population of just under 10 million inhabitants by the end of the first decade of the 21st century.

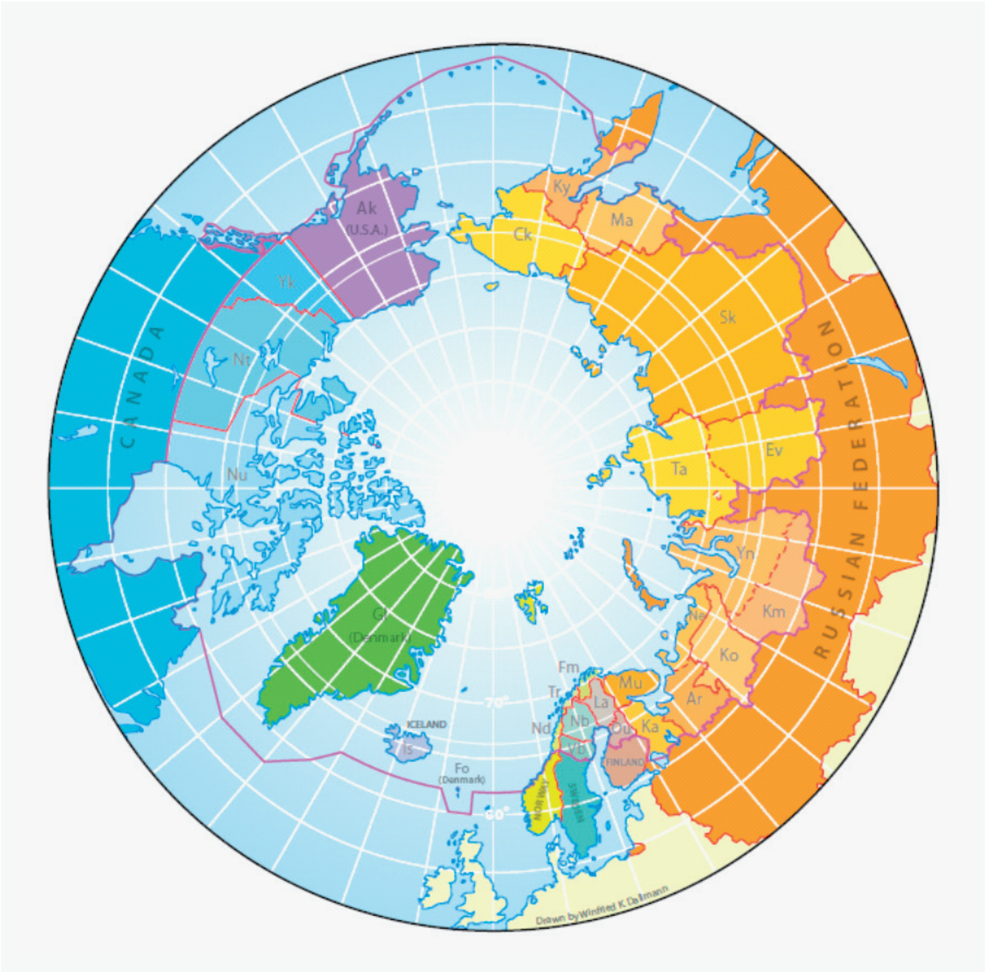

**Figure 1.1.** Map of the circumpolar North and its regions.

**Table 1.1.** Circumpolar countries and regions.

| No. | Abbrev | Name used in Atlas | Status | Name in national official language | Note |
|---|---|---|---|---|---|
| | US | United States of America | | United States of America | |
| 1 | Ak | Alaska | state | Alaska | |
| | CA | Canada | | Canada | |
| 2 | Yk | Yukon | territory | Yukon | |
| 3 | Nt | Northwest Territories | territory | Northwest Territories | Territoires du Nord-Ouest in French |
| 4 | Nu | Nunavut | territory | Nunavut | |
| | DK | Denmark | | Danmark | |
| 5 | Gl | Greenland | *selvstyre* | Kalaallit Nunaat | "self-rule" territory |
| 6 | Fo | Faroe Islands | *hjemmestyre* | Føroyar | "home-rule" territory |
| 7 | IS | Iceland | | Ísland | |
| | NO | Norway | | Norge | |
| 8 | Nd | Nordland | *fylke* | Nordland fylke | |
| 9 | Tr | Troms | *fylke* | Troms fylke | Romsa in Sami |
| 10 | Fm | Finnmark | *fylke* | Finnmark fylke | Finnmárku in Sami |
| | SE | Sweden | | Sverige | |
| 11 | Vb | Västerbotten | *län* | Västerbottens län | |
| 12 | Nb | Norrbotten | *län* | Norrbottens län | |
| | FI | Finland | | Suomi | |
| 13 | Ou | Oulu | *lääni* | Oulun lääni | Uleåborg in Swedish |
| 14 | La | Lappi | *lääni* | Lapin lääni | Lappland in Swedish |
| | RU | Russian Federation | | Rossiyskaya Federatsiya | |
| 15 | Mu | Murmansk Oblast | *oblast* | Murmanskaya oblast | |
| 16 | Ka | Kareliya Republic | republic | Respublika Kareliya | |
| 17 | Ar | Arkhangelsk Oblast | *oblast* | Arkhangelskaya oblast | includes also Nenets AO |
| 18 | Ne | Nenets AO | autonomous okrug | Nenetskiy avtonomnyy okrug | part of Arkhangelsk Oblast |
| 19 | Ko | Komi Republic | republic | Respublika Komi | |
| 20 | Yn | Yamalo-Nenets AO | autonomous *okrug* | Yamalo-Nenetskiy avtonomnyy okrug | |
| 21 | Km | Khanty-Mansi AO | autonomous *okrug* | Khanty-Mansiyskiy avtonomnyy okrug | also known as Yugra |
| 22 | Ta | Taymyr AO | autonomous *okrug* | Taymyrskiy avtonomnyy okrug | also known as Dolgan-Nenets AO |
| 23 | Ev | Evenkia AO | autonomous *okrug* | Evenkiyskiy avtonomnyy okrug | |
| 24 | Sk | Sakha Republic | republic | Respublika Sakha | also known as Yakutiya |
| 25 | Ma | Magadan Oblast | *oblast* | Magadanskaya oblast | |
| 26 | Ky | Koryak AO | autonomous *okrug* | Koryakskiy avtonomnyy okrug | |
| 27 | Ck | Chukotka AO | autonomous *okrug* | Chukotskiy avtonomnyy okrug | |

Note: The regions listed in Table 1.1 and shown in Fig.1.1 were in existence during much of the decade 2000-2009. See text for administrative changes in Finland and Russia that resulted in some of these regions being renamed or absorbed into larger entities.

The 2-letter country and region codes are used throughout this report in tables and figures. Some charts also include data on Alaska Natives (Ak-Nat) and individuals born in Greenland (Gl-born) to represent the indigenous population within the respective regions.

### 1.3 Methods and Data Sources

This report is based on a review of the literature and publicly available statistical data produced by national and regional governments and health care agencies. While no primary data collection in the form of interviews of key informants or direct observation was conducted, the CircHSR project team members have drawn upon their many years of experience as researchers, administrators, clinicians, consultants, and advisors, and are well connected with decision-makers and stakeholders in the various national and regional health systems.

In order to compare national health systems using indicators such as health expenditures, personnel, and facilities, data were obtained from international organizations such as the Organization of Economic Cooperation and Development (OECD), the European Statistical Agency (EUROSTAT), the World Health Organization (WHO) and the Nordic Medical-Statistical Committee (NOMESCO). Considerable efforts have been made by such agencies in achieving international comparability of concepts and definitions, although with varying degree of success.

For within-country comparisons between "the North" and the country as a whole, or between northern and southern regions, the data sources are usually from the specific country and the definitions and concepts may be unique to that country. For this reason, while it is appropriate to compare northern Norway with Norway as a whole, and northern Canada with Canada as a whole, for example, great caution must be exercised when comparing northern Norway with northern Canada. The narrative sections of the profiles provide the necessary context in which to interpret the statistical data.

An overview of selected statistical indicators relating to the population, health status, and health system characteristics of circumpolar regions is provided here to highlight the diversity among them. More extensive coverage of health indicators is provided by the interactive web-based Circumpolar Health Observatory [http://circhob.circumpolarhealth.org] established and maintained by the Institute for Circumpolar Health Research in Yellowknife, Canada.

### 1.4 Population Characteristics

Circumpolar regions differ in the size of their population, their share of the total national populations, and population density (Young & Bjerregaard, 2008) (**Table 1.2**). Alaska and the northern territories of Canada constitute less than 0.5% of the total population of the United States and Canada respectively. Greenland's population is only 1% that of Denmark's. In contrast, a much higher proportion of the population of Norway (10%), Sweden (6%) Finland (12%), and Russia (5%) reside in their northern regions.

Between 1990 and 2009, Alaska, northern Canada and Iceland witnessed substantial population growth, while many northern Russian regions suffered depopulation, with some losing more than half its population in 20 years. In only Yamalo-Nenets AO and Khanty-Mansi AO, which experienced large scale oil and gas development, did the population increase. Greenland and the northern regions of Fennoscandia experienced population change of less than ±10%.

**Table 1.2.** Mean annual population 1990-2009 and population density.

| Country/Region | 1990 | 2000 | 2009 | % change 2009/1990 | Density (persons/sq km) |
|---|---|---|---|---|---|
| United States | 249622814 | 282171957 | 307006550 | 23.0 | 33.5 |
| Alaska | 553290 | 627499 | 698473 | 26.2 | 0.47 |
| Canada | 27697530 | 30685730 | 33729690 | 21.8 | 3.7 |
| Yukon | 27774 | 30431 | 33683 | 21.3 | 0.07 |
| Northwest Territories | 37330 | 40480 | 43638 | 16.9 | 0.04 |
| Nunavut | 21580 | 27498 | 32240 | 49.4 | 0.02 |
| *N. Canada* | *86684* | *98409* | *109561* | *26.4* | *0.03* |
| Denmark | 5140939 | 5337344 | 5519441 | 7.4 | 128.1 |
| Greenland | 55589 | 56176 | 56323 | 1.3 | 0.03 |
| Faroe Islands | 47559 | 45749 | 48635 | 2.3 | 34.7 |
| Iceland | 254788 | 281154 | 319246 | 25.3 | 3.1 |
| Norway | 4241473 | 4490967 | 4828726 | 13.8 | 15.9 |
| Nordland | 239468 | 238702 | 235826 | -1.5 | 6.5 |
| Troms | 146705 | 151469 | 156024 | 6.4 | 6.3 |
| Finnmark | 74369 | 74073 | 72674 | -2.3 | 1.6 |
| *N. Norway* | *460542* | *464244* | *464523* | *0.9* | *4.4* |
| Sweden | 8558835 | 8872110 | 9298515 | 8.6 | 21.1 |
| Västerbotten | 251054 | 256177 | 258180 | 2.8 | 4.7 |
| Norrbotten | 263289 | 257168 | 249348 | -5.3 | 2.5 |
| *N. Sweden* | *514343* | *513345* | *507528* | *-1.3* | *3.3* |
| Finland | 4986431 | 5176209 | 5338871 | 7.1 | 17.6 |
| Oulu | 442004 | 457573 | 473543 | 7.1 | 8.3 |
| Lappi | 200324 | 193060 | 183856 | -8.2 | 2.0 |
| *N. Finland* | *642328* | *650633* | *657399* | *2.3* | *4.4* |
| Russian Federation | 147969414 | 146596870 | 141909244 | -4.1 | 8.3 |
| Murmansk Oblast | 1190127 | 931969 | 839562 | -29.5 | 5.8 |
| Kareliya Republic | 791589 | 732138 | 685856 | -13.4 | 3.8 |
| Arkhangelsk Oblast | 1572231 | 1379726 | 1258243 | -20.0 | 2.1 |
| Nenets AO | 51830 | 41053 | 42158 | -18.7 | 0.2 |
| Komi Republic | 1244388 | 1050377 | 954850 | -23.3 | 2.3 |
| Yamalo-Nenets AO | 488869 | 497282 | 545089 | 11.5 | 0.7 |
| Khanty-Mansi AO | 1273585 | 1371548 | 1529289 | 20.1 | 2.9 |
| Taymyr AO | 51316 | 38257 | 36841 | -28.2 | 0.04 |
| Evenkia AO | 24148 | 18241 | 16368 | -32.2 | 0.02 |
| Sakha Republic | 1115232 | 959993 | 949550 | -14.9 | 0.3 |
| Magadan Oblast | 387401 | 197960 | 162109 | -58.2 | 0.4 |
| Koryak AO | 37666 | 26238 | 20732 | -45.0 | 0.1 |
| Chukotka AO | 160096 | 59574 | 49056 | -69.4 | 0.1 |
| *N. Russia* | *8336644* | *7263303* | *7047543* | *-15.5* | *0.8* |
| Total Northern Regions | 10951766 | 10000512 | 9909230 | -9.5 | 0.6 |

Source: CircHOB.

The population density (number of persons per km$^2$), shown in **Fig.1.2 and Table 1.2**, varies from 0.02 in Nunavut to 35 in the Faroe Islands. Within the sparsely populated regions, the largest city tends to account for a high proportion of the total regional population, ranging from 20% to 70% (**Table 1.3**). An increasing trend towards urbanization is widely observed across the Arctic. From a service delivery perspective, it is often not appreciated that the majority of northern residents are served by a predominantly urban type of health care delivery.

The age-sex structure differs across regions. In general, the population of northern Fennoscandia tends not to differ substantially from their respective national populations, and is characterized by a high proportion of the elderly (65+), between 14% and 18%. The highest proportion of the under-15 population is found in Nunavut (about 35%) and the Northwest Territories, Greenland, and Alaska (about 25%), all regions with a high proportion of indigenous people. The proportion of the elderly is substantially lower in the North, even among the non-indigenous population, reflecting the transient nature of many residents who migrate to the North for occupational reasons. In Russia, the population of the Sakha Republic and the AOs of the Far North tend to be more youthful than those in the European North.

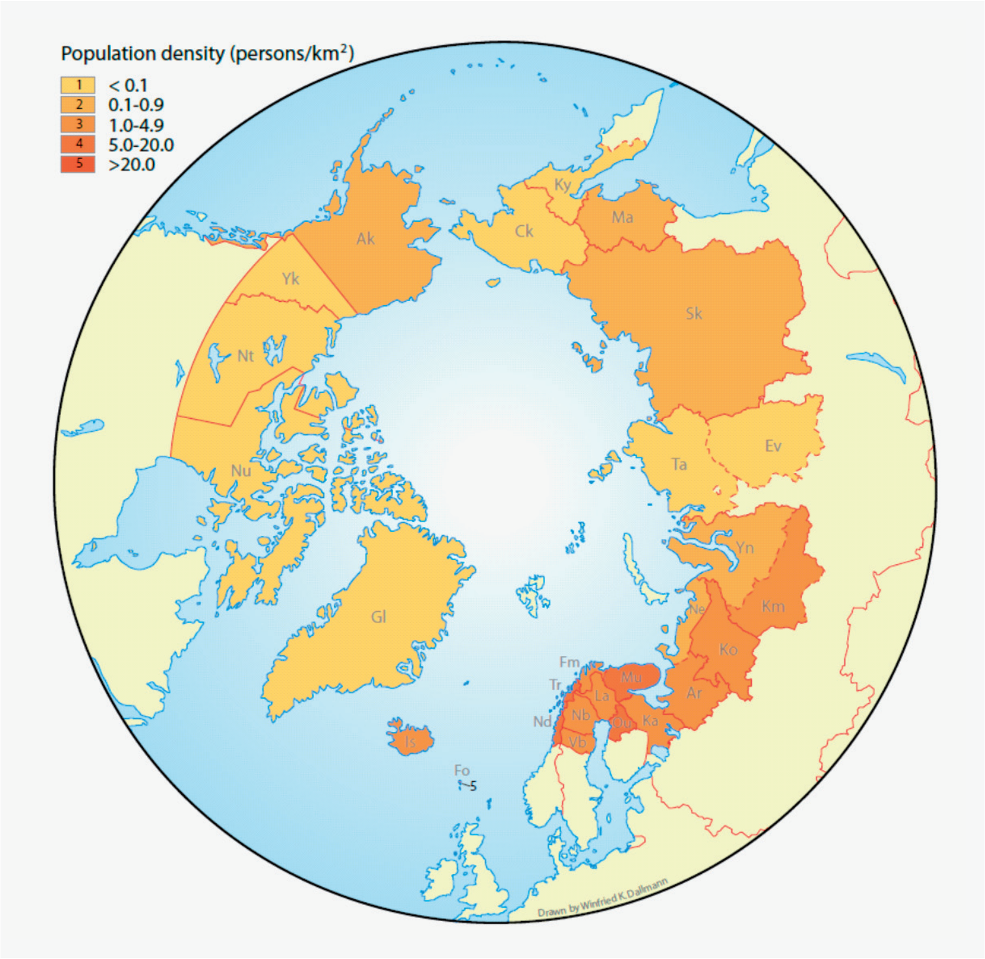

**Figure 1.2.** Map of population density in circumpolar regions. Source: CircHOB.

**Table 1.3.** Major cities in circumpolar regions.

| Region | Name | Status | Latitude North | Mid-decade Total | Population % region |
|---|---|---|---|---|---|
| Alaska | Anchorage | borough [largest city] | 61° 13' | 279940 | 41 |
| | Juneau | borough [admin centre] | 58° 21' | 30680 | 5 |
| Yukon | Whitehorse | city | 61° 43' | 20460 | 63 |
| Northwest Territories | Yellowknife | city | 62° 27' | 18700 | 43 |
| Nunavut | Iqaluit | city | 63° 45' | 6180 | 20 |
| Greenland | Nuuk | by | 64° 10' | 14660 | 26 |
| Faroe Islands | Tórshavn | *kommunur* | 62° 01' | 19320 | 40 |
| Iceland | Reykjavík | *sveitarfélag* | 64° 08' | 116090 | 38 |
| Nordland | Bodø | *kommune* | 67° 18' | 45280 | 19 |
| Troms | Tromsø | *kommune* | 69° 41' | 64040 | 42 |
| Finnmark | Alta | *kommune* [largest city] | 69° 56' | 17990 | 25 |
| | Vadsø | *kommune* [admin centre] | 70° 05' | 6120 | 8 |
| Västerbotten | Umeå | *kommune* | 63° 50' | 111000 | 43 |
| Norrbotten | Luleå | *kommune* | 65° 36' | 73030 | 29 |
| Oulu | Oulu | *kunta* | 65° 01' | 132960 | 28 |
| Lappi | Rovaniemi | *kunta* | 66° 30' | 57970 | 31 |
| Murmansk Oblast | Murmansk | *gorod* | 68° 59' | 319240 | 37 |
| Kareliya Republic | Petrozavodsk | *gorod* | 61° 47' | 265670 | 38 |
| Arkhangelsk Oblast | Arkhangelsk | *gorod* | 64° 32' | 355200 | 28 |
| - Nenets AO | Naryan-Mar | *gorod* | 67° 38' | 26300 | 63 |
| Komi Republic | Syktyvkar | *gorod* | 61° 40' | 244650 | 25 |
| Yamalo-Nenets AO | Novy Urengoy | *gorod* [largest city] | 66° 05' | 114740 | 21 |
| | Salekhard | *gorod* [admin centre] | 66° 32' | 40030 | 7 |
| Khanty-Mansi AO | Surgut | *gorod* [largest city] | 61°16' | 290210 | 20 |
| | Khanty-Mansiysk | *gorod* [admin centre] | 61° 00' | 61400 | 4 |
| Taymyr AO | Dudinka | *gorod* | 69° 24' | 27070 | 70 |
| Evenki AO | Tura | *poselok gorodskogo tipa* | 64° 17' | 5670 | 33 |
| Sakha Republic | Yakutsk | *gorod* | 62° 02' | 263450 | 28 |
| Magadan Oblast | Magadan | *gorod* | 59° 34' | 107440 | 63 |
| Koryak AO | Palana | *poselok gorodskogo tipa* | 59° 05' | 3880 | 17 |
| Chukotka AO | Anadyr | *gorod* | 64° 44' | 11370 | 23 |

Note: Where only one city is listed, it is both the largest city and the administrative centre. Some regions have more than one large city, for example, Fairbanks in Alaska has a population intermediate between that of Anchorage and Juneau.
Source: CircHOB

### 1.5 Indigenous People

The distribution of the various ethnic groups inhabiting the Arctic is shown in **Fig.1.3**. There is no universally accepted definition of indigenous people, not even in the United Nations Declaration on the Rights of Indigenous Peoples. Some circumpolar countries have laws that define and recognize specific groups as indigenous.

The Constitution of Canada identifies First Nations, Inuit and Métis as "Aboriginal". Where land claims agreements have been signed with specific indigenous groups, it is "beneficiary" status that defines whether someone is indigenous or not.

In the United States a variety of treaties and agreements define "American Indians and Alaska Natives" (AIAN). Within these broad legal categories are many different tribal and cultural groups. In Alaska the Alaska Native Claims Settlement Act of 1971 created regional

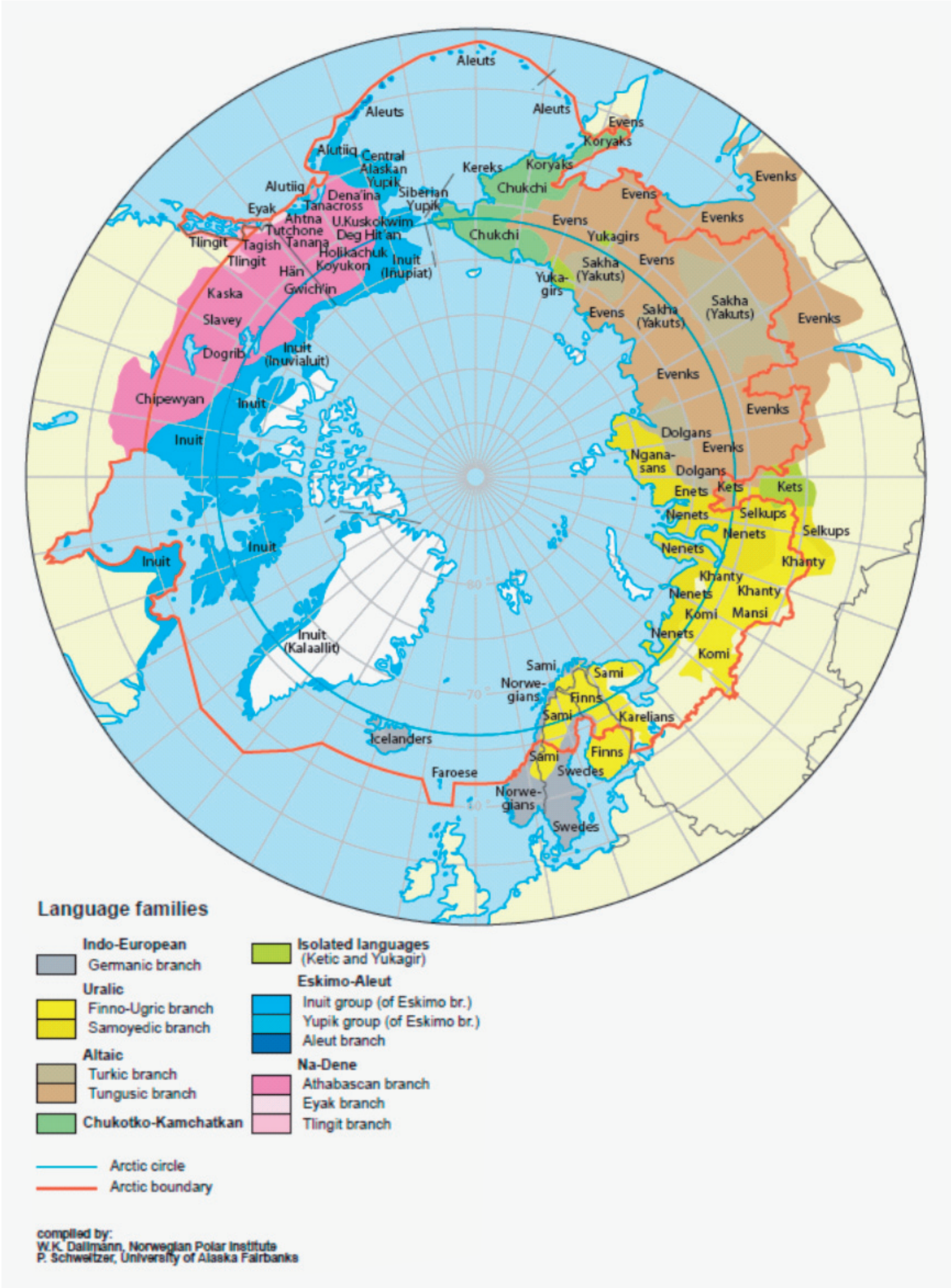

**Figure 1.3.** Map of ethno-linguistic groups in circumpolar regions.

Native (for-profit) corporations and smaller village corporations, and membership in such corporations also defines someone as indigenous.

Since the 1920s the Soviet government had accorded various ethnic groups with population less than 50,000 the status of "numerically small peoples (*korennye malochislennye narody*) of the North, Siberia and the Far East". At the dissolution of the Soviet Union, there were 26 such groups in Russia. By 2005 the number had grown to 41. Excluded are non-Slavic national minorities such as Yakuts, Buryats, and Komi, which have sizable populations in the hundreds of thousands and form their own republics.

The distinction between "national minorities" and "indigenous peoples" is also made in countries such as Norway, where the Finnish-speaking Kvens are considered a minority but not indigenous. Only the Sami are considered indigenous in Norway, Sweden and Finland, where Sami parliaments have been established to protect and promote their cultural identity and indigenous rights. While generally not considered as "indigenous", Icelanders and Faroese can arguably be considered also "indigenous" in the sense of their history of colonization by Denmark, their historical occupancy of their lands, and strong cultural identity.

While ethnic identity is included in the Canadian, United States and Russian censuses, such information is not recorded in the population registries of the Nordic countries. In terms of health status or health care data, only the United States consistently provides health data on their indigenous population. Official agencies in Greenland and researchers have used "born in Greenland" as a marker for indigenous Greenlanders who are Inuit. This practice is far from ideal, and can be expected to be less and less accurate as travel and migration between Greenland and Denmark increases. For the northern territories of Canada, ethnic-specific data are not consistently available, and when available, not usually published.

### *Alaska*

In the 2010 United States Census, 138,312 persons self-identified as AIAN "alone or in any combination", which accounted for 19.5% of the state's total population. Some 16,665 further identified themselves as Athabaskan, 11,216 as Aleut, 25,687 as Inupiat, and 30,868 as Yupik, alone or in combination with some other group (U.S.Census Bureau, 2011a). Many individuals did not provide further subdivision beyond their identity as AIAN. Aleut, Inupiat and Yupik are all part of the Eskimo-Aleut family of languages. Inupiat is part of the Inuit branch which extends from Alaska across Canada to Greenland. Yupik languages are spoken in Alaska and Chukotka in Russia. American Indians of the Northwest Coast culture – Tlingit, Haida, and Tsimshian – also make their home in southeastern Alaska.

### *Northern Canada*

In the 2006 Census of Canada, 7,810 persons in Yukon, 20,905 in the Northwest Territories, and 25,165 in Nunavut reported having Aboriginal ancestry, accounting for 26%, 51% and 86% of the total population of the respective territories. With the three territories combined, 20,580 individuals reported some First Nations ancestry, 2,595 some Métis ancestry, and 28,795 some Inuit ancestry (Statistics Canada, 2011).

### *Greenland*

The population registry of Greenland shows that the population of Greenland in January 2011 was 56,615, of whom 89% were born in Greenland (Statistics Greenland, 2011). Statistics Greenland also reports that in 2007, 13,4582 Greenlanders were resident in Denmark (Statistics Greenland, 2010).

### *Northern Fennoscandia*

There is no direct means to estimate the number of Sami in any of the three countries. Different criteria based on language, ancestry, occupation, and self-identity can be used. One set of figures - 40,000 in Norway, 20,000 in Sweden and 7,500 in Finland – is widely quoted, for example in the *Sami Handbook* (Solbakk, 2006) and various Sami institutional websites. In Russia there were 1,991 Sami enumerated in the 2002 census (Rosstat, 2006). In terms of proportion of the regional population, it could be as high as 30% in Finnmark, and 5% or less in the other regions. Hassler et al (2005) developed

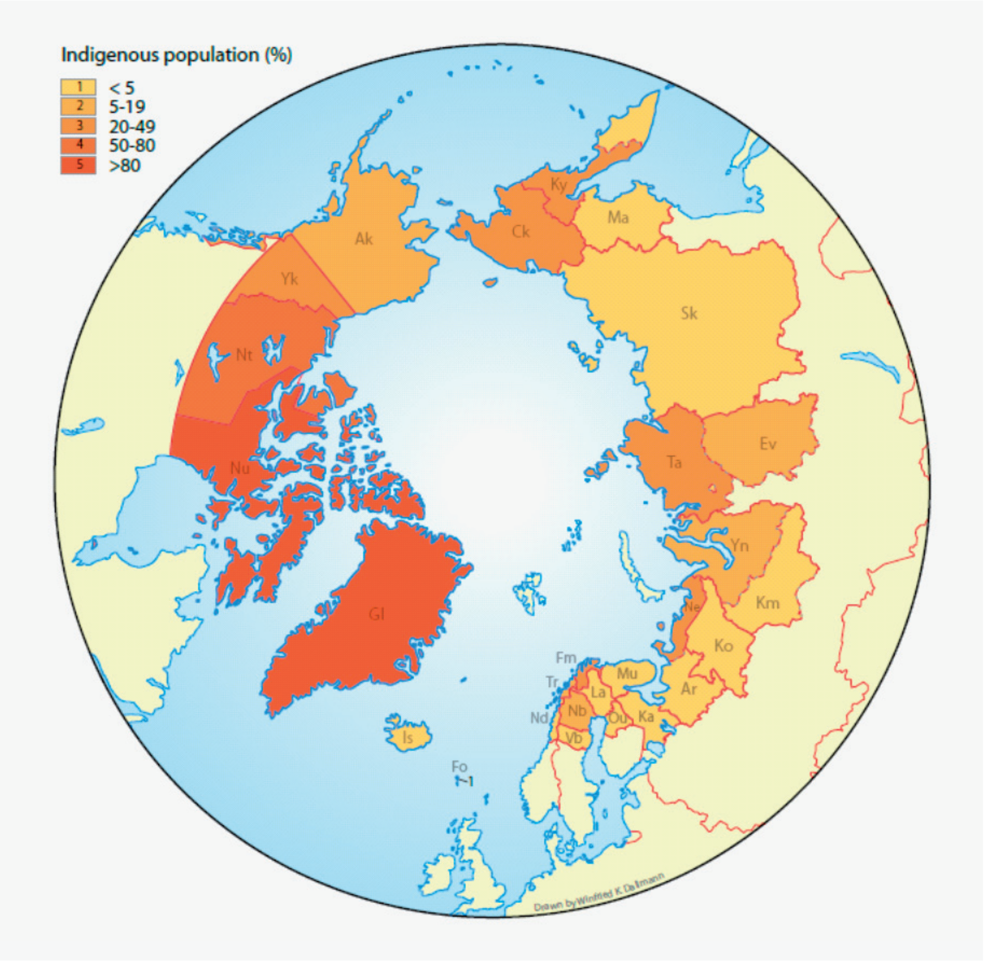

**Figure 1.4.** Proportion of indigenous people in total regional population.

a population database for Swedish Sami using a more inclusive definition, which gives a much higher estimate of 36,000 in Sweden, with 6,000 in Västerbotten and 18,000 in Norrbotten.

In Norway, about one-third of the country's Sami population live in Finnmark (*Finnmárku*), particularly in Kautokeino, Karasjok, Tana, Nesseby, and Porsanger municipalities. In Sweden, Sami are concentrated in various municipalities in Norbotten and Västerbotten, especially Jokkmokk, Arjeplog, Gällivare and Kiruna. In Finland, the Sami region consists of the municipalities of Enontekiö, Inari, Utsjoki, and the northern part of Sodankylä, all within Lapland. Overall the Sami constitutes about one-third of the population of these municipalities, but it is only in Utsjoki that they constitute the majority.

### *Russia*

According to the 2002 Census, there were 160,780 individuals in the 13 northern regions who belonged to any of the 41 designated "numerically small peoples" (Rosstat, 2006).

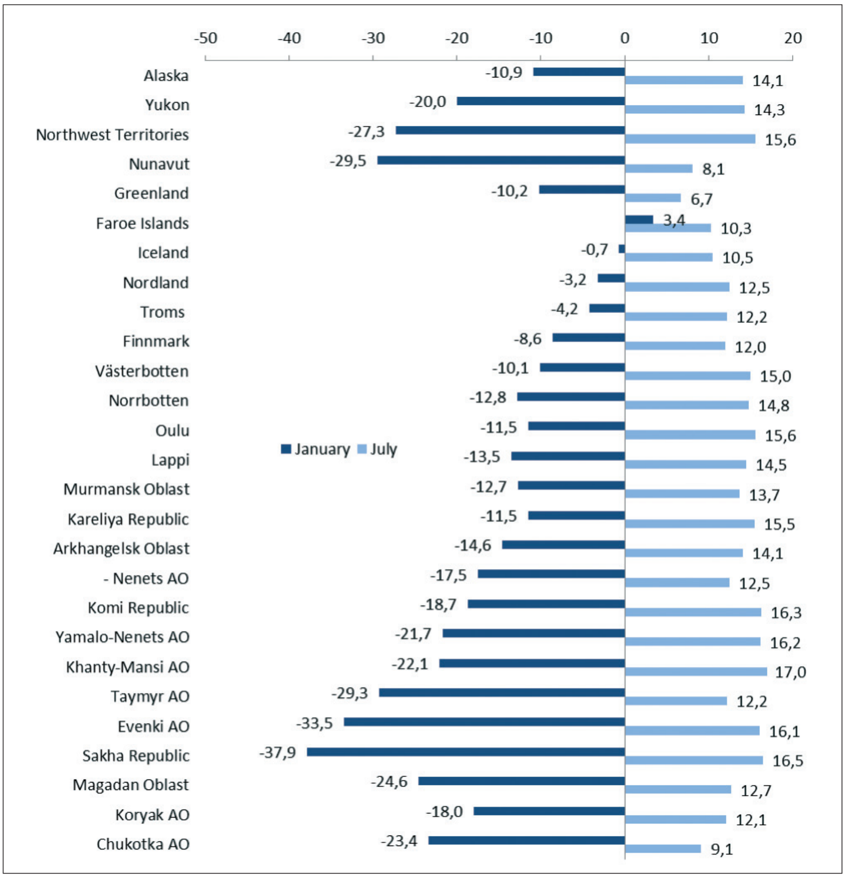

**Figure 1.5.** Mean January and July temperature of circumpolar regions.
Note: Mean temperatures refer to the "climatological normals" as defined by the World Meteorological Organization based on the period 1961-1990, and weighted by population of the municipality in which the weather station is located. Source: Young and Mäkinen (2010).

Their share of the total regional population is highest in the autonomous okrugs – 19% in the Nenets AO, 25% in the Taymyr AO, 41% in the Koryak AO, 10% in Evenkia AO, and 31% in Chukotka AO, but much less in the Yamalo-Nents (7%) and Khanty-Mansi AO (2%). In all other regions the proportion did not exceed 3%. In the whole of the Russian North, only 2% of the population is indigenous.

In summary, it is only in a few jurisdictions that indigenous peoples form a majority (Northwest Territories, Nunavut, and Greenland) of the regional population (Fig.1.4).

### 1.6 Climatic Conditions

Climate is relevant to health as it constitutes part of the physical environment to which all human beings are exposed. Climate also affects the health care delivery system, for example the construction and maintenance of health care facilities and medical trans-

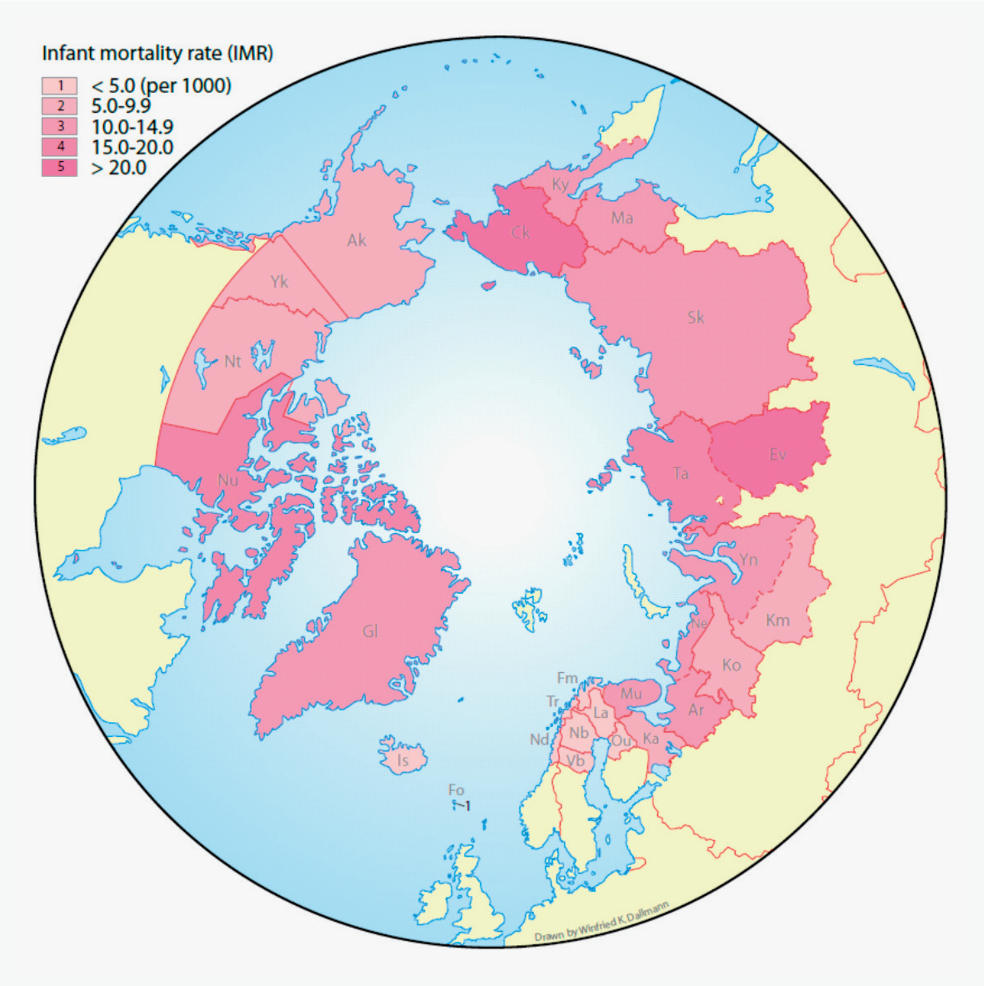

**Figure 1.6.** Infant mortality rate (per 1,000 livebirths) in circumpolar regions. Source: CircHOB.

portation. Although the Arctic is undoubtedly cold, it is not uniformly so. Within the 27 regions, the type of climate includes the polar, the continental and even the temperate, based on temperature, precipitation and native vegetation. Climatic variation exists due to mountain barriers, proximity to open waters, extent of surface snow and ice cover, and the duration of daylight and darkness. **Fig.1.5** compares the mean January and July temperatures of the circumpolar regions, weighted by the size of the population experiencing that climate. Winter temperatures decrease as one moves eastwards across Siberia and reach their lowest in the northeast, where temperatures as low as –70°C have been recorded.

### 1.7 Health Status

The health status of a population can be represented - to use an Arctic analogy – by an iceberg. Above the water, representing the most severe consequences of ill health, is mortality. A commonly used summary health indicator in international comparisons is the infant mortality rate (**Fig.1.6**) and life expectancy at birth ($LE_0$) (**Fig.1.7**). As an example of inter-regional health disparities, the infant mortality rate during the period 2005-09 in the worst region (Koryakia in Russia, 28/1000 livebirths) is 14 times that of the best region (Iceland, 2/1000 livebirths). The difference in $LE_0$ between these two regions is 30 years in men and 22 years in women. Alaska's $LE_0$ is the same as that of the United States, but for Alaska Natives, there is a drop of about 5 years. In Canada, there is a difference of 8 years between Nunavut and all Canadians, and less of a gap in the other two territories. Greenland's $LE_0$ is about 10 years shorter than that of Denmark's. In Fennoscandia, there is little difference between the northern and the national $LE_0$. Russia is suffering an unprecedented health crisis: the male $LE_0$ is about 60 years nationally, while some northern regions are even worse off.

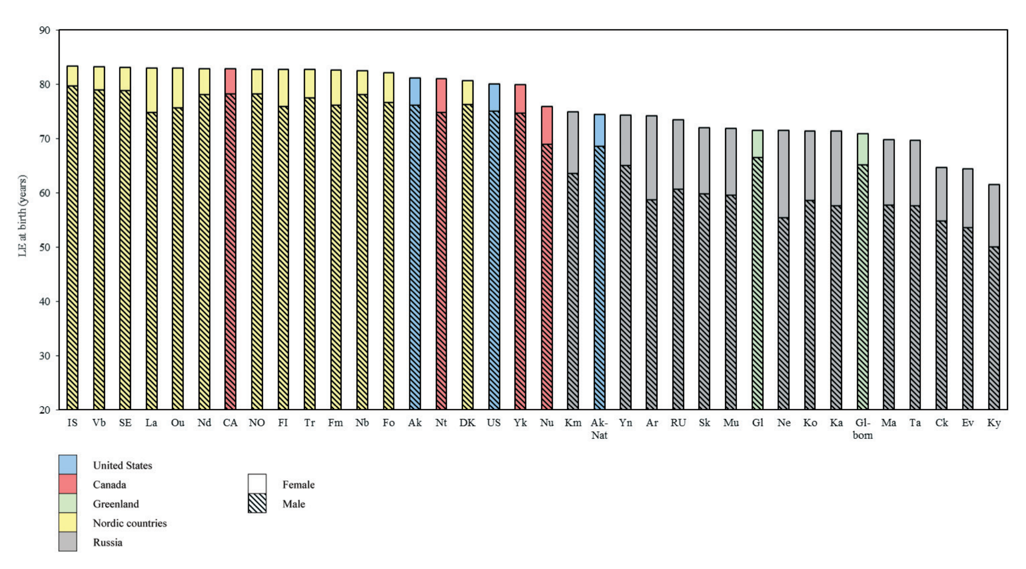

**Figure 1.7.** Life expectancy at birth in circumpolar countries and regions. Source: CircHOB.

### 1.8 Health Care Expenditures

A major challenge in an international comparison of health care expenditures is to ensure that what is counted as health care is the same entity in different countries, with different health care systems and financial management practices. The OECD developed the *System of Health Accounts* (SHA) to facilitate international comparisons, although its adoption is not universal and deviations exist among countries that have implemented it (OECD, Eurostat, & WHO 2011).

SHA distinguishes between health care (HC) and health care-related (HC.R) expenditures, with the following codes:

HC.1 Services of curative care
HC.2 Services of rehabilitative care
HC.3 Services of long-term nursing care
HC.4 Ancillary services to health care
HC.5 Medical goods dispensed to out-patients
HC.6 Prevention and public health services
HC.7 Health administration and health insurance
HC.9 Not specified
HC.R1 Capital formation of health care provider institutions

*HC.1 – HC.5 = total expenditures on personal health care*
*HC.6 – HC.7 = total expenditures on collective health care*
*HC.1 – HC.9 = total current expenditure*
*HC.1 – HC.9* and *HC.R1 = total health expenditures*

The following health-related expenditures are not included:

HC.R2 Education and training of health personnel
HC.R3 Research and development in health
HC.R4 Food, hygiene and drinking water control
HC.R5 Environmental health
HC.R6 Administration and provision of social services in kind to assist living with disease and impairment
HC.R7 Administration and provision of health-related cash-benefits

In addition there are parallel schemes for providers (HP) such as hospitals, ambulatory care providers, nursing and residential care facilities, etc, and sources of financing (HF).

Public expenditure on health care refers to health expenditure incurred by funds provided by national, regional and local government bodies and social security schemes. Privately funded sources of total health expenditure include out-of-pocket payments, private insurance programmes, charities and occupational health care paid for by the employers.

Health care expenditures based on OECD methodology are largely comparable across countries but not generally produced for regions within countries. The boundary between health care and social welfare services is difficult to delineate in some countries where the two are integrated. This is especially true of the care of the elderly.

Multiple national currencies can be converted into a single, comparable currency - the US dollar purchasing power parities (USD-PPP) - which recognizes the fact that the same amount of currency can buy more things in some countries than others. This permits a common standard against which to compare per capita health expenditures in circumpolar countries.

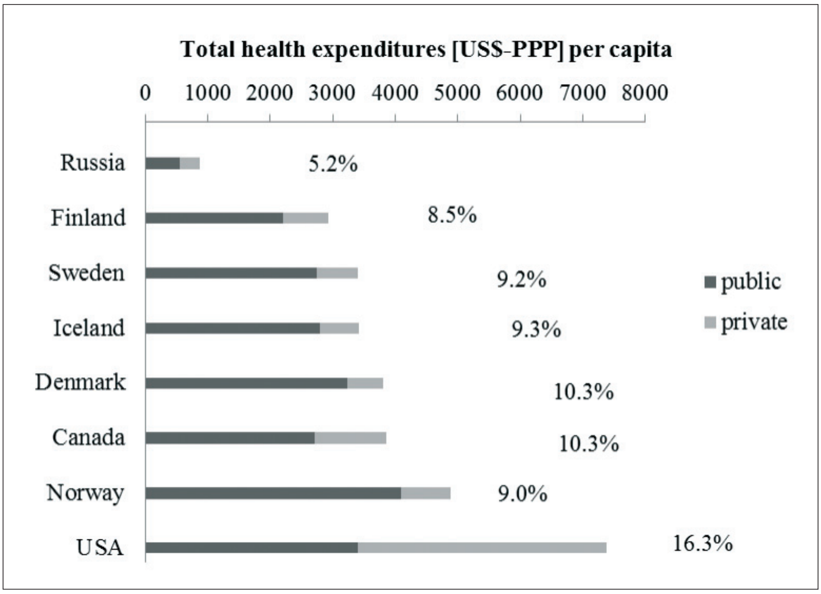

**Figure 1.8.** Per capita total health expenditures and per cent of GDP in circumpolar countries, 2005-09. Source: Russian data from WHO *National Health Accounts*; all other countries from *OECD Health Data 2011*.

The total per capita health expenditures in USD-PPP, health expenditures as a percentage of the gross domestic product, and the relative share of the private and public sectors across the 8 circumpolar countries over the 2005-09 period are shown in **Fig.1.8**.

For within-country comparisons (**Table 1.4**), the OECD method may not be consistently applied or used at all and only certain types of expenditures are available. The main purpose is to compare the northern regions with their respective countries as a whole on the same type of expenditures, and expressed in the national currency. This would allow one to assess the presence and extent of health care disparities. However, due to differences in definitions, it is not advisable to make comparisons across regions in different countries, even after currency conversion.

***United States***

United States national and state data (in US dollars) are available from the National Health Expenditures Accounts maintained by the Centers for Medicare and Medicaid Services (2011). Data on personal health care only (ie. HC.1 to HC.5) are available by state of residence, i.e. services provided to state residents anywhere in the United States.

***Canada***

Canadian national, provincial and territorial data (in Canadian dollars) are available from the Canadian Institute for Health Information's National Health Expenditure Database as reported in the annual *National Health Expenditure Trends* (CIHI, 2011a). It follows closely OECD methods.

**Table 1.4.** Comparison of selected per capita health expenditures in northern regions with "national" data.

| Country/Region | Currency | 2000-04 | Ratio | 2005-09 | Ratio |
|---|---|---|---|---|---|
| United States | USD | 4772 | 1.0 | 6291 | 1.0 |
| Alaska | USD | 5669 | 1.2 | 8110 | 1.3 |
| Canada | CAD | 3680 | 1.0 | 4893 | 1.0 |
| Yukon | CAD | 4858 | 1.3 | 7062 | 1.4 |
| Northwest Territories | CAD | 6042 | 1.6 | 8678 | 1.8 |
| Nunavut | CAD | 8136 | 2.2 | 11048 | 2.3 |
| Denmark | DKK | 22260 | 1.0 | 24933 | 1.0 |
| Greenland | DKK | 14724 | 0.6 | 17113 | 0.6 |
| Faroe Islands | DKK | 14886 | 0.8 | 18171 | 0.8 |
| Iceland | ISK | 278079 | 1.0 | 396268 | 1.0 |
| Norway | NOK | 14660 | 1.0 | 20068 | 1.0 |
| Nordland | NOK | 16714 | 1.1 | 24998 | 1.2 |
| Troms | NOK | 16689 | 1.1 | 24846 | 1.2 |
| Finnmark | NOK | 17152 | 1.2 | 25452 | 1.3 |
| Sweden | SEK | 14696 | 1.0 | 19759 | 1.0 |
| Västerbotten | SEK | 15298 | 1.0 | 20780 | 1.1 |
| Norrbotten | SEK | 16494 | 1.1 | 21774 | 1.1 |
| Finland | EUR | 1079 | 1.0 | 1426 | 1.0 |
| Pohjois-Suomi | EUR | 1086 | 1.0 | 1421 | 1.0 |
| Lappi | EUR | 1155 | 1.1 | 1553 | 1.1 |
| Russian Federation | RUB | 1656 | 1.0 | 4573 | 1.0 |
| Murmansk Oblast | RUB | 2174 | 1.3 | 6224 | 1.4 |
| Kareliya Republic | RUB | 2009 | 1.2 | 5146 | 1.1 |
| Arkhangelsk Oblast | RUB | 1756 | 1.1 | 4130 | 0.9 |
| - Nenets AO | RUB | 7162 | 4.3 | 21442 | 4.7 |
| Komi Republic | RUB | 2670 | 1.6 | 5249 | 1.1 |
| Yamalo-Nenets AO | RUB | 7199 | 4.3 | 16297 | 3.6 |
| Khanty-Mansi AO | RUB | 7863 | 4.7 | 19856 | 4.3 |
| Taymyr AO | RUB | 11175 | 6.7 | - | - |
| Evenki AO | RUB | 8354 | 5.0 | - | - |
| Sakha Republic | RUB | 4776 | 2.9 | 8595 | 1.9 |
| Magadan Oblast | RUB | 5131 | 3.1 | 12569 | 2.7 |
| Koryak AO | RUB | 7370 | 4.5 | 19672 | 4.3 |
| Chukotka AO | RUB | 14016 | 8.5 | 21978 | 4.8 |

Note: See text for source of data and the type of expenditures referred to. Greenland and Faroe Islands are compared with Denmark, but expenditures for these regions are not included in Denmark's.

### *Denmark, Greenland and Faroe Islands*

Data for Denmark and its two self-governing territories of Greenland and Faroe Islands (in Danish kroner) are available from NOMESCO's annual report *Health Statistics in the Nordic Countries* and also its Social and Health Indicators database (NOMESCO, 2011).

### *Iceland*

Data for Iceland are from NOMESCO (2010, 2011). There is no "north-south" comparison for Iceland.

### *Norway*

In Norway the delivery of primary health care and public health services is the responsibility of municipalities, whereas "specialized health services" (which include general and psychiatric hospitals, ambulances, substance abuse treatment, and patient transportation) are provided by regional health authorities. Data are available from Statistics Norway's Statbank and published tables. For municipal health services, net operating expenditures (in Norwegian kroner) in the three northernmost counties are compared to Norway as a whole. For specialized health services (all expenditures inclusive of depreciation), the three counties constitute a single northern health region. In **Table 1.4** the per capita specialized health services for the northern health region is added to the per capita municipal health services of each of the three counties.

***Sweden***
In Sweden, total health expenditures (in Swedish kronor) are available at the level of the county, which is responsible for primary care, specialized somatic and psychiatric care (ie. hospitals), dental and other services. Net costs for health care to the county councils are reported annually by the Swedish Association of Local Authorities and Regions (*Sveriges Kommuner och Landsting*) (SKL, 2011).

***Finland***
For Finland, the comparison (in euros) was for "net expenditures of the municipal health sector", available from SOTKAnet, the indicator bank of the National Institute for Health and Welfare (*Terveyden ja hyvinvoinnin laitos,* THL). It refers to health services provided by the municipality to its inhabitants or purchased from other municipalities, the central government or private providers. Net expenditures refer to operating costs less operating income (such as payment transfers).

***Russia***
For Russia, expenditures (in rubles) of the "consolidated budget for health care and physical education" by regions are available from the periodic publication *Health Care in Russia* (*Zdravookhranenie v Rossii*) by the state statistical agency Rosstat (various years). These budgets combine the regional government budgets with the federal budget attributable to the specific regions. Due to the dissolution of the Taymyr, Evenki and Koryak AOs, data for these regions are incomplete in the later period.

***Major patterns and trends***
Among circumpolar countries the United States and Russia are at two extremes – the former having the highest per capita health expenditures, accounting for 16% of GDP, whereas the per capita health expenditures in Russia was only one-tenth that of the United States, accounting for 5% of GDP. In the middle range are the Nordic countries and Canada, all having similar levels of per capita health expenditures, with health care accounting for 8-10% of GDP. In some countries, notably Canada and Russia, certain northern regions have per capita health expenditures that are several times the national average. In northern Fennoscandia the per capita expenditure is only marginally higher than the national average (with Finnmark about 50% higher), while Alaska is only 20% higher than the United States nationally. Greenland and the Faroe Islands both report a lower level of per capita health expenditure than Denmark, the only instance where "north" is less than "south".

### 1.9 Health Care Facilities

A variety of hospitals exist in circumpolar regions, from highly specialized ones such as the Alaska Native Medical Center in Anchorage, and university hospitals in Tromsø, Umeå, and Oulu, to small ones in remote towns of Greenland and Svalbard.

There are different categories of hospital beds, serving acute care, psychiatric care, rehabilitation, long-term care and palliative care, which may be located in general hospitals or specialized institutions. Acute care beds are further allocated to different medical and surgical specialties. In Finland, Iceland and Greenland, a number of beds are attached to health centres, some of which are used for the care of elderly people. In Finland such beds account for over half of all beds in the country.

According to OECD, "curative (acute) care

**Table 1.5.** Comparison of rate of hospital beds (per 100,000) in northern regions with "national" data.

| | 2000-04 | Ratio | 2005-09 | Ratio |
|---|---|---|---|---|
| United States | 285 | 1.00 | 268 | 1.00 |
| Alaska | 221 | 0.78 | 223 | 0.83 |
| Canada | 198 | 1.00 | 212 | 1.00 |
| Yukon | 187 | 0.94 | 148 | 0.70 |
| Northwest Territories | 580 | 2.92 | 394 | 1.86 |
| Nunavut | 114 | 0.57 | 111 | 0.52 |
| *Northern Canada* | *329* | *1.66* | *236* | *1.11* |
| Denmark | 333 | 1.00 | 264 | 1.00 |
| Greenland | 185 | 0.56 | 186 | 0.70 |
| Faroe Islands | 403 | 1.21 | 330 | 1.25 |
| Iceland | 282 | 1.00 | 248 | 1.00 |
| Norway | 371 | 1.00 | 338 | 1.00 |
| *Northern Norway* | *362* | *0.98* | *335* | *0.99* |
| Sweden | 263 | 1.00 | 234 | 1.00 |
| Västerbotten | 379 | 1.44 | 325 | 1.39 |
| Norrbotten | 302 | 1.15 | 241 | 1.03 |
| *Northern Sweden* | *341* | *1.29* | *284* | *1.21* |
| Finland | 233 | 1.00 | 205 | 1.00 |
| Oulu | 253 | 1.08 | 200 | 0.98 |
| Lappi | 252 | 1.08 | 247 | 1.21 |
| *Northern Finland* | *252* | *1.08* | *214* | *1.04* |
| Russian Federation | 1017 | 1.00 | 899 | 1.00 |
| Murmansk Oblast | 1028 | 1.01 | 942 | 1.05 |
| Kareliya Republic | 1043 | 1.03 | 897 | 1.00 |
| Arkhangelsk Oblast | 1137 | 1.12 | 973 | 1.08 |
| - Nenets AO | 1378 | 1.35 | 1351 | 1.50 |
| Komi Republic | 1052 | 1.03 | 1009 | 1.12 |
| Yamalo-Nenets AO | 1112 | 1.09 | 960 | 1.07 |
| Khanty-Mansi AO | 964 | 0.95 | 848 | 0.94 |
| Taymyr AO | 2342 | 2.30 | 1781 | 1.98 |
| Evenki AO | 2673 | 2.63 | 2615 | 2.91 |
| Sakha Republic | 1373 | 1.35 | 1209 | 1.34 |
| Magadan Oblast | 1488 | 1.46 | 1355 | 1.51 |
| Koryak AO | 3387 | 3.33 | 2530 | 2.81 |
| Chukotka AO | 2264 | 2.22 | 2111 | 2.35 |
| *Northern Russia* | *1133* | *1.11* | *991* | *1.10* |

Note: See text for data sources.

beds" include beds in general and specialty hospitals, but exclude beds for other functions (such as psychiatry, rehabilitation, long-term and palliative care) in such hospitals, and all beds in mental health/substance abuse institutions. Within the Nordic countries, the term "somatic" care or beds is used, which corresponds to general acute care hospitals elsewhere, and excludes psychiatric and long-term care beds. **Table 1.5** provides internal comparison within circumpolar countries, between their northern regions and the national average (or in the case of Greenland and Faroe Islands, with Denmark). The data sources and type of hospital beds being compared are discussed under each country. **Table 1.5** should not be used to compare countries.

***United States***

Data for hospital beds in community or short-term general hospitals are obtained from *Hospital Statistics,* produced annually by the American Hospital Association (AHA, various years).

***Canada***

Hospital beds data are available from the Canadian Institute of Health Information's Canadian Management Information System Database (CMDB). Data for "general hospitals" for two fiscal years (2004/05 and 2008/09) are shown (special request from CIHI).

***Denmark, Greenland and Faroe Islands***

Data on the total number of medicine and surgery beds, excluding psychiatry and long-term care, are obtained from NOMESCO (2010, 2011).

***Iceland***

Iceland does not report hospital bed data to either OECD or NOMESCO. However, the total number of bed-days per year is published by the Directorate of Health (*Landlæknisembættið*). By dividing this by 365, the total number of beds can be determined, assuming full occupancy.

***Norway***

Hospital beds refer to "general hospitals and other institutions" (*somatiske institusjoner*). Private hospital beds are included. Data for the whole of Norway and the northern health region are from Statistics Norway's StatBank.

**Sweden**

Data on specialized somatic care beds are available from the *Statistical Yearbook* (Statistics Sweden, various years). These beds are operated by county and municipal governments, with a small number of private hospital beds.

***Finland***

Hospital beds data were from SOTKAnet of the National Institute for Health and Welfare (THL), and refer to "specialized somatic health care", including hospitals in both the private and public sector, but excluding psychiatric beds and nursing-home type beds operated by municipal health centres. The number of beds reported are actually calculated by dividing the total number of bed-days by 365.

***Russia***

Data are reported in *Health Care in Russia* (Rosstat, various years). The number of beds in psychiatric and "narcological" hospitals are subtracted from the total number of beds. Data are only available for 2000, 2004, 2006 and 2008.

***Major patterns and trends***

A declining trend is evident in all countries and regions between the first and second half of the decade 2000-2009. Recognizing the limitation in cross-country comparisons, Russian regions tend to have higher rate of hospital beds than other circumpolar regions. There is no clear pattern in north-south disparities, with some northern regions having higher rates than the national norm and others having lower rates.

### 1.10 Health Care Personnel

The health workforce is made up of many different types of workers. WHO's International Classification of Health Workers recognizes five broad categories: (1) health professionals, (2) health associate professionals, (3) personal care workers in health services, (4) health management and support personnel, and (5) other health service providers (WHO, 2011). Health professionals vary substantially in their training, licensure and registration requirements, and how statistics on employment are collected. **Table 1.6** lists the names of selected health professionals in the languages of circumpolar countries.

Since 2010 OECD has made the distinction among the categories of "practising", "professionally active", and "licensed to practise". This is summarized in **Fig.1.9**. In earlier editions of *Health Data*, the distinction was made only between "practising" and "licensed to practise". Some countries are not able to distinguish "practising" and "professionally active" – these are either left blank, or they provide identical numbers for the two categories. There are usually two ways to count workers, by a head count or the number of full-time equivalent (FTE). It is difficult to apply the FTE concept to self-employed professionals who do not have regular hours of work. All data presented here are based on head counts. Some countries provide data from employment records, some conduct surveys, and some include only public sector employees.

Physicians and dentists include interns/residents, defined as trainees who have graduated but are undergoing further clinical training under supervision. Both generalists

**Table 1.6.** Names of selected health professionals in national languages of circumpolar countries.

| Canada | Denmark | Finland | Iceland | Norway | Sweden | USA | Russia |
|---|---|---|---|---|---|---|---|
| physician/ médecin | læge | lääkäri | læknir | lege | läkare | physician | vrach |
| dentist/ dentiste | tandlæge | hammaslääkäri | tannlæknir | tannlege | tandläkare | dentist | stomatolog |
| nurse/ infirmière | sygeplejerske | sairaanhoitaja | hjúkrunarfræðingur | sykepleier | sjuksköterska | nurse | medicinskaya sestra |
| public health nurse/ infirmière de santé publique | sundhed-splejerske | terveyden-hoitaja | heilsugæsluhjú-krunarfræðingur | helsesøster | distrikts-sköterska | public health nurse | |
| midwife/ sage-femme | jordemoder | kätilö | ljósmóðir | jordmor | barnmorska | midwife | akusherka |

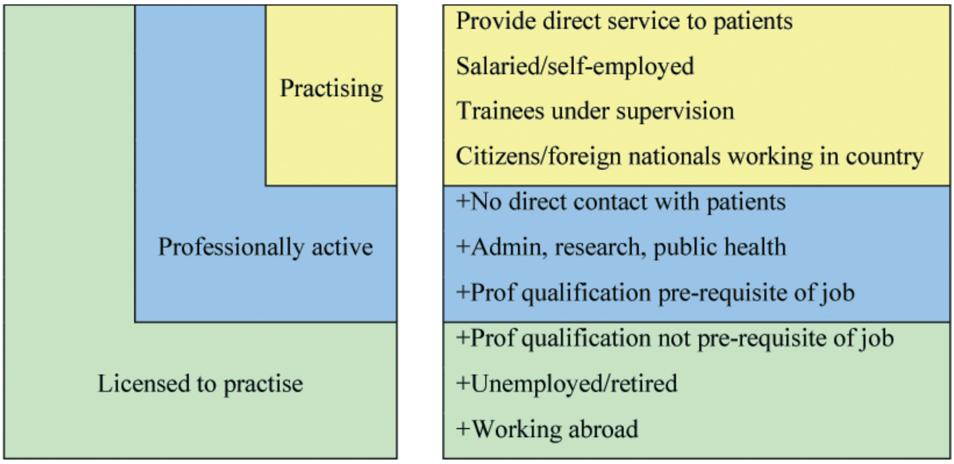

**Figure 1.9.** OECD classification of health professionals.

and specialists are included. The United States is unique in including doctors of osteopathy (DO) as physicians. DOs are now indistinguishable in training and scope of practice as doctors of medicine (MDs), whereas outside the U.S. osteopathy tends to treat only musculoskeletal disorders. Dentists (or stomatologists) are not included under physicians, although in some countries (such as Russia) they are considered medical specialists.

OECD includes under "professional nurses" clinical nurses, district nurses, nurse-anesthetists, nurse-educators, nurse-practitioners, public health nurses, and specialist nurses. Excluded are midwives (unless they are also registered as nurses and working as nurses), nursing aides, associate professional nurses, practical and vocational nurses.

**Table 1.7** to **Table 2.0** compare the rate of physicians, dentists, and professional nurses in circumpolar countries and regions. Where available, the data for "practising" professionals are shown, except where they are not distinguishable from the "professionally active".

***United States***

There is no single source of information on health human resources. For physicians, the source is the American Medical Association, accessible from the *Area Resource File* distributed by the Health Resources and Services Administration of the Department of Health and Human Services (HRSA, 2011). After 2003, both federal and non-federal physicians are counted. For dentists the source is the American Dental Association's publication *Distribution of Dentists* for 1998-2006, and annually thereafter (ADA, various years).

Data on nurses are obtained from the Occupational Employment Statistics database of the Bureau of Labor Statistics (2011). However, only salaried employees of health care institutions are included.

***Canada***

Data on physicians are from the Canadian Institute of Health Information's annual report on supply, distribution and migration of physicians (CIHI, various years); data on dentists from *Canada's Health Care Providers,*

**Table 1.7.** Rate of health professionals (per 100,000) in North America.

| | 2000-04 | Ratio | 2005-09 | Ratio |
|---|---|---|---|---|
| **Physicians** | | | | |
| United States | 279 | 1.00 | 288 | 1.00 |
| Alaska | 229 | 0.82 | 247 | 0.86 |
| Canada | 211 | 1.00 | 223 | 1.00 |
| Yukon | 171 | 0.81 | 218 | 0.98 |
| Northwest Territories | 107 | 0.51 | 107 | 0.48 |
| Nunavut | 29 | 0.14 | 37 | 0.16 |
| **Dentists** | | | | |
| United States | 59 | 1.00 | 60 | 1.00 |
| Alaska | 73 | 1.22 | 75 | 1.24 |
| Canada | 57 | 1.00 | 58 | 1.00 |
| Yukon | 73 | 1.28 | 106 | 1.82 |
| Northwest Territories | 62 | 1.08 | 114 | 1.96 |
| Nunavut | | | 142 | 2.45 |
| **Nurses** | | | | |
| United States | 779 | 1.00 | 821 | 1.00 |
| Alaska | 765 | 0.98 | 756 | 0.92 |
| Canada | 755 | 1.00 | 783 | 1.00 |
| Yukon | 908 | 1.20 | 1007 | 1.29 |
| Northwest Territories and Nunavut | 1101 | 1.46 | 1412 | 1.80 |

**Table 1.8.** Rate of health professionals (per 100,000) in Denmark, Greenland, Faroe Islands, and Iceland.

| | 2000-04 | Ratio | 2005-09 | Ratio |
|---|---|---|---|---|
| **Physicians** | | | | |
| Denmark | 298 | 1.00 | 334 | 1.00 |
| Greenland | 148 | 0.50 | 182 | 0.54 |
| Faroe Islands | 192 | 0.64 | 188 | 0.56 |
| Iceland | 354 | 1.00 | 362 | 1.00 |
| **Dentists** | | | | |
| Denmark | 85 | 1.00 | 85 | 1.00 |
| Greenland | 53 | 0.63 | 49 | 0.58 |
| Faroe Islands | 82 | 0.96 | 83 | 0.97 |
| Iceland | 100 | 1.00 | 94 | 1.00 |
| **Nurses** | | | | |
| Denmark | 963 | 1.00 | 972 | 1.00 |
| Greenland | 635 | 0.66 | 515 | 0.53 |
| Faroe Islands | 471 | 0.49 | 569 | 0.59 |
| Iceland | 829 | 1.00 | 855 | 1.00 |

*2000-2009 Reference Guide* (CIHI, 2011b); and data on nurses from *Regulated Nurse: Canadian Trends, 2005 to 2009* (CIHI, 2010). For physicians, interns and residents data are included under Canada but not in the North. NWT and Nunavut data are combined for nurses, and for dentists prior to 2004. Only Registered Nurses (RN) employed in nursing (including administration and research) are included. Although CIHI includes separate data for nurse-practitioners, these individuals are also registered nurses.

Note that the CIHI data refer to professionals registered to practise in the territories, who may be engaged in part-time practice consisting of multiple short visits, which may explain the high rate of supply (especially for dentists).

### *Denmark, Greenland, Faroe Islands, and Iceland*

Comparative data are from NOMESCO's *Health Statistics in the Nordic Countries* and the Social and Health Indicators database (NOMESCO, 2011).

### *Norway, Sweden, and Finland*

Data are from Statistics Norway's Statbank tables on labour force participations. Health professionals refer to "persons aged 16-66 with health care education employed in region". Nurses include public health nurses. Regional data for 2008 and 2009 refer to individuals aged 15-74; all other years refer to individuals aged 16-66. National data refer to individuals aged 16-66 for all years.

**Table 1.9.** Rate of health professionals (per 100,000) in Norway, Sweden and Finland.

| | 2000-04 | Ratio | 2005-09 | Ratio |
|---|---|---|---|---|
| **Physicians** | | | | |
| Norway | 359 | 1.00 | 418 | 1.00 |
| Nordland | 309 | 0.86 | 373 | 0.89 |
| Troms | 539 | 1.50 | 656 | 1.57 |
| Finnmark | 336 | 0.94 | 390 | 0.93 |
| Sweden | 327 | 1.00 | 366 | 1.00 |
| Västerbotten | 403 | 1.23 | 447 | 1.22 |
| Norrbotten | 242 | 0.74 | 268 | 0.73 |
| Finland | 217 | 1.00 | 226 | 1.00 |
| Oulu | 259 | 1.19 | 263 | 1.16 |
| Lappi | 158 | 0.73 | 181 | 0.80 |
| **Dentists** | | | | |
| Norway | 85 | 1.00 | 89 | 1.00 |
| Nordland | 70 | 0.82 | 82 | 0.92 |
| Troms | 89 | 1.05 | 98 | 1.10 |
| Finnmark | 71 | 0.84 | 76 | 0.85 |
| Sweden | 81 | 1.00 | 82 | 1.00 |
| Västerbotten | 91 | 1.12 | 85 | 1.04 |
| Norrbotten | 76 | 0.93 | 77 | 0.94 |
| Finland | 43 | 1.00 | 42 | 1.00 |
| Oulu | 53 | 1.23 | 55 | 1.31 |
| Lappi | 48 | 1.12 | 47 | 1.12 |
| **Nurses** | | | | |
| Norway | 1596 | 1.00 | 1821 | 1.00 |
| Nordland | 1502 | 0.94 | 1831 | 1.01 |
| Troms | 1875 | 1.17 | 2445 | 1.34 |
| Finnmark | 1495 | 0.94 | 1681 | 0.92 |
| Sweden | 1171 | 1.00 | 1240 | 1.00 |
| Västerbotten | 1458 | 1.24 | 1537 | 1.24 |
| Norrbotten | 1219 | 1.04 | 1292 | 1.04 |
| Finland | 671 | 1.00 | 757 | 1.00 |
| Oulu | 749 | 1.12 | 858 | 1.13 |
| Lappi | 669 | 1.00 | 781 | 1.03 |

**Table 1.10.** Rate of health professionals (per 100,000) in Russia.

| | 2000-04 | Ratio | 2005-09 | Ratio |
|---|---|---|---|---|
| **Physicians** | | | | |
| Russian Federation | 431 | 1.00 | 451 | 1.00 |
| Murmansk Oblast | 438 | 1.02 | 433 | 0.96 |
| Kareliya Republic | 477 | 1.11 | 477 | 1.06 |
| Arkhangelsk Oblast | 448 | 1.04 | 469 | 1.04 |
| - Nenets AO | 321 | 0.74 | 433 | 0.96 |
| Komi Republic | 383 | 0.89 | 417 | 0.92 |
| Yamalo-Nenets AO | 434 | 1.01 | 441 | 0.98 |
| Khanty-Mansi AO | 409 | 0.95 | 470 | 1.04 |
| Taymyr AO | 471 | 1.09 | 478 | 1.06 |
| Evenki AO | 527 | 1.22 | 549 | 1.22 |
| Sakha Republic | 449 | 1.04 | 508 | 1.13 |
| Magadan Oblast | 524 | 1.22 | 508 | 1.13 |
| Koryak AO | 543 | 1.26 | 393 | 0.87 |
| Chukotka AO | 584 | 1.35 | 723 | 1.60 |
| Northern Russia | 435 | 1.01 | 462 | 1.02 |
| **Dentists** | | | | |
| Russian Federation | 40 | 1.00 | 44 | 1.00 |
| Murmansk Oblast | 43 | 1.08 | 42 | 0.95 |
| Kareliya Republic | 17 | 0.42 | 14 | 0.33 |
| Arkhangelsk Oblast | 62 | 1.53 | 64 | 1.46 |
| - Nenets AO | 40 | 0.99 | 43 | 0.98 |
| Komi Republic | 42 | 1.04 | 41 | 0.95 |
| Yamalo-Nenets AO | 38 | 0.95 | 42 | 0.96 |
| Khanty-Mansi AO | 41 | 1.03 | 51 | 1.16 |
| Taymyr AO | 44 | 1.09 | 39 | 0.89 |
| Evenki AO | 34 | 0.84 | 35 | 0.80 |
| Sakha Republic | 28 | 0.70 | 34 | 0.79 |
| Magadan Oblast | 37 | 0.92 | 30 | 0.70 |
| Koryak AO | 46 | 1.14 | 44 | 1.00 |
| Chukotka AO | 60 | 1.49 | 74 | 1.69 |
| Northern Russia | | | | |
| **Nurses** | | | | |
| Russian Federation | 709 | 1.00 | 738 | 1.00 |
| Murmansk Oblast | 923 | 1.30 | 941 | 1.27 |
| Kareliya Republic | 829 | 1.17 | 836 | 1.13 |
| Arkhangelsk Oblast | 918 | 1.29 | 978 | 1.32 |
| Nenets AO | 590 | 0.83 | 695 | 0.94 |
| Komi Republic | 882 | 1.24 | 916 | 1.24 |
| Yamalo-Nenets AO | 883 | 1.24 | 901 | 1.22 |
| Khanty-Mansi AO | 890 | 1.26 | 1002 | 1.36 |
| Taymyr AO | 740 | 1.04 | 848 | 1.15 |
| Evenki AO | 423 | 0.60 | 613 | 0.83 |
| Sakha Republic | 862 | 1.22 | 892 | 1.21 |
| Magadan Oblast | 883 | 1.24 | 999 | 1.35 |
| Koryak AO | 882 | 1.24 | 878 | 1.19 |
| Chukotka AO | 846 | 1.19 | 1063 | 1.44 |

Swedish national and regional data are from the National Board of Health and Welfare (*Socialstyrelsen*) database. Nurses include public health nurses. Health care personnel data can only be found in the Swedish version of the website (Socialstyrelsen 2011).

Finnish data are from SOTKAnet which includes only public sector employees employed by municipal health services. Only dentists employed in primary care are included. Nurses include public health nurses (THL, 2011). Oulu data are obtained by combining the *maakunta* of Kainuu and Pohjois-Pohjanmaa.

***Russia***

Data are as reported in *Health Care in Russia* (Rosstat, various years). Included under "dentists" are stomatologists (*stomatologi*) but not middle-level dentists (*zubnye vrachi*). The number of stomatologists, however, is deducted from the total number of physicians. Nurses (*medicinskie sestry*) and midwives (*akusherki*) are middle-level health staff who, together with various health care technicians and assistants, are referred to as supporting medical personnel. No data are reported from Taymyr, Evenkia, Koryak AO after 2007.

***Major patterns and trends***

While Canada's northern territories and Greenland have far lower rates of physicians than Canada and Denmark, this is not the case in the Nordic countries or Russia, where some northern regions actually exceed the national norms. For nurses, the rate in the Canadian North is substantially higher than that for Canada nationally because of the nature of the system that is predominantly nurse-based, with nurses practising in an expanded role. For the other regions, there is no consistency in terms of a northern deficit or excess.

### CHAPTER 2.
### ALASKA

Kathryn Anderson

### 2.1 Introduction

Alaska is the only one of the 50 U.S. states that is considered Arctic. It is contiguous with Canada and non-contiguous with what is commonly referred to as the “Lower 48” states (**Fig.2.1**). Alaska is by far the largest state in the union, comprising some 1.5 million sq. km, which is approximately 20% of the landmass of the Lower 48 states. About one-third of Alaska lies above the Arctic Circle. Its southernmost city is Ketchikan, at 55°N, and its northernmost city is Barrow, at 72°N, a total distance of 2090 km by air.

The state of Alaska differs from the rest of the U.S. in several ways that are pertinent to health care. Two-thirds of the land area is referred to as “remote rural,” meaning it is unreachable by road or ferry, and encompasses roughly all areas north of Fairbanks and west of Anchorage. It contains only 9% of the state’s population. This is in sharp contrast to the “other” more urban and accessible Alaska (**Table 2.1**). The vast majority of the popula-

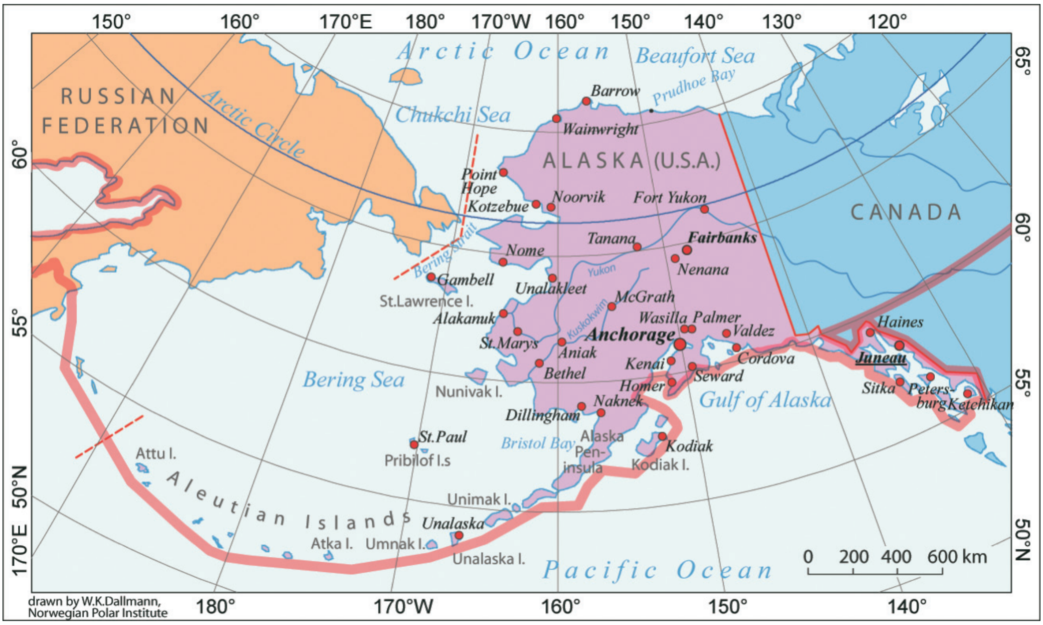

**Figure 2.1.** Map of Alaska and its population centres.

tion lives in or near the three largest cities: 54% in metropolitan Anchorage, 14% in Fairbanks, and 4% in Juneau. Compared to the United States as a whole, Alaska has a relatively low concentration of people over the age of 65 (7% vs. 13%), a relatively high concentration of indigenous population (15% vs. 1%) and a relatively high concentration of military personnel (14% vs. <1%).

**Table 2.1.** Key characteristics of Remot Rural Alaska and the Other Alaska.

| | Remote Rural Alaska | Other Alaska |
|---|---|---|
| Land area | 1,023,000 km$^2$ | 453,000 km$^2$ |
| Number of communities | 150 | 200 |
| Population size | 60,500 | 610,000 |
| Population density | 0.06 persons/km$^2$ | 1.3 persons/km$^2$ |
| Share of total population of state | 9% | 81% |
| Proportion of population that is Native | 78% | 12% |

Source: Goldsmith (2008).

The structure of health care in the United States reflects the nation's federal system which divides power between the 50 states and the national government. This structure in part explains both the complexity of health care and the volatility of health care reform activities. Either the United States Congress or the various state legislatures may issue laws regarding health care. If there is a conflict between a federal law and a state law, the federal law takes precedence. The landmark health care reform bill passed by the Obama administration in early 2010 is being contested in federal court on the basis of federal infringement of state rights, and its fate will be largely determined by the outcome of an expected 2012 Supreme Court case and the 2012 presidential election (Anderson, 2010). At issue is the so-called "individual mandate," requiring all individuals to obtain health care insurance.

States regulate physicians, hospitals, and insurance companies. At the federal level, most direct health care programs are administered by the Department of Health and Human Services (DHHS), headed by the Secretary of Health and Human Services, a member of Cabinet. DHHS's operating divisions include the Federal Drug Administration, the Centers for Disease Control and Prevention (CDC), the Centers for Medicare and Medicaid Services (CMMS), the Indian Health Service (IHS), Health Resources and Services Administration (HRSA), the National Institutes of Health (NIH), and the Agency for Health Care Research and Quality (AHRQ), among others. The Department of Labor also has a role in administering employer-provided health insurance legislation and regulations.

The federal government has a major influence on health care through its Medicare program and the rules that it sets for Medicaid programs administered by the states. While the government clearly does not have the power to set reimbursement rates for private practice providers servicing private clients, it does in fact set or influence rates for the 30% of the population covered by its health care programs for the elderly, the severely disabled, and the poor.

### 2.2 Organizational Structure and Financing

***The U.S. national health care system***

There is not a single coordinated national health care system in the United States, but rather a complex combination of public and private payers funding public and private providers.

Private health insurance, in particular employment-based insurance, lies at the core of the American system, and about 64% of the population is part of this extensive private system. The payers in the private system are health insurance companies, employers, and individuals. The providers in the private system are typically commercial or semi-commercial institutions (e.g. individual physicians, non-profit or for-profit hospitals and clinics). Some public facilities accept private insurance including rural health clinics or tribally operated hospitals in Alaska whose main source of funding is the federal government.

The two largest government payers are Medicare and Medicaid. Both of these systems require most patients to pay some portion of the service charge for an engagement, typically from 10% to 20%. American Indians and Alaska Natives whose only health coverage is the IHS are considered to be uninsured, according to the definition used by the Census Bureau's surveys on health insurance coverage (U.S. Census Bureau, 2011b). Indigenous people may be eligible for employer-based private insurance, Medicare, Medicaid, and/or military health services as well as those provided by the indigenous health system. Medicare, which is funded and administered by the federal government, covers those with severe disability and those who are age 65 and older. Medicare is funded primarily through employment taxes. Medicaid covers low-income individuals and families. Coverage eligibility is defined by the federal government but administered separately by each state. States may modify the terms of the program through a formal waiver process, and, within limits, define their own criteria for eligibility. Medicaid is funded by both state and federal taxes.

Outside of Medicare and Medicaid, government sponsored health care is typically paid for directly by the sponsoring agency rather than through insurance. In these cases, care is typically delivered through direct channels and employed health care providers rather than through referral to private providers. The military system for veterans, the military system for active duty service members, and Indian Health Service for indigenous peoples are all part of the public subsystem. Some municipalities provide direct care for the unemployed and indigent population.

***Alaska's health care systems***

While the structure of the Alaska health care system does not differ from the national system, there are differences in emphasis. Alaska's private/public/uncovered split is similar to the national average (**Fig.2.2**), but within the public system, Alaska has substantially more military coverage (13% vs. 4%) and substantially less Medicare coverage (9% vs. 14%). In Alaska 15% of the population is covered by IHS compared to 1% of the national population (U.S. Census Bureau, 2009).

The Alaska Tribal Health System is a voluntary organization of nearly 40 tribes and tribal organizations that provide health service to the indigenous population. Each of these organizations operates independently, but they work together to accomplish their mission of improving the health status of their constituents. The tribal system operates seven hospitals, 36 tribal health centres, 166 tribal Community Health Aide clinics, and five residential substance abuse treatment centres. In 2006, statewide, over $800 million was spent on health care for Alaska Natives. Medicare, Medicaid, and private insurance paid for

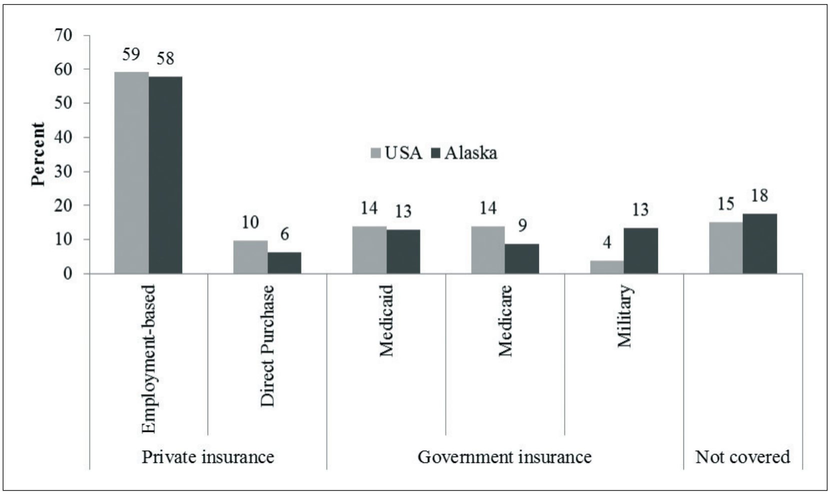

**Figure 2.2.** Percent of the population in Alaska and the United States with health insurance coverage, 2005-2009. Source: U.S. Census Bureau (2011c).
Note: totals > 100% because individuals may have more than one form of coverage.

an estimated 40%, while IHS covered 60% (Alaska Health Care Commission, 2009).

The Alaska Native Tribal Health Consortium (ANTHC) oversees tribal health service organizations in the state. ANTHC is owned by Alaska Native people and managed by all of the tribes. The Alaska tribal health system is a different arrangement than that experienced by American Indians in most of the Lower 48, where, in general, services are more directly managed by the Indian Health Service.

The military health care system has both direct-service and health insurance components. Active and retired military personnel and, in most cases, their families are eligible for direct-delivered services through military facilities by military personnel. In addition, the military provides contracted care through selected civilian providers, for use when there is no access to or capacity in the military direct system. The Veterans Health Administration system is separate from the active military system, although some facilities and services are shared.

***Health care financing***

The United States spent almost $2.5 trillion in healthcare in 2009 and Alaska spent $7.2 billion. Per capita spending over the period 2005-09 was $6291 in the U.S. and $8110 in Alaska, a premium of almost 30%. This cost difference varies by service. A report by the Institute of Social and Economic Research at the University of Alaska Anchorage cites hospital costs to be 42% higher in 2003, while the overall costs paid by private insurers for medical/surgical procedures were 18% higher, and the average costs of a visit to a doctor's office were 30% higher than the national average in 2001 (Foster & Goldsmith, 2006).

The major categories of U.S. health care spending, as reported in the annual National Health Expenditures Account, includes personal health care and administration.

Personal health includes hospital care, physician and clinical care, home health care, rehabilitative and long term care, and prescription drugs. Administration includes government administrative costs, public health, and research. It also includes the "net cost of private health insurance," which refers to insurance company administrative overhead and profit.

**Table 2.2** compares health expenditures by type of service in the U.S. and Alaska for the 2000-04 and 2005-09 periods. Only data on personal health services are provided by CMMS at the level of the state of residence. Alaska spends less than the nation on home care and nursing home care, likely due to its relatively smaller elderly population.

In the United States as a whole, about 32% of all national health expenditures were paid by private health insurance, 12% by individuals directly, and the remaining 66% by government (including Medicare, Medicaid, the military, and the Indian Health Service) (CMMS 2011). In Alaska, they were 50%, 9%, and 42%, respectively (M. Foster, Institute of Social and Economic Research, University of Alaska Anchorage, personal communication).

### 2.3 Delivery of Health Services

### *Primary care*

Primary care in the U.S. includes health promotion, disease prevention, counseling, patient education, diagnosis and treatment of acute and chronic illnesses. Primary care is usually performed by a physician or a mid-level practitioner such as a nurse practitioner (NP) or a physician assistant (PA). NPs and PAs refer patients to other health professionals such as surgeons, medical specialists, dietitians, and physical therapists when appropriate.

Outside of the three major cities, much of primary care in Alaska is delivered through federally designated Community Health Clinics (CHCs). CHCs are found throughout rural America. In Alaska, some of these clinics are tribally owned and managed, and some are separate non-profit organizations. There are a few such clinics in urban Alaska, as well, to address the needs of low-income and uninsured populations. Funding comes in the form of federal grants, which require the clinics to provide primary care, laboratory and radiology services, preventive service,

**Table 2.2.** Per capita personal health care expenditures in Alaska and the United States.

| | 2000-04 | | | | | 2005-2009 | | | | |
|---|---|---|---|---|---|---|---|---|---|---|
| | USA | % | Alaska | % | AK/US ratio | USA | % | Alaska | % | AK/US ratio |
| **Personal Health Care** | **4772** | **100** | **5669** | **100** | **1.2** | **6291** | **100** | **8110** | **100** | **1.3** |
| Hospital Care | 1697 | 36 | 2271 | 40 | 1.3 | 2271 | 36 | 3380 | 42 | 1.5 |
| Physician and Clinical Services | 1187 | 25 | 1600 | 28 | 1.3 | 1537 | 24 | 2302 | 28 | 1.5 |
| Other Professional Services | 152 | 3 | 208 | 4 | 1.4 | 198 | 3 | 294 | 4 | 1.5 |
| Dental Services | 251 | 5 | 337 | 6 | 1.3 | 318 | 5 | 434 | 5 | 1.4 |
| Home Health Care | 130 | 3 | 46 | 1 | 0.4 | 192 | 3 | 85 | 1 | 0.4 |
| Drugs and Other Medical Nondurables | 661 | 14 | 544 | 10 | 0.8 | 890 | 14 | 740 | 9 | 0.8 |
| Durable Medical Products | 93 | 2 | 88 | 2 | 0.9 | 111 | 2 | 102 | 1 | 0.9 |
| Nursing Home Care | 331 | 7 | 140 | 2 | 0.4 | 415 | 7 | 160 | 2 | 0.4 |
| Other Personal Health Care | 270 | 6 | 434 | 8 | 1.6 | 360 | 6 | 613 | 8 | 1.7 |

Source: Centers for Medicare and Medicaid Services (2011).

emergency medical services, and pharmaceutical services. The CHCs are expected to refer patients needing further medical, dental, substance abuse, or mental health services to the appropriate providers. CHCs have a strong focus on chronic disease prevention and management.

Because most villages in remote rural Alaska cannot support the cost of a full time primary care provider or a CHC, and because there is a shortage of medical providers willing to live in remote areas, much of the front-line care is provided by Community Health Aides (CHAs). CHAs are paraprofessionals, trained in basic clinical skills and health education. They are nominated by their home villages to be trained and certified through the Alaska tribal health system (Alaska Native Tribal Health Consortium, 2011). The CHA program is unique to Alaska.

The CHA model grew out of lay health-worker programs established in the 1950s to address tuberculosis epidemics in the remote rural villages. It has been extremely successful and its design is being re-used to develop dental health, behavioural health, and elder care aide programs. In 2007 there were 411 CHAs employed in Alaska, compared to 450 nurse-practitioners, 284 physician assistants, and 732 primary care physicians (Alaska Department of Health and Social Services, 2007).

Telehealth is in common use in the remote areas of the state, including otolaryngology, audiology, dermatology, cardiology, radiology, and behavioral health applications. These applications help to ameliorate both the lack of access to specialized care in remote areas and can help reduce the cost of medical transportation. At least two consortia have begun to address issues surrounding electronic health record (EHR) implementation on a state-wide level, but there is currently no guaranteed consistency of EHR format or application.

### *Hospital services*

There are 27 hospitals in Alaska, providing a total of 1723 beds (Alaska Health Care Commission, 2010), approximately 25 non-specialty beds per 10,000 residents. Acute care hospitals provide a wide range of surgical, medical, nursing, pharmaceutical, radiological, laboratory, and emergency services. Critical access hospitals, as defined by Medicare, provide a limited set of inpatient services. Critical access hospitals typically house a patient for less than 96 hours, at which time

**Table 2.3.** Number of hospitals and hospital beds in Alaska by type and ownership.

| | Private for profit | Private non-profit | Tribal | Military | State/ municipal | Total |
|---|---|---|---|---|---|---|
| Number of institutions | | | | | | |
| Acute care | 1 | 3 | 2 | 2 | 2 | 10 |
| Critical access | | | 5 | | 9 | 14 |
| Specialty | 1 | 1 | | | 1 | 3 |
| Total | 2 | 4 | 7 | 2 | 12 | 27 |
| Number of beds | | | | | | |
| Acute care | 250 | 552 | 200 | 160 | 120 | 1282 |
| Critical access | | | 93 | | 134 | 227 |
| Specialty | 74 | 60 | | | 80 | 214 |
| Total | 324 | 612 | 293 | 160 | 334 | 1723 |

Source: Alaska Health Care Commission (2010).

they are either discharged or transferred to an acute care hospital. Several critical access hospitals in Alaska are located off the road system, and patient transfer must be accomplished by air or boat. The regularity of harsh weather means that the transfer might not be timely. There are three specialty hospitals in Anchorage, two psychiatric and one extended care. The types of hospitals and their ownership are listed in **Table 2.3**.

The five largest acute care hospitals are in the three largest cities. Three are in Anchorage, including one not-for-profit hospital, one for-profit hospital, and the Alaska Native Medical Center, which is owned and operated jointly by two tribal health organizations. The fourth large acute care hospital is in Fairbanks and the fifth in Juneau, both owned by the local government. These are full service hospitals that provide care comparable to that available elsewhere in the United States. Some highly specialized care, including organ transplants, treatment for rare cancers, and severe trauma must be provided out-of-state. Seattle, in the state of Washington, is a 90-minute flight from the southernmost part of the state and a four-hour flight from Anchorage and is a common destination for this specialty care.

The military facilities are in Anchorage and Fairbanks, where most of the armed services are stationed.

***Public health***

The Division of Public Health in the State of Alaska Department of Health and Social Service has a budget of $84 million in 2009. Its major strategic initiatives include preventing and controlling epidemics, reducing death and disability due to injuries, preventing and controlling chronic disease and disabilities, ensuring emergency preparedness, assuring access to preventive services and quality health care, and protecting against environmental hazards

In addition to conducting surveillance and developing and implementing health interventions, DPH provides some direct services, such as screening and immunizations, through its workforce of about 130 public health nurses deployed statewide. Public health nurses function as a public health "safety net," meant to complement other sources of health care delivery. Tribal agencies also perform public health functions for their constituents, and state and tribal agencies work together to deliver coordinated services.

### 2.4 Patient Pathways

For indigenous people in remote rural Alaska, emergency care and basic primary care is provided by the Community Health Aides described above. They operate within an established referral hierarchy (**Fig.2.3**), which includes tribal mid-level providers, physicians, regional hospitals, and, ultimately, the main Alaska Native hospital in Anchorage.

Non-indigenous people living in remote rural areas have historically accessed tribal clinics for emergency care but not for preventive or maintenance care. However, with new federal funding arrangements under the Community Health Clinic umbrella, many remote clinics may now treat non-Native people for routine care. For most non-Native Alaskans, pathways to care usually bypass tribally-sponsored intermediate care providers. These patients are more likely to be referred directly to general private or public hospitals and physicians.

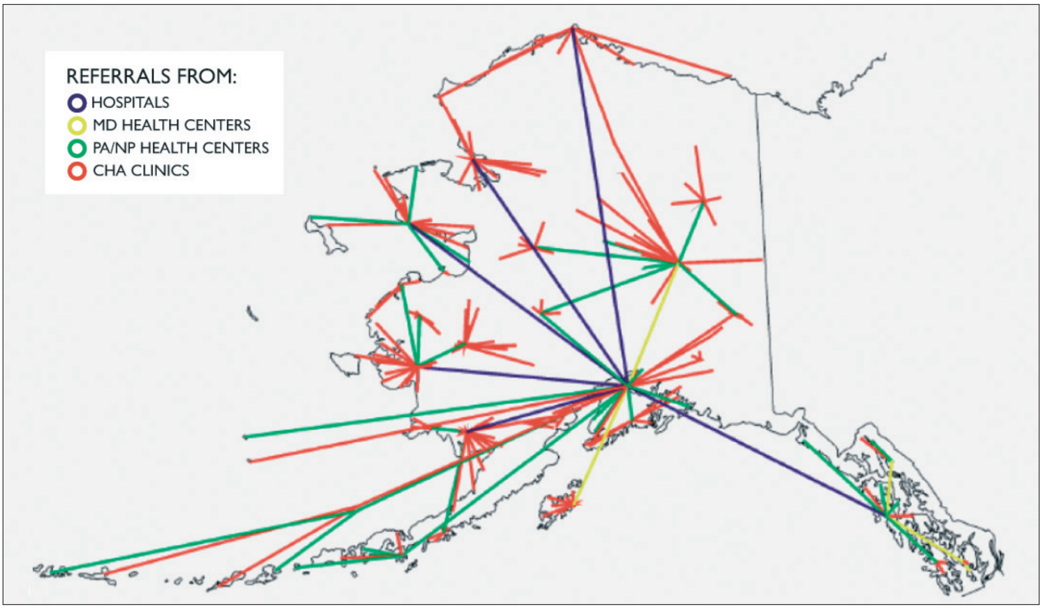

**Figure 2.3.** Referral patterns in the Alaska Native health care system. Source: Alaska Native Health Board (2011).

### 2.5 Human Resources Management

Health care is one of the largest private employment sectors in Alaska. With 30,300 employed in the Alaskan health system, this sector accounted for 9% of the labour force in 2009 (Fried, 2010). It is the fastest growing employment segment in the state, having grown 46% from 2000 to 2009, twice the growth rate of the health care workforce in the nation as a whole.

### *The health care workforce*

Key personnel include physicians, mid-level practitioners, nurses, nurses aides, community health aides, and dentists.

In order to reach the U.S. average density of physicians (23.8 per 10,000), Alaska, at 20.5 per 10,000, needs 200 more physicians, mostly in primary care (Alaska Department of Health and Social Services 2007). It is not clear if striving to reach this benchmark is appropriate, however, since Alaska's population, with its relatively low concentration of elderly people, may require fewer physicians per capita than the rest of the nation. On the other hand, the rate of physician assistants (75/100,000) and nurse-practitioners (102/100,000) in Alaska in 2010 was about 3 times and twice the rate respectively in the United States as a whole (Kaiser Family Foundation 2011).

The vacancy rates for various categories of health care workers ranged from 9% among pharmacists, 10% among registered nurses, 11% among family physicians, 13% among physician assistants, to 17% among nursepractitioners. For all physicians, the rate in rural areas was 23%, compared to 7% in urban areas (Alaska Center for Rural Health, 2009).

Health Professional Shortage Area (HPSA) is a federal designation used to describe an area that needs additional health care professionals and suffers from disparities such as access to care, health status, and poverty. In 2007, there were 29 such designations for primary care in Alaska, 27 for mental health, and 24 for dental care. All Alaskan HPSAs are located in small population centres and remote areas of the state, with the urban centres having no shortages. Health organizations and educational institutions serving a HPSA can apply for federal grant money for programs designed to eliminate the shortages.

***Training programs***

The University of Alaska offers professional training for dental hygiene, laboratory technicians, radiology technicians, nursing assistants, and emergency services personnel. It also offers degree programs in health sciences, health care management, nursing and public health.

The capacity of the College of Nursing has more than doubled between 2000 and 2009, increasing the number of graduates from 71 to 187. The nursing shortage in Alaska in the early 2000s had been so critical that private industry (largely health care providers) provided almost $5 million in funding to supplement the $11 million derived from tuition and state funding to expand the program. The nursing program has evolved to have a large distance-learning component, allowing students from remote areas to complete most of their training without moving to Anchorage. Several outreach efforts have resulted in a higher proportion of Alaska Native nursing students.

Alaska does not have its own medical school, but the state university participates in a cooperative program with four other northwestern U.S. states (known as the WWAMI program). In 2009, the number of seats allotted for Alaskan students doubled from 10 to 20. These Alaskan students complete their first year of medical school in Anchorage, their second year in Seattle, and their third and fourth years in rotations which can be in any of the five partner states.

The Alaska Family Practice Residency is a three-year program that allows medical school graduates interested in primary care to practice general medicine across the state in both rural and urban settings. The program emphasizes rural issues and cultural competency, as well as providing training in medical, surgical, obstetric, pediatric, and emergency medicine. Over 70% of the graduates of the residency choose to remain and practice in Alaska.

Community Health Aide training is conducted by the Alaska Area Native Health Service. Four training sessions of three to four weeks are conducted in a hub city, and the aide-in-training works in his or her village clinic in between the sessions. CHAs are not licensed by the state, but are certified by a tribe-operated board upon completion of their program, an exam, and a practicum.

***Recruitment and retention***

According to a study conducted by the Alaska Center for Rural Health (2006), over $24 million was spent in 2005-06 on recruiting physicians, pharmacists, mid-level practitioners, nurses, dentists, and others. Urban facilities accounted for $9 million of this spending, while rural facilities accounted for $15 million. Included in this spending was the cost of *locum tenens*, meaning itinerant workers hired from other states or countries to temporarily fill vacant positions. These costs include salaries, recruiting, training, and temporary housing costs.

The ACRH study discovered that the most effective recruiting methods for health professionals were word of mouth, the internet, and onsite visits. The top barriers to recruitment included salaries, geographic isolation/harsh living conditions, spousal compatibility/job availability, and lack of urban amenities. Suggestions for improvement of recruiting that arose in the course of the study include marketing to the proper candidates, who are more likely to have grown up in rural settings, enjoy outdoor sports, and seek professional autonomy. Other suggestions ranged from higher salaries to improved tort reform to enhance the quality and autonomy of professional life.

### 2.6 Conclusions

Alaskans can be covered by none, one, two, or more different health care systems, depending on their employment, ethnicity, military service, and socio-economic status. The federal government provides a large share of the care, through its programs for indigenous people, active and retired military personnel, the elderly, and those of lower socio-economic status. Private insurance, either through employers or purchased individually, pays for the care of those employees and customers so covered. In the case of private insurance, covered individuals share the costs through co-payments for service and/or insurance premiums.

In urban Alaska, care is similar to that found in the Lower 48 states. In rural Alaska, however, geographic and cost considerations lead to a different form of health care delivery, one which relies more heavily on paraprofessionals and mid-level practitioners and which, for more serious or extremely high-technology care, must provide transportation to larger facilities inside or outside Alaska.

Alaska has made progress towards "growing" its own health care workforce by investing in locally-based training programs. This has been important both because the need for health care workers is growing rapidly and because it is difficult to recruit and retain workers from more temperate and less isolated parts of the country.

### CHAPTER 3.
### NORTHERN CANADA

Gregory Marchildon and Susan Chatwood

### 3.1 Introduction

The Canadian federation is made up of ten provinces and three northern territories known as Nunavut, the Northwest Territories and Yukon, located north of 60°N and are often generalized as "northern Canada" (**Fig.3.1**). This review will be restricted to health care services in the three territories. Within this chapter, unique features related to territorial governance, population density, culture and service delivery models will be highlighted.

***Governance***

The territories are unique in that their legislative powers differ from Canada's provinces in two fundamental ways. First, unlike the provincial governments, territorial governments do not have the powers to amend their own constitution, as each territorial constitution is a federal act. Second, territories do not have control of the management and sale of public lands owned by the federal government. Negotiations are ongoing and each territory is unique in terms of powers which have been devolved from federal to territorial control. Similar to federal and provincial governments in Canada, the territorial governments largely function on the Westminster Parliamentary model. However, in a major break with this model, Nunavut and the Northwest Territories (NWT) rely upon a consensus style of non-party government that was deemed to be more culturally appropriate to the Aboriginal majorities living in those jurisdictions. As the only territory with a non-Aboriginal majority, Yukon conforms to the Westminster parliamentary system of competitive party government.

The federal government's control of health-care services was devolved to the departments of health and social services within the territorial governments in the late 1980s. Since that time, the territorial governments have administered their own health care services. It is of historical interest to point out that prior to the 1950s, governmental health care services were almost non-existent in northern Canada. Instead people depended on traditional knowledge and community designated indigenous healers and midwives to provide what we now call health services.

Through negotiations and devolution of federal powers, the territorial governments have assumed most of the social and economic responsibilities of provincial governments such as public health care, and are members of the federation's many intergovernmental forums including First Ministers' Conferences and the Council of the Federation. Nonetheless, all three territories have shallow tax bases, especially Nunavut, and depend heavily on federal transfer funding to exercise their full policy and program responsibilities.

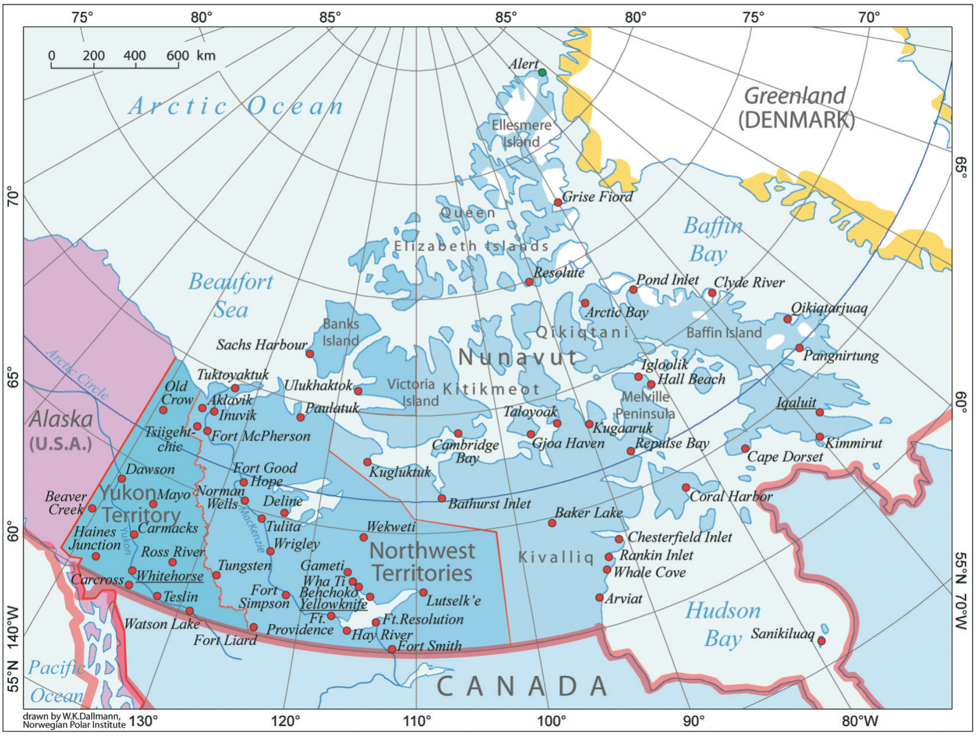

**Figure 3.1.** Map of the three northern territories of Canada and major population centres.

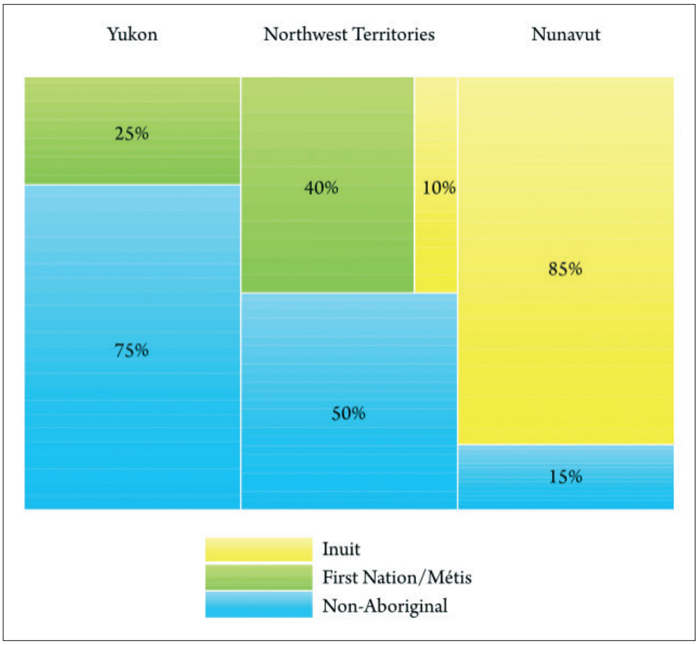

**Figure 3.2.** Distribution of Aboriginal groups in the Canadian North.

In addition to negotiations between federal and territorial governments regarding the devolution of federal powers, land claims in northern Canada have been settled between Aboriginal organizations and the federal government, which have in turn devolved powers to specific Aboriginal governments. In the case of Nunavut, the entire territory is under a single claim. Yukon has nine settled claims while the NWT has four settled claims with others still under negotiation. In most cases, health services have not been included in the negotiations and remain under territorial jurisdiction. However the option of devolving health services is possible and certain services have been devolved to Aboriginal authorities as part of indigenous land claims and treaty negotiations elsewhere in Canada.

***Population characteristics***

The population of Canada's three northern territories is little more than 100,000 people over an area that is almost 4 million km$^2$, one of the lowest population densities in the circumpolar world at 0.64 people per km$^2$ (**Table 1.2**). Just over half (52%) of the territories' total population is Aboriginal (**Fig.3.2**) in contrast to the provinces where less than 4% of residents identify themselves as Aboriginal (Statistics Canada, 2011). In relation to health services utilization it should be noted that Aboriginal peoples in the north experience health disparities which lead to a much higher proportion of Aboriginal peoples accessing health services (Marchildon, 2005).

Aboriginal groups in the three northern territories consist of First Nations, Inuit and Métis peoples . Within these groups there are a number of distinct groups. The distinctions of these groups are recognized through land claims as well as by the territorial governments through official language and traditional knowledge policies. Health services in the north are also shaped by governmental efforts to accommodate these distinctions through interpreter services, dietary programs, and Aboriginal specific services including access to traditional healers and community-based midwives. To date, this differential programming has had a limited impact on reducing gaps in health disparities between Aboriginal and non-Aboriginal Canadians (Frohlick et al., 2006).

### 3.2 Organizational Structure and Financing

Each territorial government has a department of health and social services that is responsible for administering a range of health and health care services, in particular medically necessary hospital and primary care services that are defined as "insured services" under the *Canada Health Act*, or "Medicare" as it is popularly known in Canada (Marchildon, 2005). The territorial ministers of health and social services (and the cabinets of which they form part) are accountable to territorial residents for access to, and the quality of Medicare through the territorial legislatures. They are also accountable to the federal government for meeting the five criteria of public administration, universality, accessibility, portability, and comprehensiveness under the *Canada Health Act*, and ensuring that such services are provided free to all residents at the point of service. In addition, these departments share the responsibility for administering public health services with the Government of Canada.

Health care is considerably more expensive on a per capita basis in northern Canada than it is in the country as a whole, with Nunavut ranking the highest, exceeding $11,000 over the 2005-09 period (CIHI, 2011a). Fig.3.3 compares the per capita total health expenditures and their distribution by type of function in Canada with the three northern territories.

Due to the fact that the federal government holds fiduciary responsibility for First Nations and Inuit peoples throughout Canada, the Government of Canada continues to fund and administer a number of health programs including a major coverage program that provides funding to eligible First Nations and Inuit specifically targeting non-Medicare services including: medical travel, dental care, pharmaceutical therapies and vision care, services that are sometimes covered in employment-based private health insurance plans (Health Canada, 2011). This non-insured health benefits (NIHB) program is administered by the territorial governments in the Northwest Territories and Nunavut, and by Health Canada in Yukon. As **Table 3.1** illustrates, the average contribution of NIHB is slightly more than $1,000 per NIHB beneficiary in the territories as a whole.

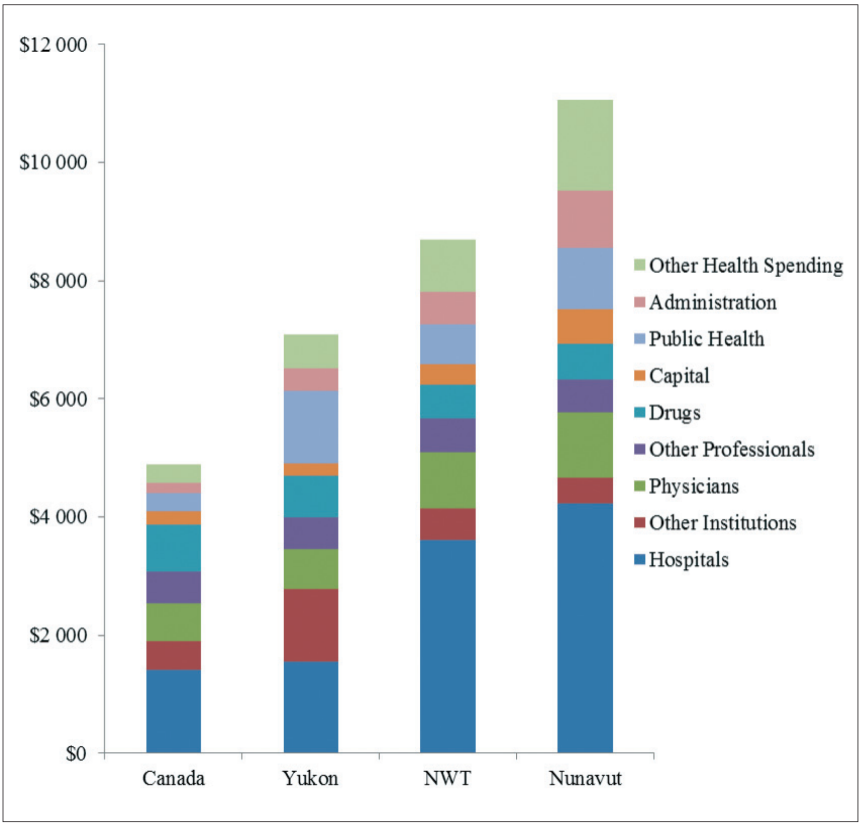

**Figure 3.3.** Distribution of per capita total health expenditures by use of funds in Canada and the three northern territories, 2005-09. Source: CIHI (2011a).

**Table 3.1.** Comparison of non-insured health benefits (NIHB) expenditures by category between northern and southern Canada, 2009-2010.

The federal government also funds a number of population health programs through the First Nations Inuit Health Branch of Health Canada and the Public Health Agency of Canada. These programs fall into four categories: (1) community care; (2) mental health and addiction; (3) children and youth programs; and (4) chronic disease and injury prevention. Funding is generally allocated via territorial or federal governments to Aboriginal and community-based organizations including local (Aboriginal and non-Aboriginal) governments in the territories.

Each territory has its own unique form of health administration. In the Northwest Territories, there are eight geographically-bounded regional health authorities (RHAs), governed by individual boards of directors, responsible for the administration and delivery of public health and primary care with various arrangements to ensure access to hospital care for all residents. The territorial hospital in Yellowknife is managed by a public administrator on behalf of the NWT government.

The Government of Yukon's Department of Health and Social Services is responsible for

**Table 3.1.** Comparison of non-insured health benefits (NIHB) expenditures by category between northern and southern Canada, 2009-2010.

| Type of expenditure | Northern Canada (3 Territories) | | Southern Canada (10 Provinces) | |
|---|---|---|---|---|
| | Total C$ millions | C$ per NIHB beneficiary | Total C$ millions | C$ per NIHB beneficiary |
| Medical Travel | 34.6 | 552 | 267.1 | 348 |
| Pharmacy | 20.6 | 328 | 391.1 | 509 |
| Dental | 19.6 | 313 | 171.1 | 228 |
| Vision Care | 3.3 | 52 | 24.5 | 32 |
| Other | - | - | 29.6 | 39 |
| Total | 78.1 | 1245 | 883.5 | 1150 |

Note: May not add up due to rounding. Results for per capita expenditures calculated by dividing expenditures in 2009-2010 by number of NIHB clients in March 2010. Source: Health Canada (2011).

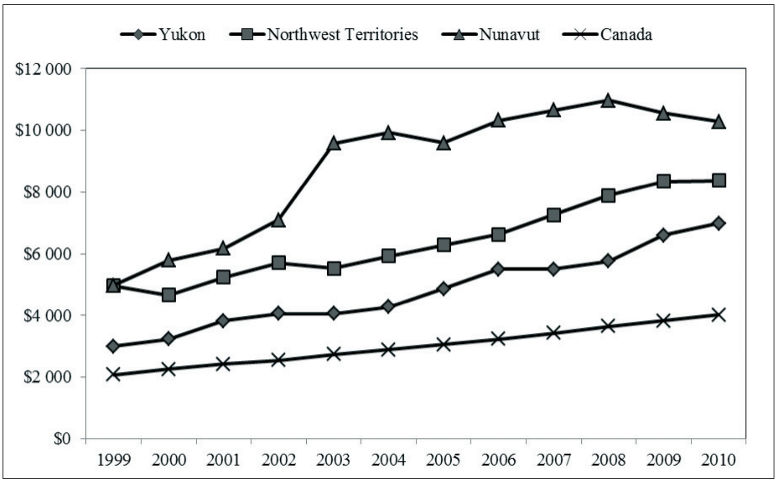

**Figure 3.4.** Trend in public sector per capita health expenditures in Canada and its three territories, 1999-2010. Source: CIHI (2011a).

administering and delivering all public health and medical services, with the exception of hospital services which are administered by public board – the Yukon Hospital Corporation. Some Yukon First Nations have agreements in place with the territorial government for the delegated delivery of specific community health services.

While there are no RHAs in Nunavut, the vast territory has been divided into three service regions with different administrative centres. In addition, the hospital in Iqaluit, the territory's capital city and most populous community (population 6,184 in 2006), is run directly by the Department of Health.

Similar to the provincial governments, territorial governments have seen their health care expenditures grow at a rate that exceeds inflation, economic growth, and other public service expenditures including education since the late 1990s (CIHI, 2011a). This has generated an impression that territorial health expenditures may be unsustainable in the long-term, and there have been recent initiatives to reduce cost and improve efficiency. As it is both difficult and expensive to provide comprehensive health care services in small, isolated communities scattered in such a vast geography, northern residents are transported vast distances – often by air – in order to access advanced diagnostic services, as well as specialized chronic, dental, maternal, medical, mental and emergent care services at regional and territorial centres.

Although private per capita health care expenditures in Yukon and the Northwest Territories track the Canadian average, those in Nunavut are considerably lower due to the lack of availability of such services. In all three territories, public sector spending exceeds 78% of the total spending compared to 70% in the provinces, while per capita public sector health expenditures are considerably higher than the Canadian average (CIHI, 2011a) (Fig.3.4).

### 3.3 Delivery of Health Services

In northern Canada, primary health care services and public health services are often delivered within collaborative models of care where linkages and overlap exist between the two (Young & Chatwood, 2009). In general, primary care services deal with the immediate responses to health concerns while public health activities tend to focus on the prevention of disease, injury and promotion of well-being.

***Primary care***

Primary care services are accessed in northern Canada through community based health clinics. In all territories the entry point for primary care services in remote communities is through nurses located in clinics administered by the territorial governments. The capital cities of the territories are serviced by family physicians who play a major role in the delivery of primary care. The territories vary considerably in terms of the proportion to general practitioners to general population – the proportion is considerably below the Canadian average in Nunavut and the NWT and well above the average in Yukon.

The remainder of the population, who primarily reside in remote communities, access nurses and in some cases midwives for primary care services. These primary care clinics – commonly known as community health centres – have been built on the nursing stations and outposts initially established by the federal government in the 1950's. Originally staffed by outpost nurses who tended to be midwives brought from overseas, the clinics today remain nurse-based. In all three territories, the community health centres are staffed by community health nurses (CHNs), the majority of whom are registered nurses (RNs), very occasionally supplemented by nurse practitioners (NPs) who have advanced practice standing. Delivering a broad range of health services in isolated conditions, RNs enjoy a scope of practice experience that goes beyond most RNs in southern Canada (Registered Nurses Association of the Northwest Territories and Nunavut, 2005). CHNs provide basic 24-hour, seven day a week emergency care, primary care services, as well as some public health services. Consultations with physicians, either in the regional centres, territorial capitals or in the south, are done through community visits by the physician or by telephone.

The number of CHNs in any given community health centre is a function of the community's population size and its overall health needs relative to surrounding communities. Where nurses used to practice in partnership with community health representatives in consultation with physicians, models with a team approach to care are now emerging. Teams provide a multidisciplinary approach that is more suited to managing lifestyle, complex diseases and cultural elements. Many innovations related to management of diabetes and maternal child care, to name just a few, are beginning to improve the quality of care and service satisfaction in the north (Health Council of Canada, 2009a and 2009b).

***Hospital services***

There are four hospitals in the territories that offer secondary and tertiary (more specialized) care. These hospitals have physicians available on call 24 hours a day and the ability to provide surgical services. This count does not include smaller regional hospitals that are really cottage hospitals such as those in Watson Lake (Yukon) or Cambridge Bay (Nunavut). Typically these smaller hospitals do not have continuous physician coverage nor do they offer any tertiary acute services. Each of these four territorial hospitals provides a reasonably broad range of secondary services, birthing services, emergency departments, day (ambulatory) surgery, physicians with specialties in obstetrics gynaecology, paediatrics, internal medicine, psychiatry, otolaryngology, and ophthalmology as well as health professionals providing advanced medical imaging, laboratory services, physiotherapy, occupational therapy and speech therapy. In addition, they offer some tertiary services via visiting specialists from the provinces and additional training and broader scope of practice for northern-based specialists. There are considerably fewer specialized services than those offered by a typical urban hospital in southern Canada. As a consequence, there are a large number of referrals and emergency medical evacuations to large urban hospitals in southern Canada in cities such as Vancouver, Edmonton, Winnipeg, Ottawa and Montreal. The referral patterns are a product of the agreements reached between the territorial departments of health and health authorities with hospitals, health regions and health ministries in the provinces.

The largest hospital in northern Canada is the Stanton Territorial Hospital located in

Yellowknife, Northwest Territories. With 83 inpatient beds and 30 ambulatory care (day) beds, Stanton Hospital also serves patients from the western half of Nunavut (Kitikmeot Region). There is also a 14 inpatient bed (the remaining 35 beds are emergency and ambulatory beds) facility in Inuvik, Northwest Territories. Located in the capital of Yukon, the Whitehorse General Hospital has 49 in-patient beds and ten surgical day beds. The Qikiqtani General Hospital, in Iqaliut, Nunavut, is a 35 in-patient bed facility.

***Public health***

Public health services can be divided into activities which are based at the territorial health departments under the jurisdiction of the territorial public health acts, and services overseen by the regional authorities. Programs within the government health departments are overseen by a chief medical/public health officer, and include activities such as disease surveillance, reportable diseases, food regulations and sanitation regulations. Some community-based public health services are delivered by nurses or community health representatives (CHRs) based in the community's main health centre. Community based public health programs include: cancer screening, immunizations, prenatal care, sexually transmitted infection clinics, well baby follow up and developmental screening. In addition, programs which primarily focus on reducing risk factor burden in areas such as smoking, obesity and substance abuse are carried out. As highlighted earlier the federal government funds Aboriginal groups and non-governmental organizations to implement these services including: the Community Care Program, Brighter Futures, Building Healthy Communities/Solvent Abuse Program, the National Native Alcohol and Drug Abuse Program, the Fetal Alcohol Spectrum Disorder Program, the National Aboriginal Youth Suicide Prevention Strategy, the Canadian Prenatal Nutrition Program, and the Aboriginal Diabetes Initiative and Injury Prevention Program.

As a consequence of broad mandate of territorial health ministers and their departments, there is some overlap, if not duplication, in the respective population health and public health policies and programs of the territorial and federal governments. Because of this, there is occasionally confusion and debate as to whether the federal government, the territorial government, or even a local organization or Aboriginal government, is responsible for population health and public health programming.

### 3.4 Patient Pathway

Residents in northern Canada access primary health care through community based health programs which are staffed by nurses with an expanded scope of practice in the smaller remote communities and general practitioners in the regional and territorial centres. The primary care health centres staffed by nurses provide drop-in services for individuals with primary care needs. A number of paediatric conditions as well as infectious diseases, chronic diseases, injuries and mental health conditions are managed at the health centres. Community health nurses in remote or smaller communities rely on telephone or telehealth consultation with a general practitioner, paediatrician, obstetrician or psychiatrist located in larger centres.

When necessary, patients are transferred by air to a general practitioner or specialist in a larger centre, either within the territories or in southern Canada.

More specialized diagnosis or treatment which require specialist physicians generally occur in the territorial capital or facility in southern Canada. Rehabilitation services, speech language, physiotherapy, occupational therapy and audiology are provided through travelling clinics and in clinics based at the regional hospitals.

A patient from a community with a medical condition will normally consult a nurse practitioner or registered nurse. The nurse will decide whether to treat the patient immediately or to consult with a physician at the regional health centre. Based on the condition of the patient, the physician will recommend local treatment, that the patient be seen by a doctor on the next visit to the community, or refer the patient to subacute or acute treatment at the regional or territorial health centre. Transfers can take place by scheduled flights or by specially arranged medical evacuation (medevac) flights. Once at the regional centre further referral to a tertiary hospital may take place as needed. Patients living in or visiting one of the regional centres will often consult the physician directly in the primary care clinic.

### 3.5 Emerging Technologies

In an effort to improve access to, and the quality of, healthcare in remote settings, each northern territory has its own telehealth program (Powers, 2011). The programs support the delivery of primary and secondary care services, home care, speech language, social services, administration, educational sessions and professional development. Challenges around utilization of the current telehealth systems often arise and applications in relation to the provision of care vary. Factors that contribute to poor utilization include cost, bandwidth limitations, staff turnover, clinicians' familiarity with the equipment, lack of access to equipment in some practice areas, and lack of dedicated protocols for use in practice and systems level applications. As technology advances, creative solutions that support service provision are now emerging: for example, using portable tablets for home care services in Yukon have demonstrated early success.

Electronic medical records (EMRs) are emerging as standard tools of practice and mechanisms to enhance delivery of care. The Northwest Territories' implementation of EMRs has taken an integrated health systems approach and now have 140 users on a single database which is routinely used from 12 different clinical locations, including 6 remote communities and the territorial hospital. The system is in the process of being fully integrated with the hospitalist, emergency room, obstetrics and anesthesia services as a communication tool, viewer, and episodic scheduler. Users include physicians, nurse practitioners, nurses, clinic assistants, home care, family counselling, public health, quality assurance, and administration. Picture archiving and communication systems (PACS) and electronic health records (EHR) have been rolled out to most of the 33 communities in the NWT. Nunavut is currently implementing an EHR that supports health care delivery across the continuum of care. A first wave of applications has been implemented in the larger centres and a roll out of patient cares systems, physician care manager and electronic medication administration records are expected over the coming years. Yukon's largest primary health care clinic in Whitehorse has been using an electronic medical records system since 1994. However, in order to achieve their full potential, EMRs, EHRs, PACS and video conferencing initiatives require integration and standardization within the existing territorial health systems (Powers, 2011).

### 3.6 Human Resources Management

Each of the northern territorial regions has community college systems which offer programs for community health representatives (CHRs), nurses and social workers. The nursing and social work programs based at the community colleges award university degrees through partnerships with provincial based universities. In Nunavut, a midwifery programs is offered at the territorial college and through community based programs.

While the territories aim to promote training for local and Aboriginal health personnel through strategic initiatives, they remain highly dependent on hiring health professionals from outside the territory. No medical schools exist in the northern territories although some provincial medical schools offer northern-based rotations for medical students and residents.

While Yukon has a rate of physicians comparable to that of Canada for the period 2005-09 (**Table 1.7**) at 220 doctors per 100,000 people, it was twice the NWT rate, and 6 times the Nunavut rate. According to the Canadian Community Health Survey in 2007, 78% of the population in Yukon reported having regular access to a medical doctor, while 41% and 13% reported the same in NWT and Nunavut respectively (CIHI, 2009). Due to the lower numbers of medical specialists in the territories, many general practitioners provide basic specialty services in addition to primary care thereby explaining to some extent the large number of physicians in Yukon (Stasyszyn, 2011).

**Table 3.2** highlights how nurses are distributed in northern Canada in relation to the rest of the country. Fewer nurses in the north have a single employer and many hold casual status. This is typical for agency nurses who are likely to also hold jobs outside the territory and only work part time in the territories. The northern territories report a higher proportion employed in community health agencies and less in hospitals. This is expected where nurses provide entry to primary care services in these community centres. Recruitment and retention in the remote communities remains a challenge. In Nunavut, where the nursing vacancy rate has been as high as 40%, there has been high reliance on agency nurses – nurses who are hired through companies and flown into the north for a contracted period of time at great expense to the territorial government.

Community health representatives (CHRs) are primarily Aboriginal and play an important role in health promotion and liaison between medical personnel, community stakeholders and patients. Each community health centre employs a CHR. In general the role of CHRs has been underdeveloped, despite their representing an innovative element in relation to providing culturally appropriate care within the northern health service delivery system (Hammond, 2006).

**Table 3.3.** The nursing profession in the three northern territories, 2009.

| Employed in Nursing | | Yukon Number | Yukon % | NWT and Nunavut Number | NWT and Nunavut % | Canada % |
|---|---|---|---|---|---|---|
| Employment status | Full time | 185 | 50.7 | 577 | 56.5 | 58.7 |
| | Part time | 103 | 28.2 | 0 | - | 30.6 |
| | Casual | 77 | 21.1 | 445 | 43.5 | 10.7 |
| | Unknown | 2 | - | 0 | - | - |
| Place of work | Hospital | 169 | 46.6 | 382 | 37.3 | 62.6 |
| | Community-based health agency | 128 | 35.3 | 420 | 41.5 | 14.2 |
| | Nursing home | 26 | 7.2 | 12 | 1.2 | 9.9 |
| | Other | 40 | 11.0 | 199 | 19.6 | 13.3 |
| Position | Managerial | 41 | 11.3 | 110 | 11.0 | 7.0 |
| | Staff/Community health nurse | 295 | 81.5 | 745 | 74.7 | 77.8 |
| | Other | 26 | 7.2 | 142 | 14.2 | 15.1 |
| Agency relationship | Single employer | 268 | 73.0 | 136 | 51.3 | 86.8 |
| | Multiple employer | 99 | 27.0 | 129 | 48.7 | 13.2 |

Source: CIHI (2010).

### 3.7 Conclusions

In northern Canada unique challenges in the delivery of appropriate services exist due to the widely dispersed populations, health human resource shortages, and the high proportion of indigenous populations who experience a disproportionate burden of disease. A number of adaptations have evolved in response to improve the scope and quality of services in this environment. Team approaches and indigenous-specific health programming have been introduced in order to prevent disease more effectively as well as provide treatment in an environment where cultural needs are addressed. Human resource challenges are being addressed through historically broad scopes of practice for nurses in remote settings, recruitment and retention strategies for all health care providers, and the promotion of health careers and access to northern- based college programs and southern universities.

Small population densities, isolated communities and the need for air travel in much of the Canadian north go some distance in explaining the high cost of health delivery. At the same time, however, the territorial health systems are extremely high cost, and in the case of Nunavut, the highest cost in the circumpolar world. This presents an enormous challenge to the territorial governments as well as the federal government, both of which fund the vast majority of services and are therefore responsible for containing costs and ensuring a more efficient use of fiscal and human resources.

There have been many efforts to deploy health information technology to address the challenges posed by geography and human resource allocations. There remain much untapped potential for systems enhancements through use of these tools that have the potential to improve clinical support, quality of care and communications in remote regions. The results from these initiatives in terms of achieving their original objectives have been mixed. Relative to the rest of Canada, health systems in northern Canada continue to struggle with health human resource shortages as well as the lack of northerners, particularly indigenous peoples, in key managerial and clinical positions.

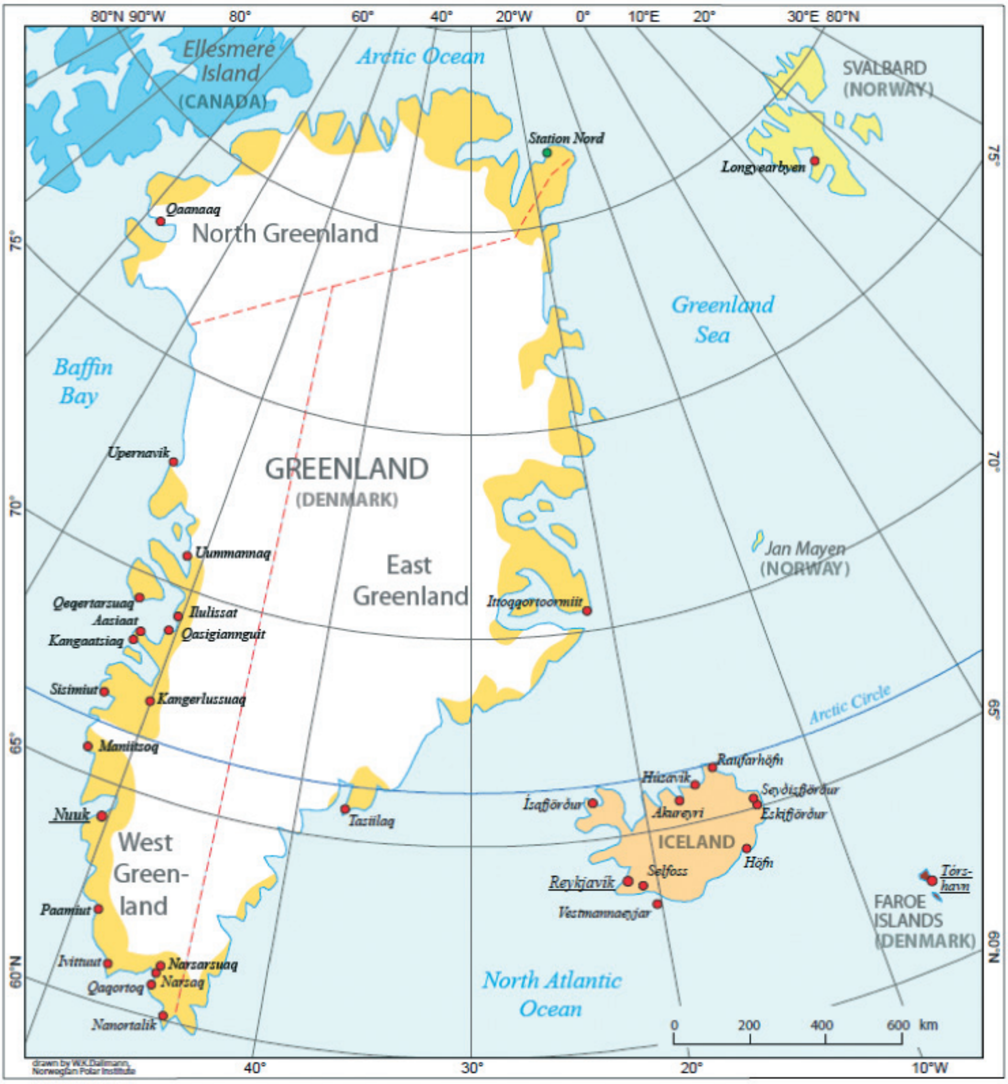

**Figure 4.1.** Map of Greenland, Iceland and Faroe Islands showing major population centres.

### CHAPTER 4.
### GREENLAND

Peter Bjerregaard

### 4.1 Introduction

Greenland is a self-governing part of the Kingdom of Denmark. Apart from certain areas such as foreign policy, defence and currency, the country is governed by the Greenland Government (*Naalakkersuisut*), which implements laws passed by its Parliament (*Inatsisartut*). The largest island in the world, Greenland is sparsely populated along the southwest coast with a few communities on the east coast and in the northwestern corner (**Fig.4.1**). The population of Greenland numbered 56,615 on 1 January, 2011. Official agencies use the concept of "born in Greenland" as a proxy for Inuit ethnicity and 89% of the population was "born in Greenland" (Statistics Greenland, 2011). According to population surveys about 90% of the population is indigenous Greenlanders of Inuit descent.

There are a total of 81 communities, all situated on the coast; 17 are called towns and the remainder are known, interchangeably, as villages or settlements. A town is by definition the largest community in each of the former municipalities. The population of towns ranges from 475 to 15,000 inhabitants while villages have population less than 10 to 550. In the towns are located schools, health centres, churches, main shops and administrative offices of the district. Only 15% of the population live in villages, while more than 25% live in the capital Nuuk. A recent reform reduced the number of municipalities from 17 to 4.

There are no roads between the communities and all travel is accordingly by public aircraft (airplane and helicopter) or public or private boat. The public boat transport is increasingly being replaced by air travel, but air travel is expensive and weather sensitive. There are five weekly flight connections between Denmark and Kangerlussuaq, which is the international airport of Greenland, two weekly connections with Iceland, and regular service to towns and villages within the country. Few consumer goods are produced in the country. Most commodities are transported by cargo ships from Denmark. Fresh products are transported by air and are accordingly expensive.

The health care sector was transferred to Greenland's responsibility in 1992. While there is a certain cooperation regarding health issues between the governments of Greenland and Denmark as well as at the operational level, Denmark has no responsibility for health care or prevention in Greenland, and Danish law and regulations are no longer valid in Greenland. Patients are regularly transferred from Greenland to Denmark for advanced tertiary care but the receiving hospitals are paid for their services by the Greenland health care system.

The overall responsibility for the health care system lies with the Minister of Health

(*naalakkersuisoq*) of the Greenland government who is the political leader of the Department of Health. At the bureaucratic level, the Department of Health is headed by a permanent secretary. Under the Department of Health is the Section for Health and Prevention whose overall responsibility is running the health care system and implementing the Public Health Programme (*Inuuneritta*). The Chief Medical Officer (*Landslægen*) reports directly to the Minister of Health, with responsibilities of overseeing the health care system, handling of medical malpractice, licensure of health staff, and collecting and reporting some health statistics, but not strategic development or actual health care administration.

In the Greenland context, health care does not include long term care (nursing home care and home care). Laws concerning health are issued by the Greenland parliament. This convention of separating health care from social care is consistent with the practice in other Nordic countries.

### 4.2 Organizational Structure and Financing

All health care, including prescribed drugs and dental care, is free at the point of delivery to all residents of Greenland. Outside the public health care system there is the odd private dentist or physiotherapist but there are no private doctors or hospitals. The annual budget is stipulated by a yearly fiscal law. The municipalities pay for social security and care of the elderly and disabled but as mentioned above this is not considered health care in the Greenland context.

In 2011, the budget for health care in Greenland amounted to a total of 1,184 million Danish kroner (DKK). This includes costs for treatment of patients in Greenland and abroad, maintenance of infrastructure, medical travel within Greenland and to Denmark, dental care, prevention and health promotion, drugs and administration. The budget corresponds to 20,900 DKK per capita. There has been a steady budget increase of 67% in fixed prices since 1994; 30% in price adjusted cost.

In 2008, the per capita expenses for health care amounted to 2,533 Euros (NOMESCO, 2010). This was lower than in the other Nordic countries despite the fact that it is relatively more expensive to offer health care in a geographically and climatically challenged country like Greenland compared to Europe. About 12% of the total expenditure was used for medical travel (patients and staff) and shipping of supplies. Almost all (97.5%) of health care expenditures was publicly financed. Greenland is unique among circumpolar regions in that its per capita health expenditures is only about 70% of Denmark's, whereas all other regions have higher per capita health expenditures than their respective national expenditures.

Greenland is not yet financially self-sufficient, and 29% of total government revenues come as a transfer from Denmark. This block transfer is not earmarked for specific purposes and is at the disposal of the Greenland government as it sees fit (Lund, 2011).

### 4.3 Delivery of Health Services

The Greenland health care system is physician based. It has a Danish legacy but is working its way from a decentralised system staffed with Danish professionals towards a regional system staffed with Inuit professionals. A

health care reform was formally introduced in Greenland on 1 January 2011 (see Section 4.5). Among other things, this reform includes reducing the number of hospitals from 16 to 5 regional hospitals, converting the remainder into health centres, and increasing the use of telemedicine (Fig.4.2). In this context, regions are equivalent to the newly configured municipalities with the exception of one municipality which has two regional hospitals. Since the process will take several years to implement, the existing health care system based on the old structure is described in some detail below.

***Primary care***

Before the organizational reform of 2011, Greenland was divided into 16 health districts roughly equivalent to the (old) municipalities. Outside Nuuk, each district has a small

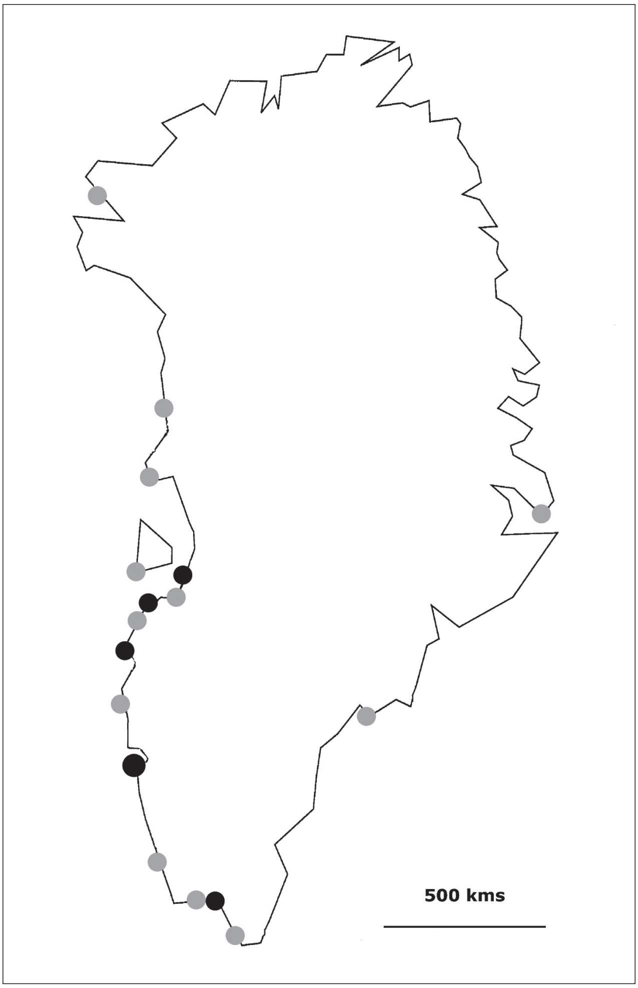

**Figure 4.2.** Distribution of hospitals in Greenland.
Note: Black dots represent hospitals remaining after the 2011 health reform; grey dots represent hospitals discontinued after health reform.

hospital or health centre staffed by one to five district medical officers. The hospitals and health centres offer primary and emergency care, and are equipped with an operating theatre, X-ray equipment, delivery ward, and laboratory. The district medical officers handle uncomplicated deliveries, legal abortions, common internal diseases and psychiatric cases, but under unfavourable weather conditions they must be prepared to handle complicated cases including major life-saving surgery. For example, most operations for extrauterine pregnancy, appendicitis and trauma are performed in the districts. Patients with complicated non-acute diseases are referred to visiting specialists, to the central hospital in Nuuk or to a university hospital in Denmark. All districts have a dental clinic usually located at the hospital or the school.

Health care is offered free of charge to all residents but varies according to community size. In Nuuk, the primary health clinic is separate from the hospital although situated on the same premises. It has its own staff. In the 15 other towns, the primary health clinic is integrated in the hospital or health centre and share the same staff. In the villages, health care is offered depending on community size.

In the larger villages (about 4.5% of Greenland's population) a registered nurse and a health aide are responsible for primary health care on behalf of the Chief District Medical Officer. In medium sized villages with a population between 70 and 300 (about 9.5% of Greenland's population), a registered health aide is employed full time while in small villages with less than 70 inhabitants (1.2% of Greenland's population), a locally trained health assistant is paid a certain number of hours per week to keep the village medicine chest and to administer basic treatment and provide health advice. The village clinics vary from little more than one room adjacent to the living quarters of the village health worker to a separate building with a consultation room, a waiting area and a room with beds for acutely ill patients waiting to be transferred to the hospital. The equipment in the village clinics is basic but all clinics have a telemedicine console for real time consultations with the hospital staff in town. All villages are visited regularly by staff from the district hospital (physicians, nurses, midwives, public health nurses etc.) who offer primary health care, prenatal care, other preventive care and vaccinations.

While the Greenland health care system is fairly successful in managing acute illness and injuries, the organization of continuous care for patients with chronic diseases (e.g. diabetes or hypertension) health promotion and disease prevention suffer from the lack of permanent staff. Such programs are not carried out to any great extent by staff on short term or even year long contracts and they tend to fall on the shoulders of the relatively few permanent physicians and nurses. Within the last few years a promising program for managing diabetics has been established in Nuuk, and it is planned to extend the principles of this program to other chronic diseases and to other regions.

Dental care is provided by public dentists assisted by dental hygienists. Care is focused on systematic preventive care and treatment of children including orthodontics. Adults are offered basic treatment including fillings, extractions and dentures.

Pregnant women are followed regularly during pregnancy by trained midwives or in their absence by trained health aides. A risk score has been developed by the obstetrical department in Nuuk, and women considered at risk for complications are transferred to the obstetrical department in Nuuk to deliver their babies. All other deliveries occur at the regional hospitals. Delivery at home or in the villages is discouraged for normal as well as at risk pregnancies. A discussion about whether to continue offering possibilities for planned deliveries outside the regional hospitals and the obstetrical department at Queen Ingrid's Hospital in Nuuk is ongoing.

***Hospital services***

Only in Nuuk is primary health care separated from secondary care, the latter being offered by Queen Ingrid's Hospital, which is both the local hospital for Nuuk and referral hospital for all of Greenland. The hospital has around 150 beds in medical, surgical, intensive care and psychiatric wards. The hospital is staffed by specialists covering a number of medical specialties: psychiatry, internal medicine, orthopedic surgery, gynecology, obstetrics, general surgery, pediatrics, dermatology, anaesthesiology, and radiology. The specialists visit the health districts outside Nuuk on a regular basis. If there is a need for more specialized hospital treatment, patients are referred to hospitals in Denmark, often to the University Hospital in Copenhagen. Medical travel, hospital care and living expenses are paid for by the Greenland health care system. Examples of treatment that currently takes place in Denmark include specialized cancer treatment, advanced and invasive cardiac diagnostics, neurosurgery, thoracic surgery, and organ transplantations.

***Public health***

The Greenland Public Health Programme *Inuuneritta* was inaugurated in 2006. It is an umbrella for disease prevention and health promotion that focuses on 7 specific themes and targets two vulnerable population groups: children and youth; and the elderly. The themes are alcohol and drugs; violence and sexual assaults; suicide; diet and physical activity; reproductive health; smoking; and dental health. Some of the themes build on already existing strategies, notably suicide and dental health, while for others, strategies and specific interventions have yet to be developed. The leadership of the program rests with the Section for Health and Prevention which contains the former Office for Prevention (PAARISA) which for decades has designed information campaigns and been responsible for various kinds of prevention and health promotion, often in relation to reproductive and dental health.

The objectives of the Public Health Program are somewhat diffuse and short of specific targets. A set of 65 indicators has been developed in order to be able to monitor the program. The funding of the first phase is secured in the fiscal law until 2012 with a modest 8 million DKK per year running costs. The total cost of the population-oriented (as opposed to individual-oriented) public health in the Department of Health is listed under several subheadings of the annual budget. With considerable uncertainty it can be estimated at 25 million DKK in 2011, which amounts to 2.1% of the total health expenditures.

*Inuuneritta* was externally evaluated in 2011 and substantial changes were suggested for an improved program. In addition to strengthening the political leadership and administrative structure, the evaluation recommended that the program should focus on only two themes: children, youth and families; and reducing the risk factors for chronic disease including diet, smoking, alcohol, and physical activity (Kamper-Jørgensen, 2011).

Public health services for individuals include preventive clinics for pregnant women, well baby clinics, and childhood immunizations. The childhood immunization programme currently consists of vaccination against tuberculosis, hepatitis B, diphtheria, tetanus, whooping cough, polio, pneumococcal disease, *Hemophilus influenza* type b infection, measles, mumps, rubella, and human papillomavirus (girls only).

General disease prevention and health promotion is carried out by the local prevention officer (*forebyggelseskonsulent*) who are backed up by the central preventive office (PAARISA). They offer smoke cessation programs and run information campaigns, in addition to other prevention and health promotion activities.

Disease surveillance is the responsibility of the Chief Medical Officer, who collects and publishes reports from the health districts including statistics on births and deaths, notifiable diseases, cancer, and legal abortions (Landslægen, 2009).

### 4.4 Patient Pathways

A patient from a village with a novel medical condition will normally consult the village health worker. She will decide whether to treat the patient immediately or to consult with a nurse or a physician at the local health centre. Based on the condition of the patient, the physician or nurse will recommend local treatment or recommend that the patient be seen by a doctor on his next visit to the village, or refer the patient to subacute or acute treatment at the health centre, by public transport or if needed by specially arranged evacuation (usually by helicopter or speedboat). Further referral to a regional hospital or the central hospital will take place as needed. Patients living in or visiting one of the towns will consult the physician directly in the open clinic.

Medical evacuation is used considerably less than in the Canadian north and is seen as a benefit, not as a right. Evacuation or publicly paid referral to a larger hospital is only possible at the discretion of the District Medical Officer. Under special circumstances a relative may accompany the patient.

### 4.5 Health Care Reform

The purpose of the health care reform of 2011 is to adjust the health care system to the population shift from smaller villages to larger towns and to create a more effective service delivery, i.e. "more health for the same money." The reform will also facilitate Greenland's coping with the global trend of increasing specialization of diagnostics and treatment. Over the years it has become increasingly difficult to attract qualified staff to the many small hospitals staffed with only one or two physicians as well as to maintain an up to date quality of care. With one exception the five new health regions are geographically similar to the four municipalities that were established in 2009. The five health regions are Avannaa with a regional hospital in Ilulissat; Disko with a regional hospital in Aasiaat; Qeqqa with its hospital in Sisimiut; Sermeersooq with Queen Ingrid's Hospital in Nuuk as its regional hospital; and Kujataa with a hospital in Qaqortoq. Each regional hospital is responsible for health care in 1-3 other health districts including 1-3 towns and 6-24 villages (Departementet for Sundhed, 2010).

Telemedicine will increasingly be used to overcome the long distances and the centrally located specialist knowledge. All service delivery points, even in the smallest villages, have recently been supplied with advanced telemedical equipment.

Queen Ingrid's Hospital continues to be the local hospital for Nuuk and the referral hospital for the whole country. The medical specialists at the hospital oversee treatment in all regions and create guidelines for treatment in collaboration with the regional hospitals. They visit towns and villages on the Coast in order to deliver specialised treatment.

The regional hospitals will be centres for health care in their region. Most physicians will eventually work at a regional hospital and perform outpatient clinics in health centres in other towns and in village clinics. They will be family physicians with additional training in certain emergency surgical procedures such as appendectomy and caesarean section. The regional hospitals will also have trained midwives, public health nurses, physiotherapists, dieticians and laboratory technicians.

The current small hospitals will continue to function as such but will eventually develop into district health centres. The precise spectrum of diagnostics and treatment will depend on the qualifications of the staff. It will not be necessary to have physicians at the district health centres at all times, which means that vacancies shorter than 3 months will not be filled. In those circumstances medical care will be provided by physicians from the regional hospitals. The health centres currently staffed by a single physician and health centres that face problems recruiting physicians will receive medical care from the regional hospital supplemented with the care from nurse practitioners and an increased reliance on telemedicine. The nurse practitioner is a new staff category in Greenland.

The total budget for the health care system is not anticipated to change as a consequence of the reform but it will most likely result in less money being spent on salaries and benefits because of vacancies left unfilled and more being spent on medical travel, in particular travel of staff. Patient pathways will not change substantially in the future, but with improved diagnostics at the local level it is reasonable to expect that among patients who will be referred, a higher proportion will actually need to be referred – from villages to health centres, from health centres to regional hospitals and from regional hospitals to the central hospital in Nuuk.

### 4.6 Human Resources Management

Physicians, dentists, midwives, and laboratory technicians are trained and authorised abroad, mostly in Denmark. Nurses and health aides are trained in Greenland. The reliance on Danish health professionals creates language problems since a majority of the population, especially among the elderly, is not sufficiently proficient in Danish language and few physicians can speak any Greenlandic. Accordingly, substantial resources are spent on interpreters. The training of nurses in Greenland started in 1996 and since then approximately 100 - mostly Inuit - nurses have been trained, while 53 are currently in training. The nursing program is now university- based with nurses graduating with a bachelor's degree.

**Table 4.1** shows the health staff in Greenland. With 17 physicians per 10,000 inhabitants, Greenland lags significantly behind the Scandinavian countries. Greenland cannot cover its need of physicians, midwives, dentists and nurses and these professionals are often immigrants from Denmark or other Nordic countries. There is an increasing proportion of Greenlanders among nurses and physicians but the fact that most physicians and nurses are still Danes with a very limited knowledge of the Greenlandic language sets a natural limit to their interaction with the patients, the majority of who have little knowledge of the Danish language. The large proportion of outsiders at the higher levels of health professions creates further challenges as many choose to stay for only a few years only or often for an even shorter time.

Training of local residents as nurses and health aides is a high priority for the government, as is the recruitment of highly skilled staff, including physicians, midwives, dentists, and nurses to complement the locally trained nurses. The training of nurse practitioners is being planned at the school of nursing in Nuuk.

It is a constant struggle to keep the positions filled and it is often necessary to employ short term staff to fill vacancies of a few months' duration or even less. This is particularly the case in the small hospitals with only one physician position where vacancies of one or a few weeks are not uncommon. For physicians, the ratio of permanently filled positions to vacancies is about 1:1 on a countrywide base.

**Table 4.1.** Number and rate (per 10,000) of health care workers in Greenland.

| | Number | Rate |
|---|---|---|
| Physicians | 98 | 17.3 |
| Midwives | 20 | 3.5 |
| Nurses | 191 | 33.8 |
| Dentists | 31 | 5.4 |
| Health aides | 253 | 44.8 |
| Other skilled | 339 | 60.1 |
| Unskilled | 288 | 51.1 |
| Total | 1220 | 216.0 |

Source: Sundhedsledelsen (unpublished data)
Note: head count as on December 31, 2009. Visiting specialists are excluded. There are considerable discrepancies between these figures and those published by NOMESCO.

### 4.7 Conclusions

Health care in Greenland is challenged by the many small and geographically scattered communities. Medical travel is expensive and occasionally impossible due to weather conditions. It has become increasingly difficult to recruit suitably qualified professional staff, including physicians, nurses, midwives and others. There are not sufficient Inuit health professionals, and the majority of physicians continue to be recruited from abroad, in particular from Denmark. In spite of the logistical challenges and the comparatively poor health status in Greenland, the Greenland economy does not generate sufficient public revenues to allow health expenditures to be at par with other Nordic countries. The aim of the 2011 health care reform is to spend the available health budget in a more efficient way in order to deliver better health services for the same budget. One way of doing this is to reduce the cost of bringing in staff from abroad for short term vacancies, for instance by eliminating the expectation that all of Greenland's 16 towns are entitled to at least one permanent resident physician. Telemedicine is a key instrument in overcoming the distances and binding the health care system together. A supplementary strategy could be to increase the focus on disease prevention and case management of chronic disease, which in the long run would reduce morbidity.

### CHAPTER 5.
### ICELAND

Tinna Laufey Ásgeirsdóttir

### 5.1 Introduction

Iceland is a representative democracy and a parliamentary republic in the middle of the North Atlantic Ocean, with a population of 320,000 and a total area of 103,000 km² (Statistics Iceland, 2011) (**Fig.4.1**). The Icelandic populace is very homogeneous and the country has been continuously occupied since it was first settled by the Norse in the 9th century. Iceland has traditionally ranked as one of the most developed countries in the world according to the United Nations' Human Development Index (UNDP, 2011).

The capital of Iceland is Reykjavik, the country's only city. However, the greater capital area includes other nearby towns and urban areas located in the southwest of the island. In total, more than half of the population lives in this region. The rural population is located in the countryside and villages along the coastline of the country. Akureyri, the largest town outside of the capital area, is inhabited by about 17,000 people. This translates into a small population distributed across a far-flung land area. It follows that there are areas that are somewhat remote, but a separation of specific northern regions is not of value in Iceland as is the case in many other circumpolar countries. Furthermore, Iceland lies in the path of the North Atlantic Current, which makes the climate of the island more temperate than would be expected for its latitude just south of the Arctic Circle.

The Icelandic health care system differs from those of the other circumpolar countries in some fundamental structural aspects. Specifically, the Icelandic health care system is more centralized in its governance structure, management, regulation, delivery and financing than other circumpolar countries. The Minister of Welfare oversees practically all health affairs. While Iceland is divided into regions, counties and municipalities, the role of local authorities in health care is negligible.

In 2011 the Ministry of Health [*Heilbrigðisráðuneytið*] was merged into a new Ministry of Welfare [*Velferðarráðuneytið*] with responsibilities for health care, family welfare, housing, labour market and gender equality. The medical director of health [*Landlæknir*] advises the Minister of Health and the government on all health matters and oversees the Directorate of Health [*Landlæknisembættið*]. It monitors the quality of health services, prescription drug use, and health professional education; collects health information and monitors public health; handles public complaints; and also licenses and regulates the health professions.

### 5.2 Organizational Structure and Financing

The Icelandic health care system can be classified as an integrated single-payer healthcare system financed by general taxation. It is deeply rooted in the Nordic model of the welfare state. Thus, one of the principal objectives of the health-care system is to improve health, irrespective of the patient's financial means. While several policies aim to reduce income-related inequalities in health, heavily subsidized medical care is by far the most important public policy as measured by the financial resources devoted to it.

Funding by local taxes is not used to any extent and the involvement of local authorities in financing is limited to exceptional instances, for example some contribution to the building costs of local nursing homes. Similarly, local authorities have played a very limited role in the management and delivery of health care services. Still, in line with other Nordic countries, Iceland has recently implemented what on the outset seems to be decentralization by dividing the country into seven health care regions. The purpose has, however, not necessarily been to devolve power to the regions as much as to induce institutional mergers and increased cooperation and consolidation *within* the areas in mind. There are obvious challenges regarding the size of the population and in a rapidly specializing world of medicine, economies of scale can be difficult to realize within sparsely populated areas. Thus the legislation that introduces health-care regions states:

*"Health care institutions that provide general health care in each of the health-care regions shall cooperate in configuring services in the region. The minister of health can, in cooperation with municipalities and The Association of Local Authorities in Iceland, decide to merge health-care institutions within a health-care region with regulations. Despite the separation of the country into health-care regions, patients should have equal access to health centres or other health care institutions where most convenient at each time."* (Alþingi, 2007; author's translation).

The creation of health care regions was largely motivated by the need to promote mergers and increase cooperation among institutions. In fact, there has been a pronounced trend towards consolidation through mergers for more than a decade. In 1999, for example, 10 institutions in the east of Iceland, including health centres, hospitals and nursing homes were merged. The main purpose of such mergers has been to increase capacities in the provision of quality care. However, this institutional consolidation may have decentralized the system indirectly, as the larger institutions are better able to control more of their daily decision making and personnel management than the smaller organizations they replaced. As such, the larger organizations are more robust in dealing with the central government.

Nevertheless, the change does not shift financing responsibility away from the central government to other levels of governance, such as municipalities. In fact, there has been no systematic decentralization of either financing or administration from the central government to the municipalities.

The Icelandic health care system is mainly financed through taxes, even though the patient pays modest user fees at the time of service. It should be noted, however, that dental care is only subsidized for children, the elderly, or for treatment for birth defects, oral diseases or injuries. Consequently, dental care comprises a large portion of private expenditures on health (Vilhjálmsson and Sigurðardóttir, 2003).

Despite not being directly related to earnings, some groups with limited ability to generate income, such as the disabled or retired people, pay a lower fee for health care services. Co-payments, however, do not generally take into account the patient's earnings. Around 80% of total expenditures on health care in Iceland are publically financed. The remaining 20% are almost exclusively financed by out-of-pocket payments (OECD, 2011).

Due to the extensive public medical services, private or employer-provided health insurance hardly exists in Iceland. While neither is prohibited by law, there is little incentive to purchase such insurance. The *Health Services Act* and the *Act on the Rights of Patients* state that "every citizen has the right to the best health services available at all times, for the restoration and protection of their mental, physical, and social health" (Alþingi, 2007).

The rise in per capita health care expenditure has been exceptionally rapid in Iceland over the last few decades, although the rapid growth has been curbed considerably in recent years, accounting for between 9% and 10% of gross domestic product (OECD, 2011). The sparsely distributed population may be a partial reason for the country's high expenditures on health, as economies of scale and scope are difficult to achieve. Furthermore, ensuring access to health care, especially during the winter months, does require more health services delivery sites and increases the cost.

**Table 5.1.** Health expenditures in Iceland, 2005-09 mean, by financing agent, function, and provider.

| | 2005-09 | % TEH | % GDP |
|---|---|---|---|
| Total health expenditure | 120412 | 100 | 9.3 |
| Financing agent: | | | |
| [HF.1] General government | 98883 | 82.1 | 7.6 |
| Function: | | | |
| [HC.1] Services of curative care | 63868 | 53.0 | 4.9 |
| In-patient curative care | 29011 | 24.1 | |
| Day cases of curative care | 6327 | 5.3 | |
| Out-patient curative care | 27311 | 22.7 | |
| Services of curative home care | 1219 | 1.0 | |
| [HC.2] Services of rehabilitative care | 6730 | 5.6 | 0.5 |
| In-patient rehabilitative care | 2596 | 2.2 | |
| Day cases of rehabilitative care | 795 | 0.7 | |
| Out-patient rehabilitative care | 3339 | 2.8 | |
| [HC.3] Services of long-term nursing care | 23035 | 19.1 | 1.8 |
| In-patient long-term nursing care | 22400 | 18.6 | |
| Day cases of long-term nursing care | 636 | 0.5 | |
| [HC.4] Ancillary services to health care | 2673 | 2.2 | 0.2 |
| [HC.5] Medical goods dispensed to out-patients | 19987 | 16.6 | 1.5 |
| *Total personal health care expenditure HC.1-HC.5* | *116294* | *96.6* | *9.0* |
| [HC.6] Prevention and public health services | 1845 | 1.5 | 0.1 |
| [HC.7] Health administration and health insurance | 2274 | 1.9 | 0.2 |
| Provider: | | | |
| [HP.1] Hospitals | 48673 | 40.4 | 3.8 |
| [HP.2] Nursing and residential care facilities | 13723 | 11.4 | 1.1 |
| [HP.3] Ambulatory care providers | 33009 | 27.4 | 2.5 |

Source: *OECD Health Data 2011*. Note: Currency in Icelandic krónur (ISK); Iceland's total health expenditures (TEH) as reported to OECD are identical to its total current health expenditures, i.e., TEH less capital formation.

Iceland's demographic situation has an impact on its health care system. Iceland has the youngest population of all the Nordic countries, with only 12% of the population aged 65 and above. It also has the highest fertility and a favourable dependency ratio. Surprisingly, this has not translated into lower utilization of long-term care services. Inpatient long-term care expenditures as a proportion of total health expenditures amounted 19% during 2005-2009, comparable to other Nordic countries with a much higher proportion of the elderly in the population (**Table 5.1**). The level of expenditures remains high even as the number of long-term care beds within hospitals has decreased. Iceland's fertility is predicted to decline in the near future, posing challenges to the health care system (Statistics Iceland, 2008).

Icelandic Health Insurance (*Sjúkratryggingar Íslands*) is a recently established public institution that is meant to administer and purchases health care services on behalf of the central government. The main goal of the institution is to strengthen the state's role as a buyer of health services, leaving this institution as the only buyer of health services while introducing a purchaser-provider split between it and the government. The institution is meant to conduct cost analysis, the first time that such an analysis will be systematically carried out in Iceland, a recommendation previously made in an OECD Economic Survey (OECD, 2008). This institution is intended to execute its role by taking into consideration economic efficiency and equal access (Alþingi, 2008).

When founding Icelandic Health Insurance, policy makers were inspired by the Swedish purchaser-provider split of the early 1990s. Given the mixed results in Sweden, it remains to be seen what effect these reforms will have on fixed and activity-based funding in Iceland.

### 5.3 Delivery of Health Services

While there is a general consensus in Iceland about public financing of the health care system there has been considerable debate about the most effective ways to administer and deliver health care. This debate has to some extent been a response to health care developments in neighbouring countries, where there have been similar debates concerning the question of private delivery within publicly financed systems.

***Primary care***

Although many countries have expanded patient choice in the past, the freedom to seek services directly from a specialist has been and continues to be, a pronounced feature of the Icelandic system. It is thus difficult to discuss primary care and first points of contact without discussing the role of specialists.

As mentioned above, recent health care reforms in Iceland have in multiple ways increased centralization, where the government has taken over practically all responsibilities that local authorities previously oversaw. A notable example of increased centralization is primary health care in the capital area. One primary care institution now administers almost all primary care services in and around Reykjavik. Previous to the change, primary care was provided by independent practitioners based in health centres.

In the capital area, the Primary Health Care of the Capital Area (*Heilsugæsla höfuðborgars-*

*væðisins*, hereafter PHCCA) operates fifteen health clinics, only one of which is reasonably independent in managing its own affairs under the PHCCA board. The PHCCA clinics offer general medical and nursing services, maternal and child health services, school health, vaccinations for adults, and health care for the elderly among other services. The clinics' activities are directed towards inhabitants of specific neighbourhoods within the capital area. A few general practitioners still see patients privately, the last remnants of the old system that has survived despite a government policy that clearly favours a centrally organized network of health clinics.

In the capital area, there is one health centre that is privately run, without any direct administrative affiliation with PHCCA. It is, however, financed publically under a contract with the Ministry of Welfare. In the tender process for this project, it was specified that the goals were to increase access and efficiency while guaranteeing comprehensive services. The contract involves an incentive scheme where some of the payments are based on services given. The outcome of this experiment has been examined and reported. Based on the Ministry of Health's own evaluation, the results from this experiment in private delivery were favourable in the three areas examined – cost, performance and user satisfaction (Heilbrigðisráðuneytið, 2008).

Health centres providing primary care are located throughout the country, some jointly run with small hospitals or health institutions. The centres outside the capital area are all publically funded and administered, based on fixed budgetary dispensations. Even though primary care centres are distributed to ensure geographic access, rationing through waiting times can limit access, a concern felt particularly in Reykjavik.

The debate on the appropriateness of private vs public provision in the western world seems to generate greater political debate with regard to specialist rather than primary care. It is thus interesting that Icelanders have private provision of specialist care but public provision of primary care. There are formidable obstacles for private practice by general practitioners, as there are limited opportunities besides applying for positions at the publicly managed health centres. Thus the number of general practitioners and the quantity of primary care service is publically determined. In contrast, specialists can start practicing without much impediment. They can open private clinics and start servicing patients under the public insurance scheme on a fee-for-service basis negotiated between the government and the Medical Association. Specialists do not have to wait for a position to become available or negotiate the amount of their services they would like the state to purchase. There is, however, an upper limit on services provided by specialties as a whole, and the entry of new providers can potentially limit the amount of work (and income) available to other providers within the same field. However, as a result of the lack of constraints, specialist practices have grown rapidly, and due to somewhat limited access to general practitioners and extensive patient choice, it seems that specialists provide many services that in other countries are provided by general practitioners. The distribution of specialist services seem quite random with some areas apparently over serviced while others are lacking in the same type of specialist care. This is consistent with a lack of government policy and direction and supplier-induced demand, though the latter has not yet been examined.

In Iceland, the mainly salaried general practitioners have few economic incentives to attract patients and the primary care sector is marked by low productivity (Heilbrigðisráðuneytið, 2008). In theory, general practitioners are supposed to be the patients' first point of contact within the system. In reality, specialists and emergency care units are involved in a substantial number of what could be classified as primary care services.

The interplay between a highly regulated primary care system with less than optimal productivity and the much less regulated services of specialist-provided ambulatory care, financed on a fee-for-service basis, is likely to lead to outcomes that are not consistent with the idea of primary care being the patients' first point of contact within the system.

### *Hospital services*

Iceland's main hospital is located in Reykjavik. Akureyri also has a hospital. In addition there are 6 regional hospitals and 16 health institutions dispersed around the country. This number is the result of recent mergers. The National University Hospital (*Landspítali*) was created from two older hospitals in Reykjavik, and it has recently also taken over a small hospital in the adjacent town of Hafnafjörður.

Hospitals are funded on a fixed (global) budget. Landspítali alone takes up one third of the Icelandic health care budget and is a state-run hospital. Similarly, most Icelandic health-care professionals are salaried employees. Inpatient hospital care is funded without any copayment by patients whereas ambulatory hospital care does. The greatest cost pressure may be the overuse of inpatient care, when other forms of treatment could be more cost effective.

Similarly, Iceland has not developed lower-intensity care to the same extent as many other countries, still relying more on inpatient care than ambulatory care and day surgery. In terms of expenditures Iceland allocates its health care budget quite conspicuously to higher levels of care (**Table 5.1**).

Out-patient hospital care and home care could surely be used to a greater extent where inpatient services are currently employed. It has been suggested that user fees for inpatient hospital care should be considered to encourage the use of alternative services instead of expensive hospital resources when appropriate (OECD, 2008). The focus on institutional long-term care as opposed to less costly measures such as home care is also a feature that sets the Icelandic health care system apart from its Nordic counterparts.

### *Public health*

Health outcomes are generally favourable in Iceland with life expectancy among the very highest in the world and infant mortality among the very lowest in the circumpolar world, indeed the whole world (**Fig.1.7**). A heavy emphasis on prevention and community health is something that the other Nordic systems have in common, which Iceland shares, though perhaps not to the same extent. In 2011 The Public Health Institute of Iceland (*Lýðheilsustöð*), created in 2003 to promote public health, monitor the population health and evaluate public health programs and policies, was merged into the Directorate of Health and its role and activity level under this new arrangement remains to be seen.

Substantial progress has been made on many fronts in terms of lifestyle choices. Smoking has for example declined substantially in the past decades and some progress has been made with regard to substance abuse. There are however still areas of concern. Despite the fact that the average per capita consumption of alcohol in Iceland is lower than in most other European countries, drinking habits of Icelanders have traditionally differed from those in neighbouring countries and are characterized by lower frequency of consumption. However, when alcoholic beverages are used, they are often consumed to the point of intoxication, and binge drinking of hard liquor is quite common. It should be noted though that these patterns have been slowly changing in recent years, as the rate of binge drinking is decreasing (Ólafsdóttir 1998).

The most pressing public health concern is obesity. Currently, Icelanders are among the heaviest in Europe. The increase in weight has been quite rapid, from 7.5% of the population being obese in 1990 to over 20% in 2009 (Steingrímsdóttir et al., 2002; Ásgeirsdóttir, 2007; Gísladóttir et al., 2009).

### 5.4 Human Resources Management

The rate of physicians (3.7 per 1,000 people) in Iceland is fairly high by global standards although not out of the ordinary among Nordic countries (**Table 1.8** and **Table 1.9**). However, the population of doctors in Iceland is much skewed towards specialists, as shown in **Table 5.2**. General practitioners accounted for less than 20% of the physicians practising in the country.

**Table 5.2.** Number of practising physicians in Iceland, 2005-09 mean, by category and place of employment.

| Category | Number | % |
|---|---|---|
| Practising physicians | 1123 | 100.0 |
| General practice | 184 | 16.4 |
| General paediatrics | 17 | 1.5 |
| Obstetrics and gynecology | 36 | 3.2 |
| Psychiatry | 75 | 6.7 |
| Medical group of specialties | 350 | 31.2 |
| Surgical group of specialties | 220 | 19.6 |
| Other | 241 | 21.4 |
| Employed in hospital | 898 | 80.0 |

Source: *OECD Health Data 2011*.

In Iceland the majority of nurses work in the public sector. The largest employer in this respect is Landspítali-University Hospital. Other health institutions and the health care centres falling under the public sector collectively are also employers. Education for nurses in Iceland takes four years for the completion of a BSc degree, as opposed to three years for an undergraduate degree in most other fields of study.

### 5.5 Conclusions

The Icelandic people enjoy good access to high quality health care services. What most decisively separates the Icelandic health-care system from those of the other circumpolar regions is the degree of centralization of the health care system and limited steering of patients within that system. Patients have extensive choices in where they seek treatment. In such a system incentives need to be carefully reviewed. The structure of the cost-sharing scheme could be used more effectively to direct patients to the most appropriate level of care. Currently copayments are not structured according to the cost of the services provided, and copayments for more costly services are often lower than those for less expensive services. Examples of this can be found in the cost-sharing structure of some drug treatments, as well as inpatient versus outpatient care.

Little attention has been paid to cost-effectiveness studies. The need for such data was in fact was expressed in an OECD economic survey on Iceland, which stated that it is "important to improve the cost-effectiveness of health care in Iceland, which seems to be lacking, in order to be better prepared for the unavoidable long-term pressures due to population ageing." (OECD, 2008, p. 86). Due to Iceland's small population, research evidence on which health policy can be based is limited. Health economics and policy studies conducted in the context of Iceland are of particular value to policy makers, who are otherwise left to rely on intuition and experience from people, places, and times that may be far from current Icelandic reality.

### CHAPTER 6.
### NORTHERN NORWAY

Jon Magnussen

### 6.1 Introduction

Norway has total population of 5 million and a population density of 14.6 inhabitants per $km^2$. The country has three levels of government; the central state, 19 counties (*fylker*), and 430 municipalities. General elections are held every four years, with elections for county councils and municipalities phased in between elections for parliament.

Responsibility for primary and specialized health care services lies on different government levels. Thus while the responsibility for primary care is devolved to the 430 municipalities, the central state is responsible for specialized health, but executes this responsibility through four state owned regional health authorities (*regionale helseforetak*, hereafter RHAs).

In this volume northern Norway is defined as consisting of the three northernmost counties of Finnmark, Troms and Nordland (**Fig.6.1**). This region covers roughly one-third of the total area of Norway with a population of around 465,000 and a population density of 4.4 inhabitants per $km^2$. The three counties also constitute the Northern Norway RHA (*Helse Nord RHF*).

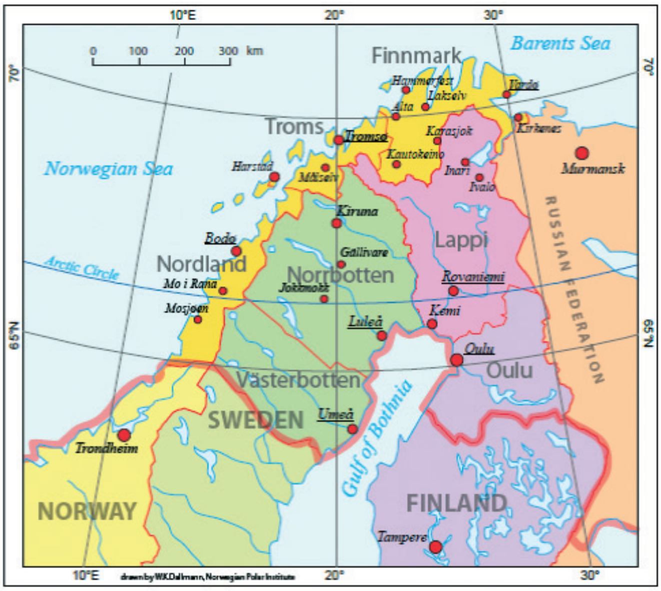

**Figure 6.1.** Map of the northern regions of Norway, Sweden and Finland and major population centres.

### 6.2 Organizational Structure and Financing

In common with other Nordic countries, the responsibilities for preparing health legislations, allocating resources, designing policies, and monitoring their implementation reside at the highest national level in the Ministry of Health and Care Services (*Helse- og omsorgsdepartementet*). Subordinate to the ministry are specialized agencies dealing with issues such as food safety, radiation protection, and the regulation and licensing of drugs. Of relevance to public health are those concerned with disease control, health monitoring and surveillance (including the maintenance of health registries and databases), and the design and implementation of national health promotion and disease prevention programs. These functions are shared between the National Institute of Public Health (*Nasjonalt folkehelseinstitutt*) and the Norwegian Directorate of Health (*Helsedirektoratet*).

Norway has one of the most expensive health care systems in the world, ranking only behind USA and Luxembourg in per capita spending, according to OECD. In 2009, total health care expenditures in Norway amounted to 47,351 NOK per person, accounting for 9.6% of GDP, or 12.4% when oil revenue has been excluded. The private sector is relatively small, accounting for only 16% of total health expenditures. Public sector health care expenditures constituted about 18% of all government expenditures at all levels (OECD, 2011).

The Norwegian system is tax-based and characterized by decentralized governance structure, universal access, services that are (almost) free at the point of use, and predominantly public ownership (Martinussen et al, 2009).

While taxation is the main source of financing for health care, taxes are not earmarked. Thus health care is financed alongside (and thus competes with) other public tasks for funding. Specialized health care is funded directly from the central government, while primary care is funded by the munici-

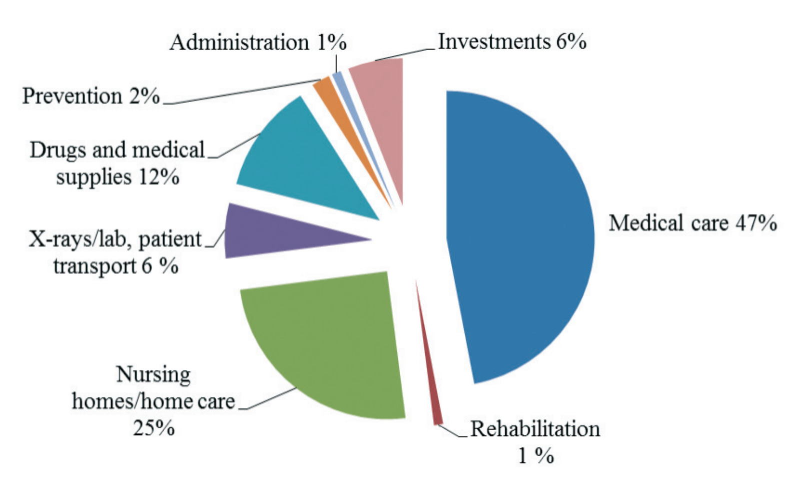

**Figure 6.2.** Distribution of health care costs by type of service in Norway, 2007.
Source: *OECD Health Data 2011*.

palities. Municipalities obtain their income in part from local taxes and in part via tax equalizing grants from the central government. Relative to other Nordic countries Norway has a lower share of autonomously raised local taxes and also a lower share of unconditional intergovernmental grants. Thus Norway ranks as one of the most (fiscally) centralized Nordic countries (Sellers & Lidstrøm, 2007; Rehnberg et al, 2009). **Fig.6.2** shows the distribution of health care costs by type of services in 2007 for Norway. **Table 1.4** compares per capita health expenditures for the whole of Norway with the three northern counties.

**Fig.6.3** shows the distribution of funding between the national government (consisting of state transfers, direct funding and reimbursement from the national sickness fund), the municipalities and individual out of pocket payment for the different types of services. These figures are national averages, however since both municipal funding and state funding is based to a large extent on needs adjusted capitation, there will in general not be large geographical differences in these figures. To the extent that differences exist they may be the result of differences in municipal tax income, differences in local priorities or differences in efficiency. Note, however, that the way expenses are registered in the system of health accounts leads to some inconsistencies. For example, there are no recorded administrative costs at the municipal level, and instead these will be included in other categories of health care costs or excluded altogether. **Fig.6.3**, therefore, should be seen as a rough description of the distribution of costs among payers and types of services on the national level.

### 6.3 Delivery of Health Services

The health care delivery system in northern Norway is not substantially different from that in the remaining parts of the country. On the other hand, when providing services to the population in northern Norway, central authorities must take into account certain

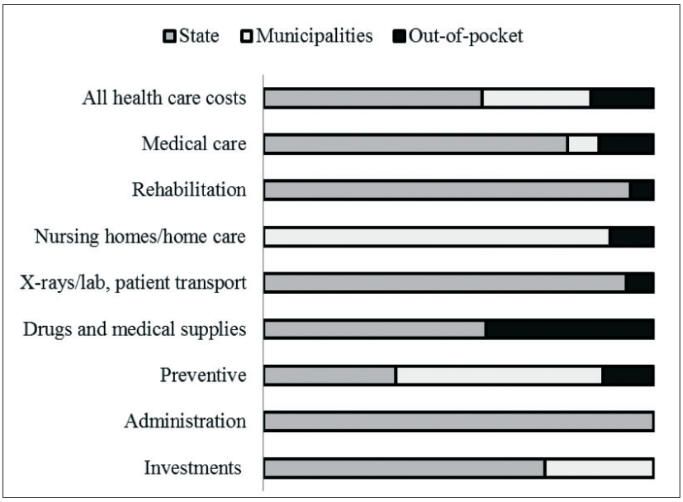

**Figure 6.3.** Share of health care costs by payer in Norway, 2007.
Source: *OECD Health Data 2011*.

special characteristics: the indigenous population, sparsely populated remote areas, and climate conditions.

***Primary care***

Primary health care and long term care is the responsibility of the municipalities. There are 430 municipalities in Norway, with a population ranging from 215 to 600 000 (the capital Oslo). Average population size is around 10 000 inhabitants and as many as 234 municipalities have less than 5000 inhabitants.

Municipalities are governed by elected municipal councils and financed mainly from local taxes and central tax equalizing grants Municipalities are free to allocate resources between public services such as education, health care, child care, social services and other municipal tasks. However health and social services consume most of the municipal budget; more than 40 % of municipal employees are in the health and social services sector.

General practitioners (GPs) are all self-employed and thus represents an element of privately provided care in an otherwise publicly dominated system. Under the current system GPs serve a specific patient list. Each municipality has a number of GP contracts based on the size and composition of the population, thus GPs will need to get a contract with a municipality in order to receive payment from public sources.

GPs receive 30 % of their income on a capitation (list size) basis and 70 % on a fee for service basis. The fee for service consists of patient co-payments and refunds from the national sickness fund. Financing of long term care is through global (fixed) budgets, but with substantial co-payment for patients living in institutions.

In 2001 the Regular General Practitioner scheme (*fastlegeordningen*, hereafter RGP) was introduced. The scheme is contractual and based on patient lists and capitation. Every person must be on the list of one GP, and GPs are funded partly based on the size of the list (average list size is around 1500), partly on the number of services delivered. The RGP replaced a system where patients could choose to see any GP in their area of residence. In practice, however, two thirds of the population were already in a steady doctor-patient relationship, thus the change was not that fundamental. The reform's aim was to provide equal access across all parts of the country and a higher degree of effectiveness through more coordination between primary and specialist health care and between the primary care physician and emergency care services.

What are the special challenges in northern Norway? Recruiting and keeping primary care health personnel, especially physicians, is a challenge in rural areas. In the mid-1990s, northern Norway faced a crisis with more than one out of four GP positions vacant (Straume et al, 2010). Establishing a medical faculty at the University of Tromsø in 1972 helped increase physician recruitment, however, systematic postgraduate medical training in remote areas proved to be an even more efficient instrument for physician recruitment (Straume et al, 2010). It should also be noted that recruitment problems seem to be more related to the size of the community and its remoteness rather than climate. Thus rural communities in western Norway face the same challenges as northern Norway. Still, even today municipalities in northern Norway find it harder to recruit and maintain a stable capacity of GPs. The proportion of GP-specialists also tends to be lower in the North, especially Finnmark. For other types of health care personnel, however, there is little no difference between northern Norway and the rest of the country.

***Hospital services***

The foundation for the current organizational structure of the specialized health care was laid in the 1970s. Until then hospitals were planned, built and run as the result of uncoordinated local (mainly county and municipal) initiatives. In 1974 the country was divided into five health care regions. The basic idea behind this regionalization was that hospital services should be delivered in small "local" hospitals, larger and more specialized "central" hospitals, or highly specialized "regional" teaching hospital. The formal responsibility for hospital services would be devolved to the 19 county councils, with the exception of a few state owned ”national" hospitals. Counties within a region would (in theory) cooperate and coordinate their services and capacity, and each region would have a specialized teaching hospital. With minor modifications this organizational model lasted until 2002, at which time ownership of hospitals was centralized from the 19 counties to the central government which then delegate the responsibility for the provision of specialized health services to five independent regional health authorities (RHAs). The boundaries of these RHAs coincide with those of the former regions, thus preserving the principle of regionalization. In 2007 the number of RHAs was reduced to four, with the merging of RHA-East, with RHA-South into a RHA-Southeast, which now serves around 55 % of the country's population. At the other extreme is the North Norway RHA which serves less than 10 % of the population, about 470,000.

The responsibility for hospital services is now centralized to the state which owns the four RHAs. Each authority is governed by a board of trustees appointed by the Minister of Health and Care Services. One level down, the RHAs own hospitals that are organized as independent health trusts (*helseforetak*, or HF) with governing bodies (hospital trusts) appointed by the RHA. These boards have the same mix of politicians and other representatives as the regional boards. The strategic and operational governance of the health trusts is done – as on the regional level – through "task-documents" and annual enterprise meetings.

The North Norway RHA (*Helse Nord RHF*) supervised 5 subordinate hospital trusts:

Helse Finnmark HF – serving 73,400 people in 19 municipalities, with hospitals in Hammerfest and Kirkenes;

Universitetssykehuset Nord-Norge HF – serving 185,400 people in 31 municipalities, with hospitals in Tromsø, Harstad, and Narvik; as well as Longyearbyen in Svalbard.

Nordlandssykehuset HF – serving 133,900 people in 21 municipalities, with hospitals in Bodø, Lofoten, and Storkmarknes;

Helgelandssykehuset HF – serving 77,500 people in 18 municipalites, with hospitals in Moi i Rana, Mosjoen, and Sandnessjøen; and

Sykehusapotek Nord HF, which provides system-wide hospital pharmacy services.

There is a clear division of responsibilities and tasks between the state, the RHA and the local health trusts. The RHAs are regulated by a set of statutes that clearly defines as the responsibility of the RHA to "coordinate the activity and division of tasks between the local health trusts in such a way that it is appropriate and efficient" (author's translation). Furthermore the local health trusts also operate under a set of statues regulating various tasks and investment decisions.

RHAs are financed by a combination of needs-adjusted capitation and activity-based financing. The capitation model is similar to capitation models found in other tax-based countries such as other Nordic countries and the United Kingdom. Thus each RHA receives a share of the total budget for specialist health care that is based on the size of the population weighted by a needs index. Separate needs indices have been constructed for somatic care, mental health care, substance abuse, and ambulances and patient transport. To account for differences in the costs of providing services there is also a regional cost index.

Somatic care is also partly financed based on activity. The unit of payment is hospital discharges, classified by using diagnosis-related groups (DRGs). The share of activity-based financing is 40 %. The system of partly activity-based funding dates back to 1997, when hospitals were owned and run by counties. The share of activity-based funding ranges from 30 % to 60%.

RHAs are free to choose their own way of how to finance the local hospitals. In practice all RHAs choose the same type of model of a combination of needs adjusted capitation and activity- based financing. In order to cope with cross-border movement of patients there is also a system of transferring funds between local health authorities based on cross-border activity levels.

The ownership reform of 2002 transferring hospital ownership from the 19 counties to the state represented a re-centralization of ownership and a move from a model of devolution (through elected county councils) to deconcentration (through appointed boards). As noted the number of RHAs was reduced from five to four in 2007.

There are special challenges in providing hospital care in northern Norway, especially due to its remoteness and harsh climate. First, the costs of transportation are higher, both with regards to acute care transport and patient travel. This has been recognized in the development of a special "prehospital care" index that is used in the allocation of funds between RHAs. Thus with average population resource need set to 100, the index for northern Norway is calculated at 300, effectively allocating a higher share of per capita income to that RHA. Secondly, the cost of providing hospital services is also deemed to be higher in the North. Thus a regional cost index used in the allocation of resources to specialist health care assumes a level of costs close to 10% higher than the national average. Thirdly climate has been found to be correlated with the use of hospital services. Thus in the needs adjusted capitation model "climate and latitude" is a specific criteria, effectively allocating a larger share of resources to northern Norway.

***Public health***

There are two main goals for public health in Norway: contributing to more healthy life years in the population and at the same time reducing social inequalities in health. Health promotion and disease prevention are the responsibilities of the municipalities, delivered through school health services, health centres and child health care. However, there has also been a restructuring of the central health administration in order to strengthen the national focus on public health.

Most notably the National Institute for Public Health (*Nasjonalt folkehelseinstitutt*) has the responsibility for monitoring the development in population health, as well as translating new knowledge. The Directorate of Health (*Helsedirektoratet*) has as one of its tasks to ensure that social and health issues exert a strong influence on the general public's choice of lifestyle and behaviour, with importance given to health care and social issues in connection with political decisions in all sectors of society (Johnsen, 2006). The global concern with the increase in chronic diseases such as diabetes, cancer, chronic lung disease and dementia is also recognized in Norway. Despite its excellent health status indicators, there is nevertheless concern within Norwegian society that improvements in health seems to be slower than in other highly developed countries.

The health disparities between north and south in Norway are much less pronounced than in other circumpolar countries, although the northern counties tend score lower on social indicators such as the proportion of the population on sickness leave and disability pensions, and the general level for educational attainment. Based on analyses of the relationship between health care use and a variety of social indicators, a report for the Ministry of Health and Care Services calculated need-adjustment indexes for northern Norway. With the national average set at 100, the indices for somatic care, mental health care and drug and substance abuse treatment in northern Norway in 2007 were determined to be 116.5, 99, and 103.5 respectively (Helse- og omsorgsdepartementet, 2008).

With both prevention and promotion devolved to municipalities these differences do not trigger any centrally initiated public health policy aimed at these areas. However, the distribution of funds among different regions in Norway does take such differences into account, and thus in theory provides better access to services for the population in the northern counties.

### 6.4 Patient Pathways – the Coordination Reform

GPs act as gate-keepers in Norway. Thus only GPs are able to refer patients to specialized health care. Once the GP has approved that the patient can see a specialist there is a maximum waiting time of 30 working days within which the specialized health authority must either provide an appointment with a specialist or another form of feedback to the patient. Once the patient has seen a specialist he/she will receive the "right to health care" with a specific maximum waiting time, or placed on a less specific waiting list with only an indication of how long wait for treatment would take.

Once inside the hospital patients with complex and lasting disorders have a right to an "individual plan". The main aim of the individual plan is to facilitate coordination between specialized and primary health care and between health care and social services. Since primary care is the responsibility of the municipalities and specialized health care the responsibility of the RHAs, this requires the coordination of care between two levels of government and between two different service types within the municipalities.

Once the patient is fully treated at the hospital the municipality is responsible for further health care, often in the form of long-term care. Up until 2011 hospitals are allowed to charge municipalities for hospital costs from the 10th day after the hospital has termed the patient "fit for discharge". In most cases hospitals and municipalities will have (formal or informal) agreements on how to manage these situations should be handled. Municipal payment for hospital services is rare. However, starting in 2012, municipal payment for patients "fit for discharge" will be mandatory.

It has been acknowledged for a long time that coordination between primary and specialized health care is inefficient. Thus from 2012 a "coordination reform" will be gradually implemented with the aim of providing more timely, less resource consuming and higher quality services. The main points of the reform may be summarized as:

Strengthening the responsibility of the municipalities for the provision of primary health care services;

Making "contracts" (i.e. agreements) between municipalities and local health authorities mandatory;

Requiring municipalities to finance a portion (20 % for medical patients) of hospital treatment, and to pay in full for patients remaining in hospital after treatment is completed.

### 6.5 Sami Health Services

The majority of the Sami population in Norway lives in the northern part of the country. Among the Nordic countries, the Norwegian government has a clearly articulated policy to improve health care access and quality for the Sami population (Sosial- og helsedepartementet, 2001). Some research indicates that the Sami have a lower level of satisfaction with primary health care services, and that this is particularly related to the language skills and cultural knowledge of the GPs (Nystad et al, 2008). In addition, the Sami population tend to be concentrated in municipalities with a higher turnover of health care personnel and use of temporary personnel**.** On the other hand, a review of health expenditures in those municipalities with a high Sami population found that the overall public hospital expenditure in Sami municipalities was above the national average and equivalent to corresponding municipalities in the same geographical area. However, there was considerable variation among the Sami municipalities (Gaski et al, 2011). A survey conducted among Sami and non-Sami youths in the three northern counties found that they used health services with equal frequency. However, there were culture-specific factors which influenced the help-seeking process in indigenous youth, with some factors acting as barriers against health service use while other factors increased the probability of health service use (Turi et al, 2009).

### 6.6 Human Resources Management

**Table 6.1** compares the rate of employed health care personnel in the three northern counties with Norway nationally in two 5-year periods. There is little difference between north and south within Norway. Compared to other Nordic countries Norway has more health personnel per capita, reflecting also the higher level of expenses in Norway (**Table 1.9**).

Municipalities and RHAs are not free to establish positions for physician. GPs are allocated among municipalities according to the size and composition of population. Specialist positions are allocated according to yearly quotas to the RHAs by the Ministry of Health and Care Services. Within the RHAs these positions can be allocated freely between local health trusts. There is established a national council that supervises the allocation of physician positions as well as the specialist education of physicians. This council is part of the Directorate of Health.

To meet the challenge of recruitment of physicians to northern remote areas in Norway, the medical school at the University of Tromsø was established in 1972, and it has proven successful. Given a model of allocation of physicians that is quite centralized, recruitment problems in northern Norway is likely less serious than if the market for physicians is unregulated. Physician wages are somewhat higher in hospitals in northern Norway, but nor for other types of health care personnel, according to the "calculation committee" for specialist services (Beregningsutvalget for spesialisthelsetjenesten, 2011).

### 6.6 Conclusions

There are no formal differences in the governance, financing or delivery of health care between the southern and northern parts of Norway. There are, however, challenges related to remoteness, climate and an indigenous population that is reflected in the allocation of resources and personnel. The general impression is that access and utilization of services are as high in northern Norway as in the rest of the country, and that this also is the case for the Sami population.

**Table 6.1.** Number and rate (per 100,000) of selected health professionals in Norway and the three northern counties.

| Health professional | 2000-04 mean Norway Number | Rate | Nordland Number | Rate | Troms Number | Rate | Finnmark Number | Rate | 2005-09 mean Norway Number | Rate | Nordland Number | Rate | Troms Number | Rate | Finnmark Number | Rate |
|---|---|---|---|---|---|---|---|---|---|---|---|---|---|---|---|---|
| Physician (general) | 7585 | 167 | 432 | 182 | 426 | 280 | 179 | 243 | 9366 | 199 | 490 | 208 | 517 | 335 | 186 | 256 |
| Specialists | 8716 | 192 | 301 | 127 | 394 | 259 | 68 | 93 | 10388 | 220 | 394 | 167 | 505 | 327 | 99 | 136 |
| *Total physicians* | *16301* | *359* | *733* | *309* | *820* | *539* | *247* | *336* | *19753* | *419* | *884* | *375* | *1023* | *662* | *285* | *393* |
| Nurse | 69386 | 1528 | 3393 | 1428 | 2699 | 1775 | 1041 | 1414 | 82411 | 1747 | 4128 | 1751 | 3613 | 2339 | 1164 | 1602 |
| Public health nurse | 3083 | 68 | 174 | 73 | 152 | 100 | 60 | 81 | 3543 | 75 | 197 | 84 | 174 | 112 | 60 | 83 |
| | | *159* | | *150* | | *187* | | *149* | | *182* | | *183* | | | | *168* |
| *Total nurses* | *72469* | *6* | *3567* | *2* | *2851* | *5* | *1100* | *5* | *85954* | *2* | *4326* | *5* | *3787* | *2451* | *1225* | *5* |
| Midwife | 2422 | 53 | 133 | 56 | 112 | 74 | 47 | 63 | 2649 | 56 | 138 | 58 | 131 | 85 | 54 | 74 |
| Auxiliary nurse/ care worker | 78647 | 1732 | 5144 | 2165 | 2992 | 1967 | 1233 | 1675 | 95515 | 2024 | 5573 | 2364 | 3292 | 2131 | 1475 | 2029 |
| Dentist | 3852 | 85 | 165 | 70 | 136 | 89 | 53 | 71 | 4205 | 89 | 195 | 83 | 155 | 100 | 55 | 76 |
| Occupational/ physical therapist | 10258 | 226 | 436 | 184 | 413 | 272 | 138 | 188 | 13632 | 289 | 562 | 238 | 575 | 372 | 175 | 241 |
| Pharmacist | 2665 | 59 | 83 | 35 | 94 | 62 | 27 | 36 | 3504 | 74 | 115 | 49 | 115 | 74 | 35 | 48 |
| Psychologist | 4069 | 90 | 128 | 54 | 145 | 95 | 32 | 43 | 5584 | 118 | 159 | 68 | 196 | 127 | 42 | 58 |

Source: Statistics Norway. Statbank Tables 3448,3491,7936,7939.
Note: Number refers to employed persons with health professional education; regional data refers to employed persons working in the region. Regional data for 2008 and 2009 refer to individuals aged 15-74; all others refer to individuals aged 16-66.

### CHAPTER 7.
### NORTHERN FINLAND

Johanna Lammintakanen and Juha Kinnunen

### 7.1 Introduction

Finland is the fifth largest country in Western Europe (338,424 km$^2$) with a population of 5.4 million. It is a parliamentary republic. Since 1995 it has been a member of the European Union. Finland has two official languages – Finnish and Swedish – and citizens have a right to receive services using their own language. The Finnish population remains ethnically homogenous, and the number of permanent foreigners resident in the country is less than 3% of the total population. The precise population of indigenous Sami in Finland is not known (see Chapter 1). Sámi people are the only indigenous population in European Union having their own history, language, culture, livelihoods and identity. The criteria for Sami status is determined by an Act of the Sami Parliament. In northern Finland the Sami are concentrated in several municipalities in Lapland (Enontekiö, Inari, Utsjoki and the northern part of Sodankylä). They have the constitutional right to use their own language.

The Finnish public administration system consists of several levels: state, province (*lääni*), region (*maakunta*) and municipality (*kunta*). The political responsibility of health care at the national level rests with the Ministry of Social Affairs and Health (MSAH, in Finnish: *Sosiaali- ja terveyministeriö*, STM). MSAH defines general policy guidelines and steers the health care system at the national level. However, the central government does not regulate or direct municipal health service provision in a detailed way. The provincial authorities are part of the central government's executive branch, and have no legislative or taxation powers and are not directly elected. As of 2010, they have been replaced by the Regional State Administrative Agencies (RSAA; in Finnish: *Aluehallintovirasto*, AVI). The former Lapin lääni is now the Lapin AVI (Lapland RSAA) and Oulun lääni is now Pohjois-Suomen AVI (Northern Finland RSAA). For the purpose of this report, northern Finland is defined as consisting of these two RSAA. The Lapland RSAA has the same territory as the Lapland region, whereas the Northern Finland RSAA comprises the Northern Ostrobothnia (Pohjois-Pohjanmaa) and Kainuu regions. The RSAA and regions have no direct role in either financing or delivery of health care, with the exception of Kainuu, where a pilot scheme is in place (see below).

Local democracy is very strong in Finland. Municipalities (n=336 in 2011) are self-governing local public authorities that are responsible for providing basic social and health services and primary education to their residents. Municipalities have the right to levy taxes in order to finance these services.

Municipalities supplement their own revenues with central government subsidies and user-fees (Vuorenkoski, 2008; Teperi et al., 2009; Suomen Kuntaliitto, 2011).

In Finland, the size of municipalities varies from several hundred inhabitants to over half a million, with a median of just under 6,000 inhabitants. There are 64 municipalities in northern Finland, with population ranging from 1,000 to 140,000 inhabitants. The total population in northern Finland is almost 660,000, about 12% of the total population of the country. The small size of many municipalities often creates an imbalance between their extensive responsibilities and their capacity to provide health services. There are also demographic differences among municipalities that result in variation in health service needs, especially in the northern and eastern parts of Finland. For example, the proportion of individuals aged 65 and over is 15% in Northern Ostrobothnia and 21% in Kainuu region (Suomen Kuntaliitto 2011). Since there is no minimum coverage standard for municipalities and in resource allocation across the municipalities, there are large differences in service volumes (Teperi et al. 2009, Vuorenkoski, 2008).

Every Finnish resident has the right to health services regardless of ability to pay or place of residence. Principles of universal coverage, comprehensive range of services, public finance and service provision, equity and fair distribution of services have been typical of the Finnish system. The Finnish system is thus similar to other Nordic health care systems (Magnussen et al. 2009, Klavus et al. 2011).

The Finnish health care system is highly decentralized. The responsibility of service provision is spread among many municipalities, multiple service providers and scattered financial systems (Klavus et al., 2011). Kokko (2009) concludes that "one of the main lessons from the Finnish experience could be that decentralization can lead to loss of coherent health policy making". Therefore, MSAH has recently tightened the regulative steering of municipalities (Vuorenkoski 2008).

In terms of research and development, a network of social welfare centres of expertise covers the whole country. The centre for northern Finland (in Finnish: *Pohjois-Suomen sosiaalialan osaamiskeskus,* POSKE) has the responsibility for developing health and social welfare services for northern Finland, especially for Sami people in co-operation with Sami organizations, reflecting their experiences, cultural features and traditional livelihoods (POSKE, 2011).

During the last ten years broader public and municipal administration reforms have been undertaken which affected the arrangements in health care. The Kainuu experiment is an example of current trends to restructure local government and services. It is anticipated that municipal reform will result in mergers, reducing the total number of municipalities across the country, including the North (Kokko, 2009; Klavus et al., 2011).

The organization of health care and social services in Kainuu region differs from other parts of Finland. In 2005, the Joint Authority of Kainuu Region (*Kainuun maakunta-kuntayhtymä*) was established. Kainuu became the first and only self-governing region on mainland Finland, with an elected Regional Council as highest decision-making body. The Joint Authority is responsible for providing all social and health care services to all the inhabitants of the region, consolidating municipal services at the regional level. Still, there is a health centre in every municipality and emergency services are also available. The Joint Authority is also responsible for regional planning and development. The experiment ends in 2012 (Joint Authority of Kainuu, 2011), but will quite probably continue as a permanent structure.

The Finnish system integrates the Beveridge (general taxation) and Bismarck (national health insurance NHI) models. A mandatory health insurance system was introduced in the 1960s (Kokko, 2009; Klavus et al., 2011). NHI covers all permanent residents. It is funded by the state, employees and employers through income-based insurance fees collected with taxation (Vuorenkoski, 2008). NHI is part of the Finnish social security scheme and it provides partial reimbursement of: (1) private physician fees; (2) costs of examinations and treatments prescribed by the private physician; (3) fees of private dentists and costs of examinations; (4) medication costs; and (5) illness-related transportation costs. NHI also provides sickness allowances and partial sickness allowances. NHI does not have any defined benefit package, but it covers a certain amount of all treatments that a physician has deemed necessary for treating a disease, pregnancy or childbirth, but excludes non-medically necessary services such as cosmetic surgery (Vuorenkoski, 2008). NHI is administered by the Social Insurance Institution (*Kansaneläkelaitos*, KELA).

Historically, the service provider and purchaser has been the same - a municipality. However, municipalities have increasingly applied purchaser-provider models in organizing their health care services. The idea is to adopt more market-orientated models into traditional public administration systems to increase efficiency and effectiveness. Municipalities have separated the political and technocratic/administrative bodies. These models vary among municipalities, but typically the politicians are the purchasers (supported by professional expert teams) and they allocate the resources for different services in the municipal budgets. Either public or private providers take care of the service provision (Tynkkynen 2009).

### 7.2 Organizational Structure and Financing

Tax financing comes from two different systems: state taxation and municipal taxation. The main share of state taxation comes from value-added tax and progressive gross income tax. Other sources include corporate tax, capital income tax, alcohol tax, energy tax and car tax. The state participates in health care financing via state subsidies. On average one-third of municipal social and health care budgets came from state subsidies. The calculation of social and health care state subsidies is based on the population in the municipality, age structure, unemployment rate, income level, geographical remoteness and morbidity burden. Furthermore, the municipalities can receive extra state subsidy if they are sparsely populated (less than 2 inhabitants/km[2)], located on islands and archipelagos, and in the designated Sami region (consisting of four municipalities). They also receive funding from the central government for social and health care development projects (Vuorenkoski, 2008).

Since 2002, the Finnish Parliament has granted a separate funding in the state budget for providing social and health services in the Sami language. This funding can be used for paying subsidies through the Sami Parliament to the municipalities in the Sámi region (Magga, 2010).

Municipal taxation consists of municipal income tax, real estate tax and share of revenues from corporate tax. Municipalities decide every year the taxation rate, which creates variation among the municipalities.

User fees were introduced in 1993 for curative out-patient services in health centres. Before that time these services were free of charge. Inpatient hospital care has been charged previously. Due to concerns about the impact of user fees, especially on lower income groups, an annual ceiling for health care costs was implemented in 2000. Furthermore, legislation and governmental decree define the maximum fees which municipalities can charge for health services and also the services that must be provided free of charge, such as maternity and child health clinic visits, immunizations, and the treatment of some communicable diseases (Vuorenkoski, 2008).

In the last two decades, several Finnish and international experts, in their assessments of Finnish health care, have pointed out the inefficiency and dilemmas related to cost shifting caused by the fragmented financing system and the burden placed on exceptionally small municipalities acting as autonomous pooling funds (Klavus et al., 2009).

In 2009, the share of public financing was 75% while private financing was 25%. The public funders are municipalities, central government and KELA. The private funders are households, employers, relief funds, private insurance companies and non-profit organizations (THL 2011). In 2009, the total health expenditure was €15.7 billion (9.2 % of GDP). The per capita expenditure was 2936 Euros. Two thirds of the total health expenditure went to specialized health care (€5.2 billion), primary health care (€2.8 billion) and the

**Table 7.1.** Net municipal health care expenditures, per capita, 2000-04 and 2005-09.

| | 2000-04 mean | | | 2005-09 mean | | |
|---|---|---|---|---|---|---|
| | Whole country | Northern Finland | Lapland | Whole country | Northern Finland | Lapland |
| *Per capita (euros)* | | | | | | |
| Health and social sector | 2075 | 2005 | 2129 | 2741 | 2684 | 2853 |
| Health sector | 1079 | 1086 | 1155 | 1426 | 1421 | 1553 |
| Primary health care | 419 | 262 | 491 | 551 | 572 | 643 |
| Specialized health care | 650 | 611 | 652 | 863 | 832 | 900 |
| *Ratio region/country* | | | | | | |
| Health and social sector | 1.00 | 0.97 | 1.03 | 1.00 | 0.98 | 1.04 |
| Health sector | 1.00 | 1.01 | 1.07 | 1.00 | 1.00 | 1.09 |
| Primary health care | 1.00 | 0.63 | 1.17 | 1.00 | 1.04 | 1.17 |
| Specialized health care | 1.00 | 0.94 | 1.00 | 1.00 | 0.96 | 1.04 |

Source: THL. SOTKAnet.

consumption of pharmaceuticals and other medical non-durables in out- patient care (€2.2 billion) (THL, 2011).

In northern Finland, the net expenditures in secondary health care were € 933 - 960 per inhabitant (compared to a national mean of €954), while the net expenditures in primary health care were €617 – 746 per inhabitant (€600 nationally) in 2009. The use of private health services amounted to 0.4-0.5 visits to private physician per inhabitant in the North, less than in the whole country (0.7 visits per inhabitant).

### 7.3 Delivery of Health Services

***Primary care***

Primary health care services have been offered through health centres since 1972 when the *Primary Health Care Act* first obligated the municipalities to run health centres. According to law, every municipality must have a health centre providing primary care services. The services covered in health centres cover a broad range of preventive and primary care including medical care, dental health care, maternity and child health clinics, school and student health care, rehabilitation and physical examinations. A unique feature of the Finnish system is the GP-led health centre, which offers both long-term care for the elderly and short term care such as post hospitalization after major operations or severe acute illness. However, the law does not regulate in details how to provide these services, so municipalities have implemented different approaches. They can operate health centres of their own or jointly with other municipalities. They can also purchase primary health services from private providers. There are national guidelines for some primary care services, such as screening, immunizations and maternal health care (Vuorenkoski, 2008; Kokko, 2009).

In 2011 a new health care act merging two previous laws on primary health care and specialized medical care came into force. The aims of this new act are to integrate primary and secondary health care in order to increase efficiency, effectiveness and quality. The act emphasizes the role of primary health care. Furthermore, the act gives patients more freedom of choice between the service providers. (Vartiainen, 2010).

In northern Finland there are altogether 39 health centres operated by a municipality or federation of municipalities. The availability of services varies across the health centres although the so-called care guarantee defines statutory the time periods within which non-emergency assistance or treatment has to be provided. Furthermore, in order to decrease variation among service providers national guidelines for some services have been established, such as screening, maternal health care, elderly care, health promotion, substance abuse and school health services.

Modern information technology solutions have been developed to ensure service provision, especially in Lapland. The aim is to implement a virtual social and health care centre, which delivers increasingly more services electronically, such as online professional and client consultations, video-conferencing, and a system for making appointments on the Internet. (Lapin sairaanhoitopiri, 2011; Liimatta & Paananen, 2007).

### *Hospital services*

Features of both decentralisation and centralisation can be found in the Finnish system of specialized care services. Altogether 20 hospital districts (*sairaanhoitopiiri*) are responsible for providing municipal secondary health care. Each of them has several hospitals including a central hospital. Every municipality has to be a member of one hospital district and the member municipalities finance and manage the district. Within the territories of the two northern RSAA are four hospital districts (HD): Pohjois-Pohjanmaan HD, which manages the Oulu University Hospital and hospitals in Oulainen and Ylivieska; Lapin HD, with a hospital in Rovaniemi; Lähsi-Pohjan HD, with a hospital in Tornio; and Kainuun HD, with a hospital in Kajaani.

Hospital districts provide specialized outpatient care, inpatient care and day surgery. Many services are decentralized, i.e. every hospital district provides them. The patient needs a referral either from a GP or private physician in order to be treated in specialized care hospitals. In emergency situation, a referral is not needed (Vuorenkoski 2008).

Some features of centralisation are seen in tertiary care. Five of the central hospitals are university teaching hospitals offering more demanding treatments and tertiary care. They are located in Helsinki, Tampere, Kuopio, Turku, and Oulu. The whole of northern Finland is served by the Oulu University Hospital. University hospitals have centralised some service provision, for example, rare conditions are treated in only one or two tertiary care centres in the country. All are involved in education of health professional students and postgraduate trainees. The state owns two psychiatric hospitals (Vuorenkoski, 2008; Vartiainen, 2010).

The number of hospital districts is currently being reconsidered, with more division of labour among the districts. From the clients' perspective this could result in longer distances to access some services, such as emergency care. Another development is cross-border co-operation in the North where the borders of the three Nordic countries converge, and distances to needed services for some communities may be closer across the border in another country. A project in the Teno River Valley enables the inhabitants of Utsjoki to seek care use a variety of health services in Karasjok and other centres in Norway. The project aims to improve access to care and reduce regional disparities. Because the region is in the Sami heartland, the arrangement is intended also to improve health care for the Sami (MSAH 2010b). Despite shorter driving time, Finnish patients rarely use health services across the border, whereas Swedish patients use Finnish facilities far more frequently (Vuori et al., 2010).

### *Public health*

The main focus of Finnish health care policy for decades has been health promotion, which is carried out at both national and municipal levels. In addition, many non-governmental organizations participate in health promotion. MSAH has the responsibility for promoting public health such as tobacco and alcohol control, and environmental health protection. The *Health 2015* public health co-operation program forms the basis for Finnish health policy, with specific targets and health goals (Vuorenkoski, 2008).

In the municipalities, public health is the responsibility of the health centres. In fact, primary care in Finland is understood to encompass also public health. Therefore, maternal and child health as well as school health are among the most important services provided in health centres. Furthermore, municipalities provide free immunizations for residents according to the national schedule. They are also obliged to provide breast cancer screening for women aged 50-69 at two-year intervals, cervical cancer screening for women aged 30-60 at five-year intervals, and family planning services and control of sexually transmitted diseases (Vuorenkoski, 2008).

Non-governmental organizations have a strong role in health promotion in Finland. The Finnish Centre for Health Promotion has 125 member organizations and it is funded by the Slot Machine Association (*Raha-automaattiyhdistys*, RAY) and MSAH. For example, the NGO SamiSoster has been actively involved in providing social and health care services for Sami people (Magga, 2010). The most successful and internationally known public health campaign in Finland is the North Karelia Project which began in 1972, which has achieved the reduction in the incidence of coronary artery disease and the prevalence of its risk factors in the population (Vartiainen et al, 2010).

In sum, the health of the population has improved significantly over the past decades. However, disparities continue to exist between men and women, among socio-economic groups, and among people living different parts of the country (Martelin et al, 2006). However, the north-south gap in health status observed in some circumpolar countries is much less evident in Finland and the Nordic countries (see Chapter 1, **Fig.1.6** and **Fig.1.7**).

### 7.4 Human Resources Management

Human resources in Finnish health care are multi-professional. A typical feature for primary health care services has been the teamwork between doctors, nurses and other health professionals. For the most part, they are employed by municipally-operated health services. However, the share of private providers has increased during recent years. Finland is facing similar problems as other Western countries, with aging employees and severe recruiting problems for certain professionals, especially physicians. The challenge is more acute in Lapland (**Table 1.9**), which experiences a rate of physicians about 80% of the national norm. Finnish primary health care may face a major crisis if the health centres fail to attract enough younger health professionals (Vuorenkoski, 2008; Teperi et al., 2009; Kokko, 2009). The vacancy rate of physicians at municipal health centres across the country is 6%, compared to 16% in Kainuu, 8% in Lapland, and 6% in Northern Ostrobothnia.

A new feature in Finnish health care system is private firms hiring young and relatively inexperienced physicians and other health professionals and then contracting them out to municipalities at inflated prices. This practice also affects the continuity of care, as these contract physicians work in different posts for only a few days at a time, leaving much of the administrative and other responsibilities to the permanent staff (Vartiainen, 2010).

Nurses enjoy a relatively autonomous position in Finnish health care. In health centres, for example, they have their own consulting hours. Their role in acute care and assessing new patients (triage) has grown. Public health nurses play an important role in health promotion. Nurses with extra training working in health centres have been given limited rights to prescribe medications for patients with specific medical conditions if they have physician authorization (Teperi et al. 2009; MSAH 2010a).

In Finland, the educational system produces high-quality health care professionals. However, health human resource management is relatively underdeveloped. Senior management positions tend to be occupied by senior clinicians with little management skills (Teperi et al., 2009). A survey of primary care managers showed that different regions have adopted different strategies to cope with the shrinking pool of new recruits. In southern region, managers tend to look abroad to find new employees, while in the North, managers put effort into retaining the employees in the organization with different human resources management practices (Lammintakanen et al., 2010).

### 7.5 Conclusions

Finnish health care has been reformed continuously for decades in order to improve effectiveness, enhance quality, raise the level of management skills of health care professionals and increase patient choice (Vartiainen, 2010). There has been, however, problems in coordination and implementation of these reforms, which has led to the separate development of public and private services (Teperi et al 2009). Furthermore, the regional differences in terms of service provision and availability are quite remarkable. Cross-border co-operation in northern Finland with Sweden and Norway has the potential to improve access to care, particularly among the Sami population in the area.

The Finnish example has shown that a decentralised health care system has not been able to meet the citizen's needs equally. Therefore, the direction of reforms has been towards the creation of bigger units by integrating social and health services within social and health care districts, and by merging municipalities to produce catchment areas of at least 20,000 people.

While municipalities retain their responsibility for health service provision, their role will be affected by different applications of purchaser-provider models and other new public management practices. The increasing number of private service providers to date has been observed mainly in large urban centres and less so in small northern communities.

The new health care act is designed to increase clients' freedom of choice, but may have the effect of reducing equity for those living in northern, remote areas, since they may not have the opportunities for choosing service providers. The development of e-health applications in health care may contribute to better access to medical expertise not available physically.

Improvement in human resource management and planning is needed to sustain the health care system. Unlike in other Nordic countries, Finland cannot rely on importing health professionals from its neighbours due to language barriers.

### CHAPTER 8.
### CROSS-CUTTING ISSUES

Gregory Marchildon, Jon Magnussen, Kue Young,
Peter Bjerregaard, Susan Chatwood, Kathryn Anderson

### 8.1 Introduction

The purpose of this section is to illustrate, with a very broad brush, the similarities and differences among circumpolar health systems along several dimensions.

The first is financing where we examine public-private responsibilities for financing and present a basic typology of circumpolar financing systems. The second is the degree of decentralization from a political perspective as well as health system governance point of view. The third dimension examines the types of health human resource models in terms of the administration and delivery of primary health care services given the great variety of approaches in the circumpolar world.

Given the differences in indigenous people's share of the various regional populations, different approaches have emerged in how regional governments and health authorities provide services to indigenous people. The role of public health within the health care system also differs across circumpolar jurisdictions, although all proclaim its importance. Finally, different regions have designed, tested and implemented innovative technologies, notably in eHealth, that offer potential solutions to the delivery of health services in widely scattered, remote communities.

### 8.2 Financing

All circumpolar health systems are financed predominantly through public funding though the proportion of public to private varies from roughly 75% (northern Finland and Yukon) to over 95% (Greenland and Nunavut). The one exception is Alaska where less than 50% of financing sources are public, a product of the evolution of the health system in the United States that was built on employment-based private insurance (Hacker, 2002).

In terms of financing models, the majority of circumpolar health systems fall within the Beveridge model – integrated, single-payer health care systems financed by general taxation with minimal or no user charges. Northern Canada, Greenland, Iceland, northern Norway and northern Sweden lie firmly within the Beveridge model. Northern Finland combines social health insurance (the Bismarck model) with general taxation to produce a hybrid system although it is closer to the Beveridge model in terms of its integrated and single-payer features (Kutzin, 2001).

In the case of northern Russia, the health system is mainly financed by an obligatory medical insurance payment by companies, supplemented by tax-based subsidies. This earmarked tax provides all Russian citizens with statutory (social) health insurance. However, the legacy of an integrated, socialized health system combined with the constitutional right of Russian citizens to obtain state health care and medical assistance free of charge and the absence of any employee contributions to the earmarked tax, suggest that the Russian system may be closer to the Beveridge rather than the Bismarck model.

While private health insurance remains an important part of financing in Alaska, the important role of public health coverage through Medicare, Medicaid, the Indian Health Service and the military produces a truly hybrid system that mixes private, general tax and social health insurance sources of financing. This fragmentation of financing, and ultimately governance, administration and service delivery, has created the most complex health system in the circumpolar world.

Those jurisdictions that have special central government transfers, or financing arrangements, which take into consideration climate and remoteness, either directly or indirectly, seem to allow for much higher than average (relative to the reference country) per capita expenditures in circumpolar regions. Norway provides the clearest example of an explicit provision where both climate and latitude are specific criteria in determining central government transfers for specialist services. Finland allocates higher state subsidies to municipalities that have population density below a certain level. The federal government of Canada provides an example of an implicit provision through its higher than average transfers to the three northern territorial governments (relative to the provincial governments).

### 8.3 Decentralization

We discern two types of decentralization. The first is at the highest, political level, in order to separate those circumpolar entities that are self-governing and therefore have the legal and political capacity to administer and reshape their own health systems, and those regions that are an integrated part of a larger, national system, largely shaped by laws and political processes located further south. The second type of decentralization is more specific to the actual workings of circumpolar health systems and examines where such systems sit in the continuum between highly centralized and highly decentralized in terms of administrative decision-making.

When it comes to high-level political decentralization, with the exception of Iceland, no single circumpolar region as defined in Chapter 1 enjoys complete sovereignty. In its relationship with Denmark, Iceland evolved from a sub-state with home rule in 1918 to a fully independent republic in 1944. Greenland is on a similar trajectory having obtained home rule in 1979 to self-rule in 2009 while Denmark remains in charge of foreign affairs, defense and fiscal policy including funding transfers and currency. Similarly, the Faroe Islands achieved home-rule in 1948. In the three North Atlantic island states, health system administration is relatively centralized within the ministries of health in each jurisdiction.

The United States, Canada and Russia are continental federations covering vast territories. All three countries have constitutions that divide powers and responsibilities between the central government and their respective substates variously known as states, provinces, and, in the case of Russia, republics, oblasts, krays and autonomous okrugs.

Alaska emerged from its territorial status to a full-fledged state in 1959. As such, it has control over the administration of Medicaid. Beyond this, however, the health system is highly fragmented with administrative decision-making divided among federal, state and Alaska Native authorities as well as numerous private insurers and health care organizations.

Although the three territories in northern Canada do not enjoy the constitutional status of provinces, in practice they exercise self-governing responsibilities that parallel provincial authorities and responsibilities, including administering their own single-payer primary care and hospital services. The territorial governments are also responsible for coordinating most other health services including long-term care. In the Northwest Territories, the government has further delegated its responsibilities to publicly-appointed regional health authorities.

In the multi-layered Russian Federation where federal substates (subjects) have differing powers and responsibilities, varying degrees of political and administrative control are exercised over the health systems by the numerous substates in Siberia and northwestern European Russia.

In contrast, to the current and former Danish dependencies and the continental federations, health care in the northern regions of the Nordic countries are governed and administered as part of more integrated national systems. The basic laws, financing systems, benefit packages and reforms are, for the most part, determined by national laws made in national legislatures. However, there is considerable administrative delegation at the local level for the administration of health services. Regional governments and municipalities in all three Nordic countries are responsible for primary health care and social care services including long-term care. In Sweden county governments are also responsible for administering hospital services while Norway and Finland have witnessed the emergence of hospital districts which centralized the administration of secondary and tertiary care.

### 8.4 Human Resources

In remote circumpolar communities, the first point of contact with a health system is critical. The nature of that primary care contact has an important (though not determinative) impact on health outcomes including mortality. This impact is determined by a number of factors, including quality and timeliness of basic treatment, the prevalence of prevention and health promotion services, and the availability and effectiveness of referrals for more specialized treatment and care.

Every circumpolar health system has a primary care infrastructure and related human resources. There appear to be two main approaches. The first is a general practitioner model that dominates in the Nordic countries and the current and former Danish dependencies although village health workers and nurses are the first point of contact in the smallest communities in Greenland.

The second is the substitutive model where health practitioners other than doctors provide the majority of primary health care services. This is true for northern Canada where community health nurses play a central role in remote communities, in northern Russia where feldshers provide almost all primary care services in the rural and remote areas, and in the remote communities of Alaska where community health aides constitute the backbone of primary care.

In the larger urban centres in all circumpolar regions, primary care services are provided by general practitioners irrespective of the health human resource model of delivery (although in Alaska nurse-practitioners and physician assistants are also widely deployed in cities). In addition, these models obscure other differences among circumpolar regions. In some regions such as northern Canada, Greenland and Norway, the first contact acts as gatekeeper in terms of referral to specialists, while in Iceland, patients have the right to access specialists directly. In some circumpolar regions such as northern Canada, Greenland, Russia, Finland, the general practitioners are employed directly by the state or substate and work within a public infrastructure, while in other regions such as Norway, these providers are private practitioners obtaining some or all their remuneration through fee for service payments. Alaska is a hybrid of the two.

Finally, in some circumpolar systems such as northern Canada, Greenland and northern Russia, services are free at the point of access. In the Nordic countries, there is a fee or co-payment for primary care.

### 8.5 Indigenous People

As northerners, indigenous people have certain health care needs that are not very different from those of non-indigenous people, to the extent that health care access and quality are affected by geography, climate and human resources. The need for culturally specific and sensitive care is often voiced by indigenous people, particularly in areas where they are a minority. The poor health outcomes and the persistent health disparities between indigenous and non-indigenous people in most regions cannot be attributed solely to deficiencies in the health care system, given the more significant role of social determinants of health that are at play.

The remote and scattered locations of indigenous people's settlements necessitate referrals and medical travel to a degree unknown to health care systems in the South. That this is a prominent health care experience of indigenous people is due to the fact that the most remote communities tend to be inhabited by indigenous people whereas the majority of non-indigenous northerners reside in larger urban centres within the North.

Given the wide variation in the size of the indigenous population and its share of the total regional population across the Arctic, a variety of health care delivery systems exist to serve indigenous people. These systems fall into several distinct patterns, which vary from a totally separate and parallel system for indigenous people exclusively, to "one system for all" with no special provision for indigenous people. How such systems develop reflects the historical relationship between indigenous people and the majority population and the nation-state, and also the nature of the national health care system.

Alaska is the only example of a separate and parallel system for indigenous people in the state, although cross-over between the two systems is possible. If an Alaska Native person is employed by a private employer that provides health-insurance, he/she has the same coverage and choices as a non-Native employee of that same company. Similarly, if an Alaska Native person is in the military or is a veteran, he/she has the same coverage and choices as a non-Native person in the same category. Alaska Natives aged 65 or older are eligible for Medicare. If they also meet income eligibility requirements they are also eligible for Medicaid. Eligible Alaska Natives can use their non-Native benefits in either the Native health care system or the applicable non-Native systems. In fact, IHS only covers about 60% of the total Native Health budget: private insurance, Medicare, Medicaid, and military benefits cover the rest. Remote clinics run by the Native System that have a particular federal designation (Community Health Clinic) may see non-Native patients. As a small exception, non-Native pregnant women carrying a Native baby may be served in the Native system until a few months after birth.

In regions where indigenous people form the overwhelming majority of the population, such as Greenland, Nunavut, and some health regions in the Northwest Territories (Beaufort-Delta for the Inuvialuit and Gwichin, and the Sahtu, Deh-Cho, and Tlicho health authorities for the Dene), the health care system that exists for all people can be considered for all intents and purposes an indigenous health care system. Under various self-government and land-claims agreements, such systems are also controlled by indigenous people.

In regions where indigenous people are the minority, such as in northern Fennoscandia and Russia, indigenous people use the same system as other citizens. Such systems may not have special provisions for indigenous clients, although in certain pockets where indigenous people are concentrated, there may be specific services catering to the unique cultural needs of indigenous people, such as services provided in the Sami language. Norway has declared Sami health care as a priority policy. In Finland, municipalities where Sami are a majority receive additional state subsidies.

Unique to Canada, many First Nations reserves in the southern provinces and also in Yukon may run some of their own services (usually in primary care, or support services), while for other services, residents use health care providers/institutions the same as general population. These communities are not part of a regionalized system, and individual communities or First Nation councils, signed agreement with the federal and territorial governments for such "transfer of control".

### 8.6 Public Health

The concept of "public health" differs slightly from country to country, especially as the boundary between "primary care" and "public health" becomes blurred in jurisdictions where such services are well integrated in terms of facilities and service providers. Different circumpolar countries and regions have produced public health plans that reflect the country or region's epidemiologic situation or social policy orientation. Greenland's *Inuuneritta* was passed as an act of parliament in 2006, thus having the force of law and not just a bureaucratic instrument, an approach that is also undertaken by other Nordic countries. Norway's *White Paper*, Sweden's *Public Health Objective Bill*, and Finland's *Health 2015 Programme* have a strong focus on reducing health inequalities and subscribe strongly to the social determinants of health model.

Public health surveillance is a core function in any system. The Nordic countries have a clear advantage over the other regions with their population registries and the ability for data linkage among various health databases (Irgens, 2000; Swedish Centre for Epidemiology, 2003). The Alaska Native Epidemiology Center of the Alaska Native Tribal Health Consortium is able to maintain a variety of registries (on cancer, heart disease, diabetes, stroke, and trauma) that go beyond the usual public health ones, primarily because of the close link between public health and clinical care, up to the tertiary level, within the same population-based organizational structure.

Some surveillance functions also occur at the international level, notably the NORDCAN database of the Association of Nordic Cancer Registries. The International Circumpolar Surveillance (ICS) on selected infectious diseases was launched as an Arctic Council endorsed project in 1999, creating a network of participating hospital and public health laboratories, with a focus on invasive bacterial diseases (Parkinson et al, 2008).

Healthy lifestyles related to nutrition, physical activity and smoking are promoted at the national, regional or local levels. Programs may be based in schools, health care settings, communities, and involve the use of mass communication. Multisectoral action plans have been developed in some countries and their implementation evaluated (Backhans and Moberg, 2008). Different approaches are used, for example, Finland has traditionally placed strong emphasis on partnerships with non-governmental organizations such as the diabetes and heart associations to develop disease-specific strategies and programs.

In addition to increasing awareness and individual behavioural change, the legislative and policy environment also need to change. In Canada, both Nunavut and the Northwest Territories have passed comprehensive *Tobacco Control Acts* which prohibit sale of tobacco products to youths under the age of 18, advertising and public display, and also smoking in public places. The European Network for Smoking Prevention periodically evaluates tobacco control policies in 31 European countries and ranked them on the basis of their efforts in six cost effective interventions. The Nordic countries have consistently ranked within the top 10, with the exception of Denmark (Joossens & Raw, 2011). However, no such evaluation has been conducted among the northern regions of these countries.

Maternal and newborn services serve as the first entry point to the public health system for most families in circumpolar regions. The Nordic countries report some of the world's best maternal and infant health outcomes which in part have been attributed to well coordinated prenatal care, birthing services and follow up, with midwives playing the key role (Expert Group on Acute Maternity Services, 2002). Maternal and child health services in Alaska is typical of much of North America (although not in northern Canada) with services provided by both public health nurses in the state system and physicians, obstetricians and pediatricians in the private sector. However, for Alaska Natives a comprehensive system does exist where such services are integrated with primary health care. This is also the system in place in northern Canada.

Equity is an explicit objective of the Nordic countries' health systems. With their well established welfare states, social inequalities are actually much less acute than other "rich countries" such as Canada and the United States. It is interesting that in the make-up of their health ministry, the agencies responsible for standards and quality in health care are generally also tasked with ensuring equity. There are two different approaches to redressing inequality – the universalistic and the targeted group approaches. Finland has adopted a universalistic policy approach: by ensuring equal access to services and benefits for all citizens regardless of their social background and geographical location, inequalities will be reduced. The drawback is that services and benefits may not actually reach all subgroups equally, and that they produce equal results (Palosuo et al, 2008).

Norway's approach to inequalities appears to have shifted over the years. In the 1990s, inequality was mainly perceived of in terms of disadvantaged, vulnerable, or marginalized groups and individuals – e.g. low income families, immigrants, mentally ill, drug addicts, and the homeless (Dahl 2002). As a follow-up to the *White Paper on Public Health* of 2003 and the subsequent national strategy to reduce social inequalities, there was a shift in focus away from targeting only the poorest groups to addressing the social gradient in the whole population (Fosse 2008).

### 8.7 Telehealth Innovations

Telehealth, or e-health, is the use of communications and information technology to support health care services when participants are physically separated. E-health applications can be especially useful in the circumpolar north as a means of overcoming the obstacles to good health care presented by harsh climate and remoteness. Circumpolar regions have been world leaders in the development of e-health initiatives since the mid-20th century.

Four elements of e-health that are particularly relevant in the circumpolar north are telemedicine, infrastructure, electronic health records, and education. Telemedicine refers to a clinical application in which diagnosis and treatment is facilitated by an expert who is in "virtual" and not physical contact with the patient. Infrastructure refers to the underlying telecommunications structure, whether that is satellite, fibre optics or microwave, and to the information technology architecture. Electronic health records are a key underlying component of effective telemedicine, because they ensure that accurate, consistent, and timely data are available at both the patient and the provider ends of the interaction. Education refers to health care personnel in remote areas learning without having to travel.

The three basic forms of telemedicine practiced in the circumpolar north are real-time interactive consultations, store-and-forward applications, and telemonitoring.

In real-time telemedicine, the patient is remote from needed expert care and may be attended by a local provider such as a community health aide, a nurse, or a general doctor. The patient and, where appropriate, the local practitioner interact with the remote expert through two-way audio/video and high-speed patient record exchange. The patient is diagnosed and potentially treated through the facilitation of the remote expert. Examples of this type of application include psychiatry, cardiology, and dermatology, all of which are practiced widely in the circumpolar north.

In store-and-forward telemedicine, images and other information are captured by the local practitioner, or perhaps by the patient directly, and sent to a central location where an expert can examine the data. In these types of applications, the patient is often not in immediate distress, and the remote analysis can be done over a period of hours or days. Teleradiology is a primary example of store-and-forward telemedicine, and it is also practiced widely in the North.

In telemonitoring applications, the patient sends data periodically to their provider via the internet or mobile phone. This data may be entered manually by the patient into a computer or cellphone, or transmitted from a medical device such as a heart monitor. Examples of telemonitoring applications in use in polar regions include blood glucose levels for diabetes care, kidney function tests in dialysis patients, and cardiac rhythms in cardiology.

Another use of ICT in circumpolar health applications is the cost-effective continuing education of remotely deployed health care workers. Such training avoids the time and travel costs associated with attending training in larger population centres. For example, videoconferencing has been used to deliver training to practicing nurses and mental health workers in Canada, Alaska, and Norway. Internet-based classes have been used to allow nursing and public health students to conduct a large portion of their education without leaving their homes in rural Alaska.

Implementing and sustaining an effective telemedicine application can be especially difficult in a circumpolar region. The medical and ICT equipment in use at the remote end must be both ultra-reliable and simple to use and maintain, given the likely level of skill available locally and the potential difficulty of sourcing spare parts. There may be also cultural concerns among indigenous peoples regarding the use of high technology and remote personnel.

The type of telemedicine applications can be offered in a given region is dictated by the available information and communications technology (ICT), infrastructure and the structure of the healthcare system itself.

Two-way interactive video and the ability to transmit high-resolution images require relatively high bandwidth, best served by fibre optic cable, while store-and-forward and telemonitoring require somewhat less bandwidth. There is sub-optimal coverage of polar regions by satellites, and they have too high a latency for quality interactive video and high resolution image transmission. Satellites are also prone to outages due to solar and other space activity. Microwave is more reliable than satellite, but it is also slower than fibre optic cable. In relatively compact countries with government support for broadband deployment, such as the Nordic countries, high bandwidth in remote areas is more easily deployed. In other settings, such as Canada, Alaska and Russia, the area involved may be too large and too remote for affordable laying of fibre or microwave, and the subsidies required may not be available from the federal or regional governments.

Beyond the network infrastructure, ICT must address data interoperability standards in order for communication of electronic health records to be feasible. This work is complex, as it requires interfacing many different legacy computer systems at both a networking level and at a data element level. Circumpolar countries with tightly integrated health care systems will likely have a simpler task than those who are highly decentralized. Their in-place computer systems may have more in common than decentralized systems, and, by virtue of their centralization, they will have an easier time mandating system changes and timetables.

Many circumpolar regions are engaged in national and international efforts to establish electronic health record standards. National/sub-national level efforts include the Canadian Institute for Health Information (CIHI, 2011c), the Icelandic Healthnet (Persephone et al, 2010), the Alaska e-Health Network (2011), and the Swedish National Strategy for e-Health (Ministry of Health and Social Affairs, 2009). International EHR standardization efforts in which circumpolar nations participate include the European Commission's ICT for Health initiative (2011) and the WHO Collaborating Centre for Telemedicine and e-Health, located at the Norwegian Centre for Integrated Care and Telemedicine (NST, 2010).

E-health applications have the potential to positively impact the quality, access, and cost of care in a remote setting, and evidence has been cited for each of these (Mitton et al, 2011). Quality of care can be improved, because expertise that could not practically be available in sparsely populated areas can be "virtually" deployed. Patient access to care is improved for similar reasons.

Cost impacts include the avoidance of both medical evacuation and hospitalization, through the timely application of expert care. Reducing the need for higher-paid health care providers in remote places also lowers costs overall. However, the cost of deploying full broadband capability to remote areas is substantial as well, and the ultimate cost-effectiveness of telemedicine in the circumpolar north is highly dependent on the utilization of the ICT infrastructure.

### CHAPTER 9.
### POLICY IMPLICATIONS AND FUTURE RESEARCH

Gregory Marchildon and Kue Young

There are three major factors that distinguish circumpolar health systems from other health systems in the world. The first is a sparse population distributed over a vast geography. The second factor is the high concentration of indigenous peoples in some of these regions, relative to the majority populations of circumpolar countries. The third factor is the cold climate and the extent to which harsh climatic conditions influences the health system, both in terms of what services are offered and the manner in which they are provided. However, as illustrated in the preceding chapters, there are significant differences among the circumpolar regions, and these are worth highlighting as they create exceptions to any general policy implications and recommendations for future research that flow from this review.

### 9.1 Health Systems Challenges

The first challenge faced by health system decision-makes is the low population density and rural and remote nature of the majority of communities in circumpolar regions, although in most regions, the majority of the population is concentrated in a few urban centres. In all cases, the population density of the circumpolar regions is considerably less than the respective circumpolar country to which they are attached. The very lowest population densities in northern Canada, northern Alaska and the northeastern regions of the Russian Federation are due to the frigid winter climate and, in the case of Greenland, the existence of an enormous ice cap that covers that vast majority of the island. In these regions, the limited economies of scale put a premium on medical transportation since hospital treatment as well as more specialized diagnoses and treatments will be provided in centres outside the majority of these small and scattered communities.

The more temperate climates of Fennoscandia, Iceland, southern Alaska, the westernmost regions of northern Russian and the Faroe Islands allow for larger populations, and in some cases, sizeable cities. In these circumpolar regions, there is more opportunity for the delivery of hospital and other specialized health services within the communities and less of a premium on medical evacuation to centres outside such regions. In the largest cities in such regions there is often a specialized teaching hospitals such as those in Tromsø (Norway), Reykjavik (Iceland), Anchorage (Alaska), Arckhangelsk (Russia) and Oulu (Finland). At first glance, it appears that the existence of such academic health centres may be a positive factor in helping address the health human resource shortages that are often the rule in circumpolar regions but more research is needed to address this connection before anything more definitive can be stated. On the other hand, their presence may also inflate the regional rates of health professionals and hospital bed capacity which may not necessarily improve access to health care in the remote communities within the regions.

As pointed out in Chapter 1, while there is no universally accepted definition of indigenous populations, most circumpolar countries have laws or policy practices that identify these groups. Based on these definitions, we can say the following: indigenous populations form a majority of the population in northern Canada, Greenland, northern Alaska, and a number of regions in Russia, and they constitute significant minorities in other Russian regions as well as in the northern regions of Norway, Sweden and Finland. Iceland and the Faroe Islands are the only exceptions.

There also seem to be two quite different health system policy approaches. In the United States and Canada, there are constitutional, legal and programmatic policies that separate indigenous populations from the rest of the population. As a consequence, the health systems in these circumpolar regions have differentiated funding and benefit streams in the public sector and, in the case of Alaska, tribally-administered health services. In contrast, there is the Nordic model of integrated financing, administration and delivery which does not distinguish between indigenous and non-indigenous populations in northern Fennoscandia and Greenland. To date, there has been no systematic effort to compare the results obtained by these two health system approaches.

While all circumpolar regions have a colder climate – often considerably colder – than other regions in their respective countries, there is considerably variability in winter temperatures among the circumpolar regions. The residents of northern Canada, the north-eastern regions of Arctic Russia and northern Alaska face much harsher winters than the residents of southern Alaska, Greenland, Iceland, the Faroe Islands and northern Fennoscandia, with the residents of north-western European Russia contending with average winter temperatures only slightly cooler.

Health facilities in the coldest circumpolar regions must be constructed in a manner that they can be used effectively in such climatic extremes. The same applies to medical transportation as patients and their respective health providers depend heavily on aircraft, winter land vehicles and boats operating on a dependable basis. This involves extra cost for circumpolar health systems and it would be useful for researchers in the future to determine the extent to which the higher health system costs in some of the most climatically extreme areas in northern Alaska, northern Canada, and northern Russia can be attributed to the extra cost of cold weather facilities and transportation relative to other circumpolar regions. With the effects of global warming already being observed in the Arctic, considerable impact on the health care system can be expected. For example, the melting of the permafrost will compromise the structural integrity of health care facilities and transportation installations.

The disease pattern may likely change also. The impact of climate change on health care delivery will need to be closely monitored in the future.

### 9.2 Health System Responses

Health systems are both labour- and skill-intensive. For their effective functioning, health systems depend on the contributions of skilled clinicians, managers and technologists organized into dozens of professions and trades. It is estimated that roughly two-thirds of public-sector health systems are made up of payments to health human resources. As the individual region chapters indicate, one of the largest challenges in all circumpolar regions is the shortage of providers.

For primary care, there have been two main responses. The first is the physician-based system of primary care that is dominant in the Nordic circumpolar regions, including Greenland, Iceland, the Faroe Islands and northern Norway, Sweden and Finland. The second is a primary care approach that depends on middle-level health providers who provide services in the physical absence of physicians. In the remote communities of northern Canada and Alaska (as opposed to the more urbanized portion of southern Alaska and the territorial capitals of northern Canada), the almost complete absence of resident physicians has resulted in a hub-and-spoke system of primary health care with nurses and community health aides in the front lines. In northern Russia, primary care in the rural and remote areas is provided by *feldshers*, who also provide similar care in rural communities in the rest of Russian Federation.

These approaches produce very different modalities of primary care, and they should be subjected to more systematic comparative research in the future. While it would be difficult, if not impossible, for the nurse-based or *feldsher*-based systems to become physician-based primary care systems in the future given demographic and human resource realities, it is the case that some physician-based systems, such as that in Greenland, are now introducing more middle-level services to substitute for the declining number of physicians in remote communities. For this reason alone, it would be useful to conduct research on systems that are highly dependent on middle-level providers for primary care in circumpolar environments.

The use of ehealth technology and telehealth to improve the quality of, and access to, health service delivery in northern regions has often been advocated Circumpolar regions have served as sites for pilot projects evaluating these technologies as substitutes for providing access to physicians and other specialized health providers and more specialized or expensive diagnostic equipment and tools. Some regions are on the verge of territory-wide implementation of systems such as electronic medical records, and their experience in improving quality and access and reducing costs, deserves close scrutiny.

Given the lack of specialized health providers and medical facilities, particularly in the most sparsely populated circumpolar regions, there will be a corresponding need for medical transportation to centres with such facilities. As a consequence, medical transportation forms a major part of the policies and cost of public healthcare systems in circumpolar regions. In this special edition, we were unable to address in any detail, much less compare systematically, the medical transportation policies and infrastructure in the circumpolar regions, due in part to the low volume and quality of studies within each of the circumpolar countries. However, due to its importance as a public policy, medical transportation should be examined carefully in future research studies on circumpolar health systems.

Holding all other factors constant, it only stands to reason that health systems in circumpolar regions should be more expensive than other regional health systems in their respective countries. Yet, as Chapter 1 demonstrates through its analysis of expenditure ratios within circumpolar countries, this is not borne out empirically. For Canada and most circumpolar regions within the Russian Federation, it is true. It is very marginal in the cases of northern Sweden and Finland – here, Norway stands out a little if only because of the special financing formula that takes into account climate and other northern features.

In the self-governing territories of Greenland and the Faroe Island, the opposite is true - their health systems actually cost less than the Danish system as a whole. The reasons for this surprising results need to be more closely examined to determine whether there are unique delivery features that provide policy lessons for all circumpolar regions or whether outcomes are simpler poorer. Although there are statistical challenges posed by the small size of the populations in question, it would be useful to calculate and compare amenable mortality rates – deaths that can be avoided through health system interventions – in circumpolar regions as the first stage in determining whether there is some correlation between these ratios and outcomes. This would help policy decision-makers in making some basic "value-for-money" assessments when deciding how to refashion their respective circumpolar health systems in the future. In particular, there are important tradeoffs to be made between building more local infrastructure and attracting more local providers and providing better and more effective medical transportation.

### Abbreviations

| | |
|---|---|
| AHRQ | Agency for Healthcare Research and Quality |
| AIAN | American Indians and Alaska Natives |
| ANTHC | Alaska Native Tribal Health Consortium |
| AO | Autonomous okrug |
| AVI | Aluehallintovirasto |
| CDC | Centers for Disease Control and Prevention |
| CHA | Community health aide |
| CHC | Community health clinic |
| CHN | Community health nurse |
| CHR | Community health representative |
| CIHI | Canadian Institute of Health Information |
| CMMS | Center for Medicare and Medicaid Services |
| DHHS | Department of Health and Human Services |
| DRG | Diagnosis-related group |
| EHR | Electronic health record |
| EMR | Electronic medical record |
| Eurostat | European Statistical Agency |
| GDP | Gross Domestic Product |
| GP | General practitioner |
| HD | Hospital district |
| HF | Helseforetak |
| HPSA | Health Professional Shortage Area |
| HRSA | Health Resources and Services Administration |
| IHS | Indian Health Service |
| KELA | Kansaneläkelaitos |
| MSAH | Ministry of Social Affairs and Health |
| NGO | Non-governmental organization |
| NHI | National health insurance |
| NIH | National Institutes of Health |
| NIHB | Non-Insured Health Benefits |
| NOMESCO | Nordic Medical Statistical Committee |
| NP | Nurse practitioner |
| NWT | Northwest Territories |
| OECD | Organisation for Economic Co-operation and Development |
| PA | Physician assistant |
| PACS | Picture archiving and communication system |
| PHCCA | Primary Health Care of the Capital Area |
| POSKE | Pohjois-Suomen sosiaalialan osaamiskeskus |
| RAY | Raha-automaattiyhdistys |
| RGP | Regular General Practitioner Scheme |
| RHA | Regional health authority |
| RN | Registered nurse |
| RSAA | Regional State Administrative Agency |
| STM | Sosiaali- ja terveyministeriö |
| UNDP | United Nations Development Programme |
| WHO | World Health Organization |
| WWAMI | Washington, Wyoming, Alaska, Montana, Idaho |

### Bibliography

ADA. (various years). *Distribution of Dentists in the United States by Region and State*. Chicago: American Dental Association.

AHA. (various years). *Trendwatch Chartbook: Trends Affecting Hospitals and Health Systems*. Washington, DC: American Hospital Association. Retrieved from http://www.aha.org/research/reports/tw/chartbook/archive.shtml

Alaska Center for Rural Health. (2006). *SORRAS II: Status of Recruitment Resources and Strategies 2005-2006*. Retrieved from http://www.hss.state.ak.us/DPH/Healthplanning/publications/assets/SORRASii06.pdf

Alaska Center for Rural Health. (2009). *Alaska Health Workforce Vacancy Study 2009*. Retrieved from http://acrh-ahec.uaa.alaska.edu/projects/pdf/2009workforce09.pdf

Alaska Department of Health and Social Services. (2007). *Alaska Health Care Data Book: Selected Measures, 2007*. Section III. Health care workforce and shortage designations. Retrieved from http://www.hss.state.ak.us/DPH/Healthplanning/publications/healthcare/assets/section3.pdf

Alaska eHealth Network. (2011). *About AeHN*. Retrieved from http://www.ak-ehealth.org/

Alaska Health Care Commission (2010). 2009 *Report*. Appendix A: Health care in Alaska. Retrieved from http://www.hss.state.ak.us/healthcommission/docs/appendix.pdf

Alaska Native Health Board. (n.d.). About ANHB. Retrieved from http://www.anhb.org/index.cfm?section=About

Alaska Native Tribal Health Consortium. (2011). *Alaska tribal health system community health aide program*. Retrieved from http://www.anthc.org/chs/chap/

Alþingi. (2007). *Lög um heilbrigðisþjónustu* [Health Services Act]. Vefútgáfa Alþingistíðinda [Web publications of Parliament]. Retrieved from http://www.althingi.is/lagas/nuna/2007040.html

Alþingi. (2008). *Lög um sjúkratryggingar* [Health Insurance Act]. Vefútgáfa Alþingistíðinda [Web publications of Parliament]. Retrieved from http://www.althingi.is/lagas/nuna/2008112.html

Ásgeirsdóttir, T.L. (2007) *Holdafar – hagfræðileg greining* [Weight condition: an economic analysis]. Reykjavik: Lýðheilsustöð [Public Health Institute of Iceland].

Anderson, K. (2010). A review of health care reform in the United States and in Alaska. *International Journal of Circumpolar Health, 69*, 424-436. Retrieved from http://www.ijch.fi/download.php?abstract_id=1054&file_nro=1

Backhans, M., Moberg, H. (2008). Sweden. In Hogstedt, C., Mobderg, H., Lundgren, B., Backhans, M. (Eds.), *Health for All? A Critical Analysis of Public Health Policies in Eight European Countries* (pp.293-331). Östersund: Swedish National Institute of Public Health. Retrieved from www.fhi.se/PageFiles/4171/R200821_Health_for_all_komp0809.pdf

Beregningsutvalget for spesialisthelsetjenesten. (2011). *Årsrapport 2011* [Annual report]. Retrieved from http://www.regjeringen.no/upload/HOD/SHA/BUSrapport2011.pdf

Bureau of Labor Statistics. (2011). *Occupational Employment Statistics*. Washington, DC. U.S. Department of Labor. Retrieved from www.bls.gov/oes/oes_arch.htm

Centers for Medicare and Medicaid Services (2011). *National health expenditures by type of service and source of funds, CY 1960-2009*. Retrieved from http://www.cms.gov/NationalHealthExpendData/02_NationalHealthAccountsHistorical.asp#TopOfPage

CIHI. (various years). *Supply, Distribution and Migration of Canadian Physicians*. Ottawa: Canadian Institute of Health Information. Retrieved from https://secure.cihi.ca/estore/productSeries.htm?pc=PCC34

CIHI. (2009). Analysis in brief: Experiences with primary health care in Canada. Retrieved from http://secure.cihi.ca/cihiweb/products/cse_phc_aib_en.pdf

CIHI. (2010). *Regulated Nurse: Canadian Trends, 2005 to 2009*. Ottawa: Canadian Institute of Health Information. Retrieved from http://secure.cihi.ca/cihiweb/products/nursing_report_2005-2009_en.pdf

CIHI. (2011a). *National Health Expenditure Trends, 1975 to 2011*. Ottawa: Canadian Institute of Health Information. Retrieved from http://secure.cihi.ca/cihiweb/products/nhex_trends_ report_2011_en.pdf

CIHI. (2011b). *Canada's Health Care Providers, 2000 to 2009: A Reference Guide*. Ottawa: Canadian Institute of Health Information. Retrieved from https://secure.cihi.ca/estore/productSeries.htm?locale=en&pc=PCC56

CIHI. (2011c). Draft Pan-Canadian Primary Health Care Electronic Medical Record Content Standard Version 2.0. Ottawa: Canadian Institute of Health Information. Retrieved from http://www.cihi.ca/CIHI-ext-portal/pdf/internet/EMR_V2.0_DATA_EXTRACT_SPEC_EN

Dahl, E. (2002). Health inequalities and health policy: The Norwegian case. *Norsk Epidemiologi 12*, 69-75.

Departementet for Sundhed [Department of Health]. (2010). *Redegørelse for sundhedsreformen* [Report on health reform]. EM 2010/PN. Sagsnr. 2010 - 034099. Nuuk: Naalakkersuisut [Greenland Government]. Retrieved from http://www.peqqik.gl/Sundhedsreformen/~/media/Publikationer/Sundhedsreform_redegoerelse_EM2010/Redegørelse%20om%20Sundhedsreformen_dk_ubilag.ashx

European Commission. (2011). The right prescription for Europe's eHealth. Retrieved from http://ec.europa.eu/information_society/activities/health/policy/index_en.htm

Expert Group on Acute Maternity Services. (2002). *Reference Report.* Edinburgh: Scottish Executive. Retrieved from http://www.sehd.scot.nhs.uk/publications/egas/egas.pdf

Fosse, E. (2008). Norway. In Hogstedt, C., Mobderg, H., Lundgren, B., Backhans, M. (Eds.), *Health for All? A Critical Analysis of Public Health Policies in Eight European Countries* (pp.241-263). Östersund: Swedish National Institute of Public Health. Retrieved from www.fhi.se/PageFiles/4171/R200821_Health_for_all_komp0809.pdf

Foster, M., & Goldsmith, S. (2006). Alaska's $5 billion health care bill: who's paying? *Understanding Alaska Research Summary, No.6*. Retrieved from http://www.iser.uaa.alaska.edu/Publications/researchsumm/RS6_06.pdf .

Fried, N. (2010). Alaska's health care industry. *Alaska Economic Trends, 30(3),* 4-9.Retrieved from http://labor.state.ak.us/trends/mar10.pdf

Frohlick, K.L., Ross, N., & Richmond, C. (2006). Health disparities in Canada today: some evidence and a theoretical framework. *Health Policy,* 79(2-3), 132-143.

Gaski, M., Melhus, M., Deraas, T., Førde, O.H. (2011). Use of health care in the main area of Sami habitation in Norway – catching up with national expenditure rates. *Rural and Remote Health, 11*, 1655. Retrieved from http://www.rrh.org.au/publishedarticles/article_print_1655.pdf

Gísladóttir, E., Þorgeirsdóttir, H., Guðlaugsson, J.Ó., Valdimarsdóttir, M., Jónsson, S.H. & Þórlindsson, Þ. (2009). *Tíðni ofþyngdar og offitu fullorðinna Íslendinga frá 1990 til 2007* [Prevalence of overweight and obesity in the adult population of Iceland 1990-2007]. Reykjavík: Lýðheilsustöð [Public Health Institute of Iceland].

Goldsmith, S. (2008). Understanding Alaska's remote rural economy. *Understanding Alaska Research Summary, No.10.* Retrieved from http://www.iser.uaa.alaska.edu/Publications/researchsumm/UA_RS10.pdf .

Grønlands Hjemmesstyre [Greenland Home Rule Government]. (2007). *Folkesundhedsprogram: Landsstyrets strategier og målsætninger for folkesundheden 2007 – 2012* [Public Health Program: Cabinet's strategies and objectives for public health]. Retrieved from http://www.peqqik.gl/Home/Sundhed/~/media/DS/PDF%20filer/DS/Folkesundhedsprogrammet/Inuuneritta_dk_final_web.ashx

Hacker, J.S. (2002). *The Divided Welfare State: The Battle over Public and Private Social Benefits in the United States*. New York: Cambridge University Press.

Hammond, M. (2006). Road to competency: CHRs and the need for national, competency-based training and credible career paths for Inuit, Métis and First Nations health and wellness workers. Kahnawake, Quebec: National Indian and Inuit Community Health Representative Organization. Retrieved from http://www.niichro.com/2004/pdf/road-to-competency.pdf

Hassler, S., Johansson, R., Sjölander, P., Grönberg, H., & Damber, L. (2005). Causes of death in the Sami population of Sweden, 1961-2000. *International Journal of Epidemiology, 34,* 623-9. Retrieved from http://ije.oxfordjournals.org/content/34/3/623.full.pdf+html

Health Canada. (2011). Non-Insured Health Benefits Program Annual Report 2009/2010. Ottawa: First Nations and Inuit Health Branch, Health Canada.

Health Council of Canada. (2009a). Primary health care teams: A Yukon perspective. Toronto: Health Council of Canada. Retrieved from http://www.healthcouncilcanada.ca/docs/rpts/2009/TeamsInAction_Yukon_Summary.pdf

Health Council of Canada. (2009b). Teams in action: Primary health care teams for Canadians. Toronto: Health Council. Retrieved from http://www.healthcouncilcanada.ca/teamsinaction.pdf

Heilbrigðisráðuneytið [Ministry of Health]. (2008). *Samanburður á rekstri heilsugæslunnar á höfuðborgarsvæðinu við samning um rekstrur heilsugæslustöðvarinnar í Salahverfi* [A Comparison of primary health care within the Capital Area]. Reykjavík: Ministry of Health of Iceland.

Helsedirektoratet. (2011). *SAMDATA spesialisthelsetjenesten* 2010. [Comparable data for the specialist health care sector]. Oslo: Directorate of Health. Retrieved from http://www.helsedirektoratet.no/publikasjoner/samdata-spesialisthelsetjenesten-2010/Sider/default.aspx

Helse- og omsorgsdepartementet. (2008). *Fordeling av inntekter mellom regionale helseforetak*. [Income distribution between regional health authorities]. Norges offentlige utredninger [Norwegian Official Report] NOU 2008:2. Retrieved from http://www.regjeringen.no/pages/2045549/PDFS/NOU200820080002000DDDPDFS.pdf

HRSA. (2011). *Area Resource File, 2009-2010*. Rockville, Maryland: U.S. Department of Health and Human Services, Health Resources and Services Administration. Retrieved from www.arf.hrsa.gov

Irgens, L.M. (2000). The Medical Birth Registry of Norway. Epidemiological research and surveillance throughout 30 years. *Acta Obstetricia et Gynecologica Scandinavica, 79,* 435–439. Retrieved from http://onlinelibrary.wiley.com/doi/10.1034/j.1600-0412.2000.079006435.x/pdf

Johnsen, J.R. (2006). *Health Systems in Transition: Norway.* Copenhagen: WHO Regional Office for Europe on behalf of the European Observatory on Health Systems and Policies. Retrieved from http://www.euro.who.int/__data/assets/pdf_file/0005/95144/E88821.pdf

Joint Authority of Kainuu Region. (2011). English summary. Retrieved from http://www.kainuu.fi/index.php?mid=2_16&la=en

Joossens, L., & Raw, M. (2011). *The Tobacco Control Scale 2010 in Europe*. Brussels: European Network for Smoking Prevention. Retrieved from http://www.ensp.org/sites/default/files/TCS_2010_in_Europe_FINAL.pdf

Kaiser Family Foundation. (2011). Kaiser state health facts. Retrieved from http://www.statehealthfacts.org/

Kamper-Jørgensen, F. (2011). *Midtvejsevaluering af folkesundhedsprogrammet Inuuneritta* [Mid-term evaluation of the public health program Inuuneritta]. Copenhagen: Statens Institut for Folkesundhed [National Institute of Public Health].

Klavus, J., Kinnunen, J., Koistinen, V., Virtanen, M., & Vohlonen, I. (2011). Evaluation of financing of health care by highly decentralised Beveridge model. Consequences of unpredictability of the costs of specialised health care in Finnish municipalities. [unpublished manuscript].

Kokko, S. (2009). Integrated primary health care: Finnish solutions and experiences. *International Journal of Integrated Care, 9,* e86, 1-9. Retrieved from http://www.ncbi.nlm.nih.gov/pmc/articles/PMC2707593/pdf/ijic2009-200986.pdf

Kutzin, J. (2001). A descriptive framework for country-level analysis of health care financing arrangements. *Health Policy, 56*, 171-204.

Lammintakanen, J., Kivinen, T., & Kinnunen, J. (2010). Managers' perspectives on recruitment and human resource development practices in primary health care. *International Journal of Circumpolar Health, 69*, 462-469. Retrieved from http://www.ijch.fi/download.php?abstract_id=1053&file_nro=1

Landlæknisembættið [Directorate of Health]. (2011). *Tölur um notkun á sjúkrahúsaþjónustu, 1999-2009: Legudagar á sjúkrahúsum* [Statistics on hospital utilization: bed-days in hospitals]. Table retrieve from www.landlaeknir.is/pages/1129

Landslægen. (2009). *Årsberetning 2008* [Annual report]. Nuuk: Landslægeembedet [Office of the Chief Medical Officer]. Retrieved from http://dk.nanoq.gl/Emner/Landsstyre/Departementer/Landslaegeembedet/Udgivelser/Aarsberetning/2008.aspx

Lapin sairaanhoitopiri. (2011). UULA: Uudet palvelu- ja toimintamallit Lapissa [New services models and practices in Lapland]. **Retrieved from http://www.lshp.fi/uula**

Liimatta, S. & Paajanen, T. (2007). TEL LAPPI III: Teknologian käyttöönotosta palvelujen kehittämiseen 2001-2007 [From technological initialization to the development of services, 2001-2007]. Rovaniemi: Lapland Hospital District. Retrieved from http://www.lshp.fi/download.aspx?ID=973&GUID=%7BF15ACE7E-862-48 CC-A1C6-18186D3 CBB77%7D

Lund, L. (2011). *Grønland - makroøkonomiske temaer*. Copenhagen: Jurist og Økonomforbundets forlag.
Magga, R. (2010). Saamelaisten sosiaali- ja terveyspalvelujen kehittämisen haasteet Suomessa [Development needs of health and social welfare services for Sami people in Finland]. *Yhteiskuntapolitiikka* [Social Policy] 75, 670 -678. Retrieved from http://www.stakes.fi/yp/2010/6/magga.pdf
Magnussen, J., Vrangbæk, K. & Saltman, R. (Eds). (2009). *Nordic Health Care Systems: Recent Reforms and Current Policy Challenges.* London: Open University Press.
Marchildon, G.P. (2005). *Health Systems in Transition: Canada*. Copenhagen, WHO Regional Office for Europe on behalf on the European Observatory on Health Systems and Policies.
Martelin, T., Koskinen, S., & Valkonen, T. (2006). Mortality. In Koskinen, S., Aromaa, A., Huttunen, J., & Teperi, J. (Eds.), *Health in Finland* (pp.48-51). Helsinki: National Public Health Institute, National Research and Development Centre for Welfare and Health, and Ministry of Social Affairs and Health. Retrieved from http://www.ktl.fi/hif/hif.pdf
Martinussen, P., & Magnussen, J. (2009). Healthcare reform – the Nordic experience. In Magnussen, J., Vrangbæk, K. & Saltman, R. (Eds), *Nordic Health Care Systems: Recent Reforms and Current Policy Challenges.* London: Open University Press.
Ministry of Health and Social Affairs. (2009). *Swedish Strategy for eHealth: Status report 2009.* Retrieved from http://www.regeringen.se/content/1/c6/12/48/02/a97569e9.pdf
Mitton, C., Dionne, F., Masucci, L., Wong, S., & Law, S. (2011). Innovations in health service organization and delivery in northern rural and remote regions: A review of the literature. *International Journal of Circumpolar Health,* 70, 460-472. Retrieved from http://www.ijch.fi/download.php?abstract_id=1130&file_nro=1
MSAH. (2010a). Press release 21.1.2010: Nurses get limited right to prescribe medication to patients in their care. Helsinki: Ministry of Social Affairs and Health. Retrieved from http://www.stm.fi/en/pressreleases/pressrelease/view/1481993#en
MSAH. (2010b). Tiedote 26.3.2010: Tenonlaakson sosiaali- ja terveyspalveluiden kehittämishanke [Press release: Development project of social and health services in Tenonlaakso]. Helsinki: Ministry of Social Affairs and Health. Retrieved from http://www.ouka.fi/kaste/pdf/tenonjoki.pdf
NOMESCO. (2010). *Health Statistics in the Nordic Countries with Data for 2008*. Copenhagen: Nordic Medical-Statistical Committee. Retrieved from http://nomesco-eng.nom-nos.dk/filer/publikationer/Helsestatistik2010.pdf
NOMESCO. (2011). *Social and health indicators database:* Health care expenditure and capacity in the health sector. Retrieved from http://indicators.nom-nos.dk/pxweb/Database/NomNos/databasetree.asp
NST. (2010). *WHO Collaborating Centre for Telemedicine and e-health. Annual Report for 2009.* Tromsø: Norwegian Centre for Integrated Care and Telemedicine. Retrieved from http://img7.custompublish.com/getfile.php/1381609.357.ccyppsqtuw/0710_WHO_CC_2009_annual_report.pdf?return=www.telemed.no
Nystad, T., Melhus, M., & Lund, E. (2008). Sami speakers are less satisfied with general practitioner's services. *International Journal of Circumpolar Health, 67,* 114-121. Retrieved from http://www.ijch.fi/download.php?abstract_ id=50&file_nro=1
OECD. (2008). *OECD Economic Surveys: Iceland 2008*. Paris: Organization for Economic Cooperation and Development. Retrieved from http://www.oecd-ilibrary.org/economics/oecd-economic-surveys-iceland-2008_eco_surveys-isl-2008-en
OECD. (2011). *Health Data 2011.* Paris: Organization for Economic Cooperation and Development. http://stats.oecd.org/index.aspx?DataSetCode=HEALTH_STAT
OECD, Eurostat, & WHO. (2011). *A System of Health Accounts*. Paris: OECD Publishing. doi: 10.1787/9789264116016-en.
Ólafsdóttir, H. (1998). The dynamics of shifts in alcoholic beverage preference: effects of the legalization of beer in Iceland. *Journal of Studies on Alcohol, 59*, 107-17. Retrieved from http://www.jsad.com/jsad/article/The_Dynamics_of_Shifts_in_Alcoholic_Beverage_Preference_Effects_of_the_Leg/489.html

Palosuo, H., Koskinen, S., Lahelma, E., Kostiainen, E., Prättälä, R., Martelin, T., Ostamo, A., Keskimäki, I., Sihto, M., & Linnanmäki, E. (Eds.). (2007). *Terveyden eriarvoisuus Suomessa. Sosioekonomisten terveyserojen muutokset 1980–2005.* [Health inequalities in Finland: Trends in socioeconomic health differences, 1980-2005]. Publications 2007:23. Helsinki: Sosiaali- ja terveysministeriö [Ministry of Social Affairs and Health]. Retrieved from http://www.stm.fi/c/document_library/get_file?folderId=39503&name=DLFE-8726.pdf

Palosuo, H., Koskinen, S., Sihto, M.(2008). Finland. In Hogstedt, C., Mobderg, H., Lundgren, B., & Backhans, M. (Eds.), *Health for All? A Critical Analysis of Public Health Policies in Eight European Countries* (pp. 125-159). Östersund: Swedish National Institute of Public Health. Retrieved from www.fhi.se/PageFiles/4171/R200821_Health_for_all_komp0809.pdf

Parkinson, A.J., Bruce, M.G., Zulz, T., & the International Circumpolar Surveillance Steering Committee. (2008). International Circumpolar Surveillance, an Arctic network for the surveillance of infectious diseases. *Emerging Infectious Diseases, 14,* 18-24. Retrieved from http://wwwnc.cdc.gov/eid/article/14/1/07-0717_article.htm

Persephone, D., Giest, S., & Dumortier, J. (2010). *Country brief: Iceland*. eHealth Strategies. Brussels: European Commission, Directorate-General of Information Society and Media. Retrieved from http://ehealth-strategies.eu/database/documents/Iceland_CountryBrief_eHStrategies.pdf

POSKE (2011). Posken strategia 2010-2015 ja toiminta 2011 [*POSKE Strategy 2010-2015 and activities 2011*]. Rovaniemi: Pohjois-Suomen sosiaalialan osaamiskeskus [The social welfare centre of expertise of Northern Finland]. Retrieved from http://www.sosiaalikollega.fi/poske/julkaisut/tyopaperit-ja-muut-julkaisut/Poske_strat1015_totsu2011.pdf/view

Powers, P. (2011). Integration, healthcare and e-health in the Territories: the tasks ahead for Yukon, the Northwest Territories and Nunavut. *Electronic Healthcare*, 9(4), e21-e31.

Registered Nurses Association of the Northwest Territories and Nunavut. (2005). Practice guidelines for primary health care nurse practitioners: Effective August 2005. Retrieved from http://www.rnantnu.ca/Portals/0/Documents/npguidelines2005.pdf

Rehnberg, C., Magnussen, J., & Luoma, K. (2009). Maintaining fiscal sustainability in the Nordic countries. In Magnussen, J., Vrangbæk, K. & Saltman, R. (Eds), *Nordic Health Care Systems: Recent Reforms and Current Policy Challenges*. London: Open University Press.

Rosstat. (2006). *Itogi Vserossiiskoi perepisi naseleniia 2002 goda*. [2002 All-Russia Population Census]. *Tom 13. Korennye malochislennye narody Rossiiskoi Federatsii* [Numerically small peoples of the Russian Federation]. Moscow: Federal'naia sluzhba gosudarstvennoi statistiki [Federal State Statistics Service]. Statistical tables retrieved from http://www.perepis2002.ru/index.html?id=44

Rosstat. (various years). *Zdravookhranenie v Rossii* 2005,2007, 2009 [Health Care in Russia]. Moscow: Federal'naia sluzhba gosudarstvennoi statistiki [Federal State Statistics Service]. Retrieved from www.gks.ru/wps/wcm/connect/rosstat/rosstatsite/main/publishing/catalog/statisticCollections/doc_1139919134734

Sellers, J M. Lidström, A. (2007). Decentralization, local government, and the welfare state. *Governance, 20*, 609–632. Retrieved from http://onlinelibrary.wiley.com/doi/10.1111/gove.2007.20.issue-4/issuetoc

SKL. (2011). *Verksamhet och ekonomi i landsting och regioner: Statistik om hälso- och sjukvård samt regional utveckling.* [Operations and finances of counties and regions: Statistics on health and medical care and regional development]. Stockholm: Sveriges Kommuner och Landsting [Swedish Association of Local Authorities and Regions]. Retrieved from www.skl.se/vi_arbetar_med/statistik/statistik_ekonomi/verksamhet_och_ekonomi_i_landsting_och_regione

Socialstyrelsen [National Board of Health and Welfare].(2011). *Statistikdatabas: Hälso- och sjukvårdspersonal* [Statistical database: health and health care personnel]. Retrieved from www.socialstyrelsen.se/statistik/statistikdatabas

Solbakk, J.T (Ed.). (2006). *The Sámi People – A Handbook*. Karasjok: Davvi Girji OS.

Sosial- og helsedepartementet. (2001). *Mangfold og likeverd: Regjeringens handlingsplan for helse- og sosialtjenester til den samiske befolkningen i Norge 2002-2005* [Diversity and equality – The Government's action plan for health and social services for the Sami people in Norway]. Retrieved from http://www.regjeringen.no/upload/kilde/shd/pla/2001/0001/ddd/pdfv/140454-mangfold_og_likeverd.pdf

Stasyszyn, R. (2011, February 2). Doctors by numbers. *Yukon News*. Retrieved from http://www.yukon-news.com/news/21517/

Statistics Canada. (2011). *Aboriginal Ancestry (10), Sex (3) and Age Groups (12) for the Population of Canada, Provinces, Territories, Census Metropolitan Areas and Census Agglomerations, 2006 Census - 20% Sample Data* [Cat. No. 97-558-XCB2006013]. Retrieved from www.statcan.gc.ca Home>Census>2006Census: Data Products>Topic-based Tabulations> Aboriginal Peoples>Aboriginal Ancestry

Statistics Greenland. (2010). *Greenland in Figures 2010*. Nuuk: Statistics Greenland. Retrieved from http://www.stat.gl/dialog/main.asp?lang=en&version=2010&link=GF&subthemecode=o1&colcode=o

Statistics Greenland. (2011). *Greenland in Figures 2011*. Nuuk: Statistics Greenland. Retrieved from http://www.stat.gl/publ/en/GF/2011/content/Greenland%20in%20Figures%202011.pdf

Statistics Iceland. (2008). *Spá um mannfjölda 2008–2050* [Population projection]. Hagtíðindi/ Statistical Series 2008-03. Retrieved from http://www.statice.is/lisalib/getfile.aspx?ItemID=8990

Statistics Iceland. (2011). *Mannfjöldaþróun 2010* [Population development]. Hagtíðindi/ Statistical Series 2010-01. Retrieved from http://www.statice.is/lisalib/getfile.aspx?ItemID=12311

Statistics Norway. (2011). *Statbank tables: 03.02 Health services: Specialized health service and municipal health service*. Retrieved from http://statbank.ssb.no/statistikkbanken/Default_FR.asp?PXSid=0&nvl=true &PLanguage=1&tilside=selecttable/MenuSelS.asp&SubjectCode=03

Statistics Norway. (2011). *Statbank tables: 06.01Labour force participation: Health care personnel*. Retrieved from http://statbank.ssb.no/statistikkbanken/Default_FR.asp?Productid=06.01&PXSid=0&nvl=true&PLanguage=1&tilside=selecttable/MenuSelP.asp&SubjectCode=06

Statistics Sweden. (various years). *Statistical Yearbook of Sweden*. Stockholm: Statistika centralbyrån. Available for download from http://www.scb.se/Pages/Product____30937.aspx

Steingrímsdóttir, L., Þorgeirsdóttir, H., & Ólafsdóttir, A.S. (2002). *Hvað borða Íslendingar? Könnun á mataræði Íslendinga 2002* [What do Icelanders Eat? A Dietary Survey 2002]. Research Report no. 5. Reykjavik: Manneldisráð [The Icelandic Nutrition Council]. Retrieved from http://www2.lydheilsustod.is/media/manneldi/rannsoknir/skyrsla.pdf

Straume, K., & Shaw, D.M.P. (2010). Effective physician retention strategies in Norway's northernmost county. *Bulletin of the World Health Organization, 88*, 390-4. Retrieved from http://www.who.int/bulletin/volumes/88/5/09-072686.pdf

Suomen Kuntaliitto. (2011). Information collected from the Internet pages of the Association of Finnish Local and Regional Authorities. Retrieved from http://www.localfinland.fi/en/Pages/default.aspx

Swedish Centre for Epidemiology. (2003). *The Swedish Medical Birth Register – A Summary of Content and Quality*. Research Report 2003-112-3. Stockholm: National Board of Health and Welfare, 2003. Retrieved from http://www.socialstyrelsen.se/Lists/Artikelkatalog/Attachments/10655/2003-112-3_20031123.pdf

Teperi, J., Porter, M.E., Vuorenkoski, L., & Baron, J.F. (2009). *The Finnish health care system: a value-based perspective*. Sitra Reports 82. Helsinki: Sirta, the Finnish Innovation Fund. Retrieved from http://www.sitra.fi/julkaisut/raportti82.pdf

THL. (2011). *Terveydenhuollon menot ja rahoitus 2009*. [Health expenditure and financing 2009]. Statistical Report 14/2011. Helsinki: Terveyden ja hyvinvoinnin laitos [National Institute for Health and Welfare]. Retrieved from http://www.stakes.fi/tilastot/tilastotiedotteet/2011/Tr14_11.pdf

THL. SOTKAnet Statistics and Indicators Bank. (2011). *Social welfare, health care and population data*. Retrieved from http://uusi.sotkanet.fi/portal/page/portal/etusivu/hakusivu?group=248

Turi, A.L., Bals, M., Skre, I.B., & Kvernmo, S. (2009). Health service use in indigenous Sami and non-indigenous youth in North Norway: a population based survey. *BMC Public Health, 9*, 378. Retrieved from http://www.biomedcentral.com/content/pdf/1471-2458-9-378.pdf

Tynkkynen, L-K. (2009). Purchaser-provider models in Finnish health care. *Health Policy Monitor*. Retrieved from http://hpm.org/survey/fi/a14/3

U.S. Census Bureau. (2009). *People without health insurance coverage by selected characteristics:* 2008 and 2009. Retrieved from http://www.census.gov/hhes/www/hlthins/data/incpovhlth/2009/tab8.pdf

U.S. Census Bureau. (2011). Current Population Survey, Annual Social and Economic Supplements. *Table HIB-4. Health insurance coverage status and type of coverage by state all people, 1999 to 2010.* Retrieved from **http://www.census.gov/hhes/www/hlthins/data/historical/HIB_tables.html**

U.S. Census Bureau. (2011). ACS health insurance definitions. Retrieved from : http://www.census.gov/hhes/www/hlthins/methodology/definitions/acs.html

U.S. Census Bureau. (2011). 2010 *Census Summary File 1: QT-P7 - Alaska: Race Reporting for the American Indian and Alaska Native Population by Selected Tribes.* Retrieved from http://factfinder2.census.gov/faces/nav/jsf/pages/index.xhtml

UNDP. (2011). *Human Development Report 2011.* New York: Palgrave Macmillan for the United Nations Development Program. Retrieved from http://hdr.undp.org/en/reports

Vartiainen, P. (2010). Changes and challenges in Finnish health care management. *Society and Economy, 32(1)*, 123-136

Vartiainen, E., Laatikainen, T., Peltonen, M., Juolevi, A., Männistö, S., Sundvall, J., Jousilahti, P,, Salomaa, V., Valsta, L., & Puska, P. (2010). Thirty-five-year trends in cardiovascular risk factors in Finland. *International Journal of Epidemiology, 39*,504-18. Retrieved from http://ije.oxfordjournals.org/content/39/2/504.full.pdf+html

Vilhjálmsson, R., & Sigurðardóttir, G.V. (2003). Bein útgjöld íslenskra heimila vegna heilbrigðisþjónustu [Out-of-pocket health care costs among population groups in Iceland]. *Læknablaðið - The Icelandic Medical Journal, 89*, 25-30. Retrieved from http://www.laeknabladid.is/2003/1/fraedigreinar/nr/1147

Vuorenkoski, L. (20008). *Health system review: Finland*. Health Systems in Transition 10(4). Copenhagen: WHO Regional Office for Europe on behalf of the European Observatory on Health Systems and Policies. Retrieved from http://www.euro.who.int/__data/assets/pdf_file/0007/80692/E91937.pdf

Vuori, J., Kylänen M., & Tritter, J. (2010). Transfer of patients from health care centres to special care services: analysis of travel distances in Nordic countries. *International Journal of Circumpolar Health, 69*, 512-518. Retrieved from http://www.ijch.fi/download.php?abstract_id=1060&file_nro=1

WWAMI Program. (2011). WWAMI turns 40. Retrieved from http://uwmedicine.washington.edu/education/wwami/Pages/default.aspx

WHO. (2011). National health accounts. *Country health information*. Retrieved from www.who.int/nha/country/en/index.html

WHO. (2011). Classifying health workers: Mapping occupations to the international standard classification. Retrieved from http://www.who.int/hrh/statistics/Health_workers_classification.pdf

Young, T.K., & Bjerregaard, P. (2008). *Health Transitions in Arctic Populations*. Toronto: University of Toronto Press.

Young, T.K., & Chatwood, S. (2009). *Public Health Practice in Circumpolar Regions: Lessons for Canada*. Report prepared for the Public Health Agency of Canada. Yellowknife, NT: Institute for Circumpolar Health Research.

Young, T.K., & Makinen, T.M. (2010). The health of Arctic populations: Does cold matter? *American Journal of Human Biology, 22,* 129-133.

Young, T.K., Rawat, R., Dallmann, W., Chatwood, S., Bjerregaard. (2012). *Circumpolar Health Atlas*. Toronto: University of Toronto Press.

### Contributors

**Kathryn Anderson**, MSc, MPH, is President of Pescatore Systems International, an Anchorage-based consulting firm and a PhD candidate at the University of Alaska Fairbanks. She also teaches graduate level courses in marketing at Alaska Pacific University. She serves on the board of directors of several health-related non-profit organizations in Alaska. Her research interests include health care systems, health communication, and program evaluation, and she has published in both computer science and health related journals. In addition to degrees in mathematics, computer science, and public health, she completed the Harvard Business School Advanced Management Program in 1995.

**Tinna Laufey Ásgeirsdóttir**, PhD, is assistant professor of economics and director of the master's program in health economics at the University of Iceland. She has conducted research and taught at several universities, and chaired and served on multiple committees, executive boards and advisory boards. Her main fields of research and teaching are within health economics. She has published on a variety of topics, including the economics of sleep, body weight, and alcohol use. Her research on the health care in Iceland was funded in part by the Icelandic Research Fund (RANNIS).

**Peter Bjerregaard**, MD, PhD, has been Professor of Arctic Health since 1996 at the National Institute of Public Health, University of Southern Denmark, and the Department of Health, Government of Greenland. Early in his career he served as District Medical Officer in Upernavik, in northern Greenland in 1978. Since then he has travelled extensively in Greenland initiating research, collecting data and presenting results. His current research interests are focused on public health in Greenland, particularly inequity in health, social position and health in a lifecourse perspective, and risk factors for cardiovascular disease and diabetes. He is a past president of the International Union of Circumpolar Health. He co-authored with Kue Young *The Circumpolar Inuit* (1998) and *Health Transitions in Arctic Populations* (2008).

**Susan Chatwood**, BScN, MSc, is the founding Executive and Scientific Director of the Institute for Circumpolar Health Research and an Assistant Professor at the Dalla Lana School of Public Health in the University of Toronto. She has spent most of her career in remote and northern communities, working in clinical nursing, public health, and research. Her research interests include circumpolar health systems, climate as a determinant of health; health monitoring strategies; and building sustainable and ethical health research capacity and networks in northern regions. She is dedicated to the development of research infrastructure for the Arctic.

**Juha Kinnunen**, PhD, is Dean of the Faculty of Social Sciences and Business Studies, University of Eastern Finland. After his PhD from the University of Kuopio, he pursued postdoctoral studies at the Massachusetts Institute of Technology in 1991. His main research themes include organisational culture, health care management and policy, nursing administration, evaluation research, and priority-setting in health care. He has been involved several European Union funded projects. At the moment he is a partner in COST Action IS0903 – Enhancing the Role of Medicine in the Management of European Health Systems.

**Johanna Lammintakanen**, PhD, is professor of health management sciences in the Department of Health and Social Management, University of Eastern Finland. She is also a vice director of the department. She obtained her MA in health management sciences and PhD from the University of Kuopio. Her research interests are prioritization in health care as a political issue, human resource management in health care, and development of education in health management sciences.

**Jon Magnussen**, PhD, is Professor of health economics at the Norwegian University of Science and Technology, Trondheim, and head of the Department of Public Health and General Practice. His research interests centres around models for organization and delivery of health services, with particular focus on models for financing and governance. He is the lead editor of *Nordic Health Care Systems: Recent Reforms and Current Policy Changes*", published by McGraw Hill in 2009. His publications cover topics from challenges in the measurement of hospital efficiency to the effect of health care reforms on professional subcultures in medicine.

**Gregory Marchildon**, LLB, PhD, has been Canada Research Chair of Public Policy and Economic History in the Johnson-Shoyama Graduate School of Public Policy, University of Regina, since 2003. Prior to this he served as Executive Director of the Royal Commission on the Future of Health Care in Canada; Cabinet Secretary and Deputy Minister to the Premier of Saskatchewan; and Saskatchewan's Deputy Minister of Intergovernmental Affairs. After completing his PhD at the London School of Economics, he taught at the Johns Hopkins School of Advanced International Studies in Washington, DC, from 1989 until 1994. He has written extensively on health systems, including the Canada profile in the *Health Systems in Transition* series published by the European Observatory on Health Systems and Policies (2005).

**Kue Young**, MD, DPhil, is professor at the Dalla Lana School of Public Health at the University of Toronto, where he holds the endowed TransCanada Chair in Aboriginal Health. He devoted much of his career as primary care physician, public health administrator and academic researcher in northern Canada. For his contributions he was awarded the Order of Canada in 2010, the highest civilian honour in Canada. He is also a Fellow of the Canadian Academy of Health Sciences. He was founding president of the International Network for Circumpolar Health Research and is currently Editor-in-Chief of the International Journal of Circumpolar Health.

Supported by the Nordic Council of Ministers

Health Canada Santé Canada

CIHR IRSC

**International Association of Circumpolar Health Publishers**
Circumpolar Health Supplements
c/o Institute of Health Sciences
PO Box 5000
FI-90014 University of Oulu
FINLAND

**ISSN-L 1797-2361**
**ISSN 1797-2361** (Print)
**ISSN 1797-237X** (Online)
